# Genome-wide association study of dietary intake in the UK biobank study and its associations with schizophrenia and other traits

**DOI:** 10.1038/s41398-020-0688-y

**Published:** 2020-02-03

**Authors:** Maria Niarchou, Enda M. Byrne, Maciej Trzaskowski, Julia Sidorenko, Kathryn E. Kemper, John J. McGrath, Michael C. O’ Donovan, Michael J. Owen, Naomi R. Wray

**Affiliations:** 1 Institute for Molecular Biosciences, University of Queensland, Brisbane, QLD Australia; 2 Medical Research Council Centre for Neuropsychiatric Genetics and Genomics, Division of Psychological Medicine and Clinical Neurosciences, Cardiff University, Cardiff, UK; 3 Vanderbilt Genetics Institute, Vanderbilt University Medical Center, Nashville, Tennessee United States; 4 Max Kelsen, Beyond AI, Brisbane Australia; 5 Esthonian Genome Centre, Institute of Genomics, University of Tartu, Tartu, Estonia; 6 Queensland Brain Institute, University of Queensland, Brisbane, QLD Australia; 7 Queensland Centre for Mental Health Research, The Park Centre for Mental Health, Wacol, QLD Australia; 8 National Centre for Register-Based Research, Aarhus University, Aarhus, Denmark

**Keywords:** Schizophrenia, Genomics

## Abstract

Motivated by observational studies that report associations between schizophrenia and traits, such as poor diet, increased body mass index and metabolic disease, we investigated the genetic contribution to dietary intake in a sample of 335,576 individuals from the UK Biobank study. A principal component analysis applied to diet question item responses generated two components: Diet Component 1 (DC1) represented a meat-related diet and Diet Component 2 (DC2) a fish and plant-related diet. Genome-wide association analysis identified 29 independent single-nucleotide polymorphisms (SNPs) associated with DC1 and 63 SNPs with DC2. Estimated from over 35,000 3rd-degree relative pairs that are unlikely to share close family environments, heritabilities for both DC1 and DC2 were 0.16 (standard error (s.e.) = 0.05). SNP-based heritability was 0.06 (s.e. = 0.003) for DC1 and 0.08 (s.e = 0.004) for DC2. We estimated significant genetic correlations between both DCs and schizophrenia, and several other traits. Mendelian randomisation analyses indicated a negative uni-directional relationship between liability to schizophrenia and tendency towards selecting a meat-based diet (which could be direct or via unidentified correlated variables), but a bi-directional relationship between liability to schizophrenia and tendency towards selecting a fish and plant-based diet consistent with genetic pleiotropy.

## Introduction

Schizophrenia is a chronic mental disorder with typical onset in early adulthood and a lifetime risk of approximately 0.7–0.9%^1^. Affected individuals have a life expectancy that is reduced by an average of 14.5 years relative to the general population^2^. The primary factor contributing to increased mortality is cardiovascular disease (CVD)^3^. Weight gain and obesity, which are common in schizophrenia^4^, are important risk factors for CVD^5^. Notably, evidence of shared genetic factors between schizophrenia and obesity has been reported, but not in the direction expected from epidemiological data. Genetic correlations estimated from genome-wide association study (GWAS) results from independently collected schizophrenia case-control samples and other traits show a significant negative genetic correlation (*r*_g_) of schizophrenia risk with body mass index (BMI) (*r*_g_ = −0.10, s.e. = 0.03, *p* = 0.0002)^6^. There is no evidence for a genetic relationship between schizophrenia and Type 2 diabetes (*r*_g_ = −0.028, s.e. = 0.06, *p* = 0.62) or coronary artery disease (*r*_g_ = −0.0, s.e. = 0.05, *p* = 1.0)^7^. These results imply that if genetic factors also contribute to the associations between metabolic syndrome and schizophrenia, this is a likely complex relationship.

Dietary intake has a causal association with obesity and people with schizophrenia tend to have an unhealthy diet, higher in fat and refined sugar and low in fruit and vegetables^4,8^. We hypothesised that there might be an underlying genetic susceptibility to the self-selected dietary composition in individuals with schizophrenia and that this would be manifest as a significant genetic correlation between schizophrenia and self-selected diet measured in a community sample. A twin study of 18–19-year-olds (*N* = 2865) reported heritability (*h*^2^) estimates for vegetable eating of 54% (95% CI: 47–59%) and for meat or fish-eating of 44% (95% CI: 38–51%)^9^. These estimates may be inflated by shared family environment. Meta-analyses of GWASs for macronutrient intake (i.e., protein, carbohydrate and fat intake) have confirmed associations between consumption of carbohydrates, fat and protein with the fibroblast growth factor 21 (*FGF21*) gene and associations of consumption of protein intake with the fat mass and an obesity-associated locus (*FTO*)^10–12^. Significant genetic correlations between protein intake and BMI (*r*_g_ = 0.23) have been reported, but no significant evidence for genetic correlations between any macronutrient types and schizophrenia (*r*_g_ = < 0.07)^10^. Larger samples are needed to replicate these findings and to elucidate further how diet correlates with other traits at the genetic level.

Our study aimed to investigate (1) genetic influences on dietary intake using GWAS data from the UK Biobank^13^; (2) whether there is shared genetic susceptibility between dietary intake and schizophrenia and (3) if so, whether there was any statistical evidence consistent with a causal relationship between SNPs are bi-directional using Mendelian Randomisation. We also explored genetic correlations of dietary intake with a number of other traits with available GWAS summary statistics.

## Materials/subjects and methods

### Study sample

The United Kingdom Biobank (UKB) is a major community-based longitudinal study with extensive genetic and phenotypic information of over 500,000 participants aged 40–69 years from across the UK during 2006–2010. The study design and sample characteristics have been extensively described elsewhere^13^.

### Ethics statement

This research has been conducted using the UK Biobank resource under application number 12505 and follows UK Biobank’s Ethics and Governance Framework.

### Generic diet questionnaire

All participants completed a generic diet questionnaire (UKB, category:100052) that was used to estimate the average consumption of fruit, vegetables (raw and cooked), fish (oily and non-oily), meat (processed, beef, lamb, pork), bread, cheese, cereal, tea, coffee and drinking water.

**Fig. 1:**
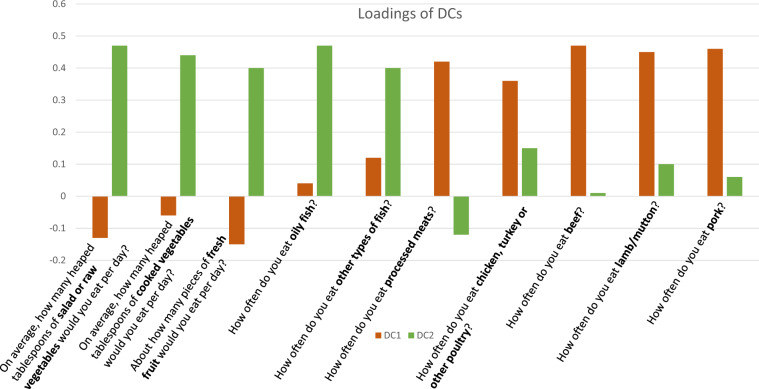
Loadings of diet components (DCs).

We only included responses from individuals at the questionnaire at the first time-point as only a small proportion had completed the questionnaire twice. We standardised the diet questionnaire responses for each item, and we set values that were >3.5 standard deviations from the mean to 3.5 standard deviations. Given the high correlation between question responses, we summarised the questionnaire information by conducting a principal component analysis (PCA)^14^. Since questions about bread, cheese, cereal, tea, coffee and drinking water had low loadings on the components (<0.08), we excluded these questions from the PCA and repeated the PCA using only the questions about fruit, vegetables, fish, and meat consumption. Three eigenvalues were greater than 1. We selected the first two factors, factor 1 (Diet Component 1, DC1), explaining 23% of the variance of the included questionnaire items and representing a meat-related diet (high intake of processed meat, poultry, beef, lamb and pork), and factor 2 (Diet Component 2, DC2), explaining 18% of the variance and representing a fish and plant-related diet (high intake of raw and cooked vegetables, fruit, oily and non-oily fish) (Fig. 1). The third factor, accounted for only 12% of the variance and was not as interpretable as DC1 and DC2 and, therefore, we did not include it in our analysis (Supplementary Fig. 1). A schematic diagram with the number of individuals excluded at each stage is provided in Supplementary Fig. 2 and the distributions of the anthropometric traits of the final sample are provided in Supplementary Fig. 3. By design the phenotypic correlation between DC1 and DC2 is zero; the phenotypic correlations between diet components are in Supplementary Table 1. We excluded from the sample individuals with a BMI that was more or <3 standard deviations from the mean based on their sex and individuals with a diagnosis of anorexia nervosa (ICD-10 code: F50 and ICD-9 code: 307.1) and/or schizophrenia, schizotypal and delusional disorders (ICD-10 codes: F20-F29 and ICD-9 codes: 290–299). Taking into account that data on individuals who follow special diets was only available for 58,985 participants, we did not include this information in our analyses.

### Genotypes

The genotype measures and quality control (QC) of the UKB data have been described extensively by the UKB group^15^ (also see Supplementary Note for more information). We utilised the latest July 2018 genotype release of imputed data from UKB. We only included individuals of White European descent with genetic data. Ancestry was defined using a combination of self-report information on ethnic background and genetic information as described^16^. The total number of markers included was 25,921,788. Principal components were calculated with genotyped variants used by the ukb (identified from the ukb_snp_qc.txt file) and passing additional QC filters (as applied in to unrelated white European set; geno 0.05, pHWE 10-6, MAF > 0.01). Genotyped SNP used by the UKB had already been LD pruned (*r*^2^ < 0.1) and had long-range LD regions removed (Table S12 UKB QC documentation). There were 137,102 SNP included in the analysis. Genetic principal components were calculated for the unrelated white European set using flashPCA^17^ then projected onto the related individuals.

The number of individuals with complete phenotypic and genotypic data was 335,576.

### Statistical analyses

The UK Biobank provided KING kinship coefficients and the fraction of markers, which share no alleles identical-by-descent^15^. We identified likely 100 twin, 12,957 full-sibling, 3354 parent–offspring, 6092 second- and 37,947 third-degree relationship pairs in our European individuals following the procedure outlined in Bycroft et al.^18^. We then estimated the phenotypic correlation (*r*_p_) for each group or relatives and approximated the heritability of DC1 and DC2 (residuals after regression on covariates) within each class as *r*_p_*/a*_R_; where *a*_R_ is the average coefficients of relationship (i.e., monozygotic twins *a*_R_ = 1, full siblings *a*_R_ = 0.5, parent–offspring *a*_R_ = 0.5, second-degree *a*_R_ = 0.25 and third-degree *a*_R_ = 0.125) relatives). This approximation assumes that the *r*_p_ reflects only shared additive genetic contributions. Since close family member share non-additive genetic and family/social networks, contributions from such factors would generate higher *r*_p_*/a*_R_ for close relatives compared to more distant relatives. We also estimated *r*_p_*/a*_R_ for BMI, as a benchmark.

For our GWA analyses, we used the DC1 and DC2 residuals after regressing on covariates of (1) year of birth, (2) sex, (3) month of assessment, (4) assessment centre, (5) batch and (6) 100 genetic PCs. To obtain a better understanding of DC1 and DC2, we also compared their geographical distribution^19^ using the whole sample vs. the sample on unrelated Europeans after regressing out the covariates (Supplementary Figs. 4 and 5). We decided to adjust for 100 genetic PCs given that diet is a trait that is likely to vary within subpopulations.

We used the BOLT-LMM software package^20^ to model the associations between SNPs and the residuals of the two phenotypes, DC1 and DC2. BOLT-LMM uses a mixed model that uses genetic data to account for population structure and relatedness between individuals. The threshold for significance of associations was a *p*-value < 5 × 10^−8^.

We used the FUMA web application to identify independent significant SNPs with a genome-wide significant *p*-value (<5 × 10^−08^) that are in approximate linkage disequilibrium with each other at *r*^2^ < 0.1 and to generate Manhattan and Quintile–Quintile plots and achieve SNP functional annotations^21^.

We performed gene analysis and gene-set analysis with MAGMA v1.6 using FUMA^21^ using the association analysis summary statistics. Taking into account that the UK Biobank imputation used both 1000 genomes and Haplotype Reference Consortium (HRC) reference panels while FUMA only uses 10,000 genomes as a reference panel, it is likely that our gene-set analyses may be based on an incomplete set of variants. Gene expression analysis was obtained from GTEx v6 (https://www.gtexportal.org/home/) integrated by FUMA^22^ (Supplementary Note).

We estimated genetic correlations between schizophrenia^23^ and other complex traits using linkage disequilibrium (LD) score regression through LD Hub v1.9.0 (http://ldsc.broadinstitute.org/centers/)^7^ and GWAS summary statistics. 235 traits were examined using LD hub, and the Bonferroni corrected *p*-value threshold for significance is 0.05/470 = 1 × 10^−4^. For traits that had estimated genetic correlations significantly different from 0 with DC1 or DC2, we used the gsmr R-package to implement Generalised Summary-data-based Mendelian Randomisation to test for bi-directional genetic associations^24^. Heterogeneity in dependent instrument (HEIDI) outlier analyses were implemented to exclude SNPs that have significant pleiotropic effects.

We used the summary-data-based Mendelian randomisation (SMR) software^25^ to examine if the association of an SNP with the phenotype is mediated through gene expression, a tool to help prioritise GWAS results for follow-up functional studies. We used the following summary data expressed: (1) brain-expressed: expression quantitative trait loci (eQTL) meta-analysis data where we tested 7324 probes and DNA methylation quantitative trait loci (mQTL) summary data where we tested 92,867 probes from^26^. The Bonferroni adjusted *p*-value threshold for eQTL analyses was 0.05/7324 = 6.8 × 10^−6^ and for the mQTL was 0.05/92,867 = 5.4 × 10^−7^; (2) blood-expressed: eQTL summary data from the CAGE data set^27^, where we tested 8468 probes and mQTL data from the Brisbane Systems Genetics Study and the Lothian Birth Cohorts of 1921 and 1936 from^28^ where we tested 92,867 probes. The Bonferroni adjusted *p*-value threshold for eQTL analyses was 0.05/8468 = 5.9 × 10^−6^ and for mQTL was 0.05/91,578 = 5.4 × 10^−7^. We conducted a number of sensitivity analyses (Supplementary Note).

## Results

### Diet components (DCs)

In all, 335,576 individuals (46% males) had complete genotypic and phenotypic data and were included in a PCA to generate two independent diet components (DCs) (see Methods, Supplementary Table 2). DC1 represented a meat-related diet and DC2 a fish and plant-related diet **(**Fig. 1 and Supplementary Table 1). DC1 was associated with younger age (*b* = −0.01, *p* < 0.001) (i.e., a 1-year increase in year of birth was associated with a decrease in DC1 by 0.01 standard deviations) and females were more likely to have a lower DC1 score (*b* = −0.38, *p* < 0.001) (Supplementary Table 3). DC2 was also associated with younger age (*b* = −0.03, *p* < 0.001), but in contrast to DC1, females were more likely to have a higher DC2 score (*b* = 0.28, *p* < 0.001) (Supplementary Table 3). Month of questionnaire administration was also significantly associated with DC1 (less meat eating in summer) and DC2 (more fish and plant eating in summer). We report analyses of standardised DC1 and DC2 residuals after regression on covariates, including age, sex, month of questionnaire, assessment centre, genotyping batch and 100 genetic principal components (PCs).

To determine if genetic factors contribute to these DCs, we identified pairs of 1st, 2nd and 3rd-degree relatives and estimated phenotypic correlations (*r*_p_). As expected, *r*_p_ increased with the coefficient of relationship (*a*_R_), and the *r*_p_/*a*_R_ estimates were higher for close relatives. For 3rd-degree relatives, coefficients of non-additive genetic relationship and influences of a shared common environment are expected to be small, so *r*_p_/*a*_R_ provides estimates of the trait heritability (*h*^2^). We estimate *h*^2^ of 0.16 for DC1 and 0.16 for DC2 (Table 1).

**Table 1:** Phenotypic correlations and heritability of DC1 and DC2 within each relationship class.

DC1				DC2		BMI		
	Avg. coefficient of relationship (*a*_R_)	Phenotypic correlation (*r*_p_)	*r*_p_/*a*_R_ (s.e.)	Phenotypic correlation (*r*_p_)	*r*_p_/*a*_R_(s.e.)	Phenotypic correlation (*r*_p_)	*r*_p_/*a*_R_ (s.e.)	*N*^a^
Monozygotic twins	1	0.46	0.46 (0.08)	0.46	0.46 (0.08)	0.75	0.75 (0.09)	100
Full siblings	0.5	0.16	0.32 (0.02)	0.13	0.26 (0.02)	0.25	0.50 (0.02)	12,957
Parent–offspring	0.5	0.16	0.32 (0.04)	0.14	0.28 (0.03)	0.28	0.56 (0.04)	3354
Second-degree relatives	0.25	0.04	0.16 (0.05)	0.04	0.16 (0.05)	0.10	0.40 (0.05)	6092
Third-degree relatives	0.125	0.02	0.16 (0.04)	0.02	0.16 (0.04)	0.07	0.56 (0.04)	37,947

^a^number of pairs; *s.e.* standard error.

### Genome-wide association study

**Fig. 2:**
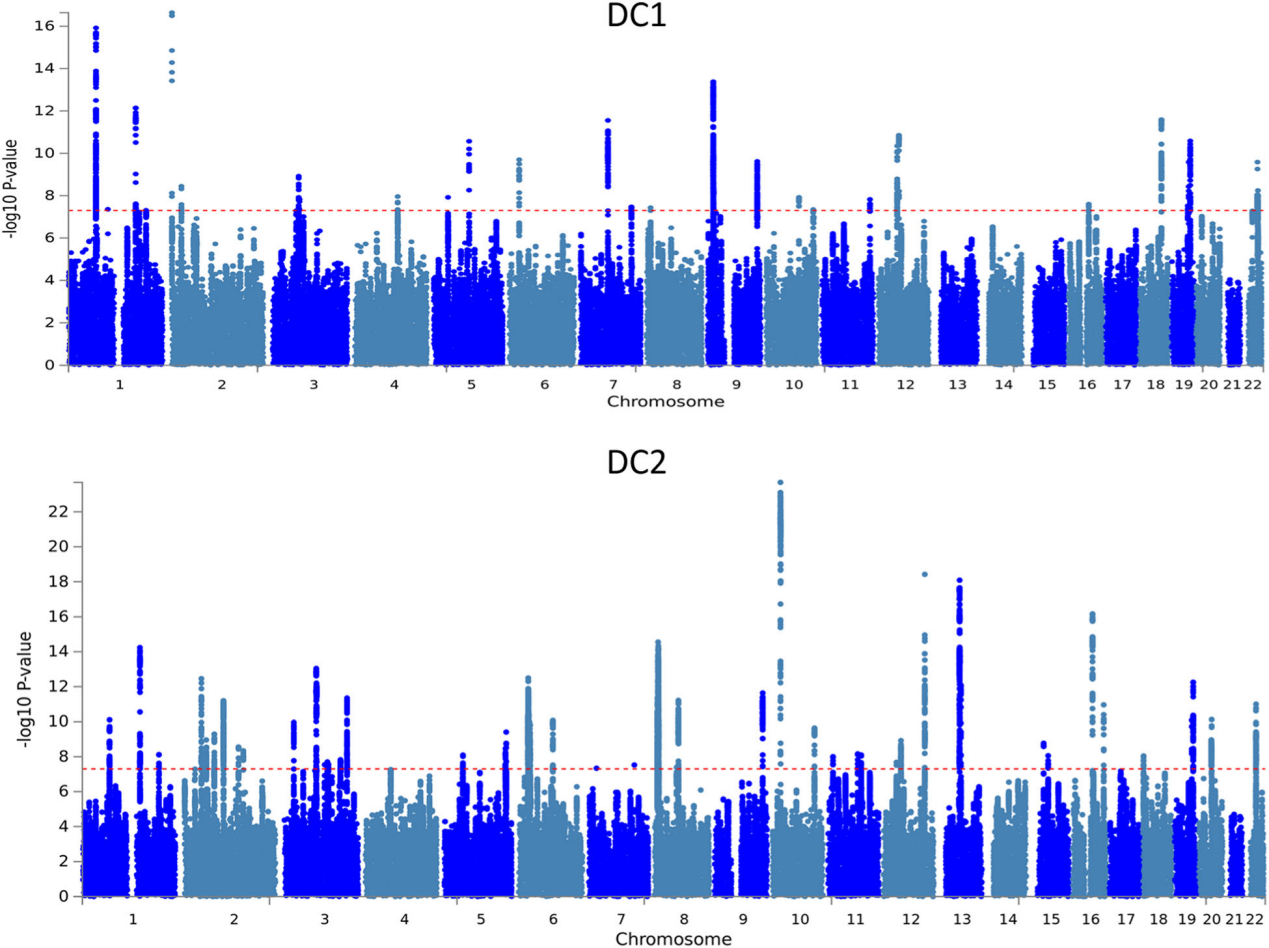
Manhattan plot for diet components 1 and 2.

For DC1, 29 independent SNPs reached genome-wide significance (*p* < 5 × 10^−8^; Fig. 2, Supplementary Table 4, Supplementary Data File, Supplementary Fig. 6). The proportion of variance explained by genome-wide common genetic variants ($h_{SNP}^2$) is 0.055 (s.e. = 0.003), i.e., 31% of the *h*^2^ estimated from 3rd-degree relatives. Among the top-associated loci was the chromosome 19 apolipoprotein E gene (*APOE*, rs429358, *p* = 4.5 × 10^−13^, C allele *b* = −0.02), with the APOE protein a significant cholesterol transporter that has been directly related to low-density lipoprotein cholesterol^29,30^ and mostly known for its associations with Alzheimer’s disease^31^. Here, the C allele, associated with higher risk of Alzheimer’s disease^32^ was negatively correlated with the meat-related diet. Another strongly associated locus was the chromosome 22, rs429358 SNP that maps to the *FGF21* gene, replicating previous GWASs on macronutrient intake^11,12^. This locus was also associated with the individual questionnaire items (Supplementary Table 5) providing further validity on their associations with food consumption. In gene-based analyses there were 41 genes significantly associated with DC1 (Supplementary Table 6), including the neuronal growth regulator 1 (*NEGR1*) gene (*p* = 5.7 × 10^−15^) a BMI-related gene^33–35^. Seven gene-sets reached statistical significance (Supplementary Table 7). The top three gene-sets were related to synaptic plasticity, a process related to memory and learning^36^ that is also found disrupted in people with schizophrenia^37^.

For DC2 ${\mathrm{h}}_{{\mathrm{SNP}}}^2$ was 0.078 (0.004), or 44% of the estimated *h*^2^ estimated from 3rd-degree relatives. There were 63 independent genome-wide significant SNPs (*p* < 5.0 × 10^−8^; Fig. 2, Supplementary Table 8, Supplementary Data File, Supplementary Fig. 6), and 260 genes (Supplementary Table 6) that achieved statistical significance in their respective association analysis tests.

Fat mass and the obesity-associated protein (*FTO*) gene (*p* = 4.4 × 10^−17^), one of the most extensively studied genes in the field of food consumption and obesity, was associated with DC2 at the gene analysis further supporting previous candidate gene studies^38–40^.

Two gene-sets achieved statistical significance (Supplementary Table 7). The top-associated gene-set was the Nikolsky breast cancer 20q11 amplicon gene-set (i.e., genes within amplicon 20q11 that were identified in a copy number alterations study of 191 breast tumour samples) was associated with DC2 (*p* = 1.4 × 10^−08^).

### In silico functional analyses

Integration of association results with GTEx gene expression across 30 tissue types showed that SNP associations were significantly enriched in the brain tissue gene-set for both DC1 and DC2 while DC1 was also significantly enriched in the pituitary gland tissue gene-set (Supplementary Figs. 7 and 8). To investigate in silico putative functional relevance of loci, we integrated eQTL, i.e., SNP gene expression associations) and mQTL summary statistics expressed in the brain and blood tissues^26–28^ using the SMR method^25^. The analyses highlight >200 genes for which association with self-selected diet have the strongest evidence-base (Supplementary Table 9). Results include associations putatively mediated through gene expression for both DC1 and DC2 with neuronal growth regulator 1 (*NEGR1*) and the ribosomal protein L31 pseudogene 12 (*RPL31P12*), both of which have been consistently associated with BMI^41,42^, educational attainment^43^, intelligence^44^ and major depressive disorder^45^. In addition SMR association analyses link DC1 and Histone Cluster 1 H2B Family Member F (*HIST1H2BF*) that has been associated with hip circumference^46^ and body height^47^, and DC2 with IKAROS family zinc finger 3 (*IKZF3*) gene that has been associated with inflammatory bowel disease^48^ and family with sequence similarity 167 member A (*FAM167A*) gene that has been associated with systemic lupus erythematosus^49^.

### Cross-trait analyses

**Fig. 3:**
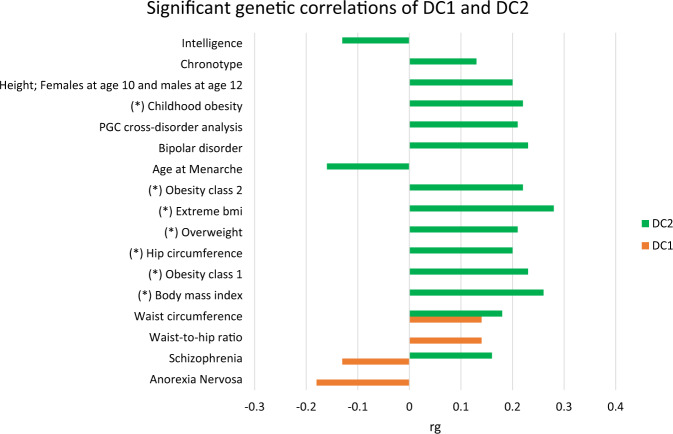
Significant genetic correlations of DC1 and DC2.

**Fig. 4:**
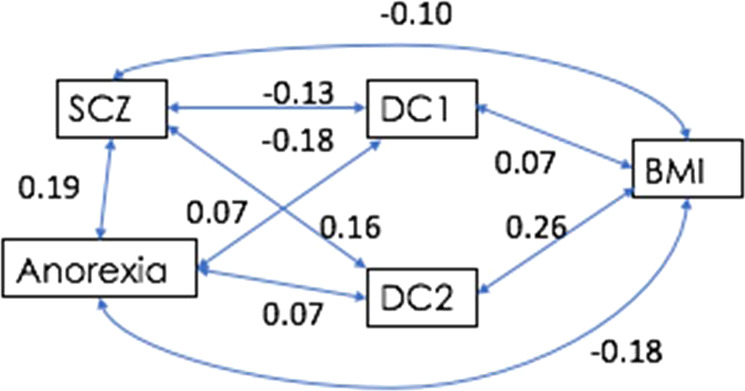
Genetic correlations between DC1, DC2, BMI, schizophrenia.

Genetic correlations between DC1 or DC2 with other traits estimated from publicly available GWAS summary statistics (Figs. 3 and 4, and Supplementary Table 10, Supplementary Table 11) show significant estimates with schizophrenia and a number of other traits. In particular, there was a negative genetic correlation between schizophrenia and DC1 (*r*_g_ = −0.13, s.e. = 0.03, *p* = 2.0 × 10^−6^) and positive genetic correlation between schizophrenia and DC2 (*r*_g_ = 0.16, s.e. = 0.03, *p* = 9.4 × 10^−8^). Since DC1 and DC2 are orthogonal (i.e., phenotypically uncorrelated), they were also genetically uncorrelated (*r*_g_ = −0.03, s.e. = 0.03, *p* = 0.30).

DC1 showed a significant negative genetic correlation with anorexia nervosa (*r*_g_ = −0.18, s.e. = 0.03, *p* = 5.02 × 10^−08^) and positive genetic correlations with waist circumference (*r*_g_ = 0.14, s.e. = 0.03, *p* = 3.8 × 10^−06^) and waist-to-hip-ratio (*r*_g_ = 0.14, s.e. = 0.03, *p* = 5.13 × 10^−06^) (Fig. 3).

DC2 was positively correlated to bipolar disorder (*r*_g_ = 0.23, s.e. = 0.04, *p* = 6.9 × 10^−08^) , chronotype (*r*_g_ = 0.13, s.e. = 0.03, *p* = 4.2 × 10^−05^), BMI (*r*_g _ =  0.26, s.e. = 0.03, *p*  =  5.16  × 10^−19^) and other BMI-related traits while negative genetic correlations were found with age at menarche (*r*_g_ = −0.16, s.e. = 0.03, *p* = 4.9 × 10^−09^) and intelligence (*r*_g_ = −0.13, s.e. = 0.03, *p* = 4.2 × 10^−05^). We also conducted genetic correlations analyses for the DC1 per sex separately but the results were similar for both sexes (Supplementary Tables 16 and 17).

### Mendelian randomisation (MR) analyses

Given the significant genetic correlations between the DCs and other traits we used MR analyses (via the GSMR method^50^) to investigate if there is statistical evidence consistent with uni- or bi-directional relationships between the correlated traits.

MR analyses between DC1 and schizophrenia were weakly significant when DC1 was an outcome, where for 1 standard deviation liability to schizophrenia there was a reduction of 0.03 standard deviations of DC1 (*b*_DC1|SCZ_ = −0.03, s.e. = 0.003, *p* = 2 × 10^−02^) but were not significant when DC1 was the exposure (Table 2).

**Table 2:** Generalised summary-data-based Mendelian randomisation (GSMR) results.

	Outcome					Exposure			
	DC1					DC1			
Exposure	***b***	**s.e.**	***p***	*N*_SNP_	Outcome	***b***	**s.e.**	***p***	*N*_SNP_
Schizophrenia^a^	**−0.03**	**0.00**	**2.0E-02**	**118**	Schizophrenia	0.98	0.16	8.9E-01	24
Anorexia Nervosa	n/a	n/a	n/a	n/a^b^	Anorexia Nervosa^a^	0.49	0.38	6.0E-01	26
Waist hip ratio	−0.01	0.02	6.4E-01	31	Waist hip ratio	0.06	0.06	3.2E-01	21
	**DC2**					**DC2**			
	***b***	**s.e.**	***p***	*N*_SNP_		***b***	**s.e.**	***p***	***N***_**SNP**_
Schizophrenia^a^	**0.05**	**0.00**	**3.2E-06**	**118**	Schizophrenia^a^	**1.43**	**0.10**	**1.0E-03**	**46**
Bipolar^a^	**0.05**	**0.01**	**6.0E-03**	**16**	Bipolar^a^	**1.92**	**0.13**	**3.3E-07**	**46**
Intelligence	**−0.04**	**0.02**	**5.0E-02**	**13**	Intelligence	−0.08	0.06	2.0E-01	38
Age at Menarche	−0.01	0.01	1.7E-01	71	Age at Menarche	**−0.18**	**0.06**	**2.0E-03**	**45**
Chronotype^a^	0.08	0.03	1.6E-01	11	Chronotype^a^	1.07	0.04	7.0E-02	50
BMI	**0.15**	**0.01**	**4.8E-39**	**82**	BMI	0.07	0.04	6.0E-02	44

*DC* diet component, *N*_SNP_ number of single-nucleotide polymorphisms, *b*; ^a^beta coefficient on the liability scale, in bold are statistically significant associations.

MR analyses between DC2 and schizophrenia were bi-directionally significant (Table 2). Particularly, when DC2 was an outcome, for 1 standard deviation liability to schizophrenia there was an increase of 0.05 standard deviations of DC2 (*b*_DC2|SCZ_ = 0.05; s.e. = 0.00, *p* = 3.2 × 10^−06^, number of independent SNP instruments (*N*_SNP_) = 118). When DC2 was an exposure, for 1 standard deviation change in DC2 the odds of liability to schizophrenia increased 1.43 fold (*b*_SCZ|DC2_ = 1.43, s.e. = 0.10, *p* = 1 × 10^−03^, *N*_SNP_ = 46).

MR analyses between DC1 and waist hip ratio and DC1 and anorexia nervosa were not significant.

Interestingly, BMI was associated with DC2 when DC2 was an outcome (*b*_DC2|BMI _= 0.15, s.e. = 0.01, *p* = 4.9 × 10^−39^, *N*_SNP_ = 82), but not when DC2 was an exposure (*b*_BMI|DC2 _= 0.07, s.e. = 0.04, *p* = 0.06, *N*_SNP_ = 44) (Supplementary Fig. 9). Thus, there is no evidence for a high consumption of fruit, vegetables and fish being causally related to an increase in BMI, consistent with observational studies^51^ and dietary population guidelines^52^ rather the observed correlations are a result of tendency to have increased consumption of fruit, vegetables and fish as a (direct or indirect) consequence of high BMI, which may reflect that, in general, individuals with high BMI consume larger quantities of all types of food.

## Discussion

### Dietary intake

Our study is the largest to investigate the genome-wide associations of dietary intake. We applied PCA to diet questionnaire item responses and identified two independent diet components, with high DC1 representing high-meat consumption and high DC2 reflecting high consumption of fish and plant-related products. This analysis enabled us to undertake GWAS analyses of quantitative measures of self-reported dietary intake.

Variation between people in dietary intake as represented by DC1 and DC2 is predominantly driven by non-genetic factors, consistent with it being primarily influenced by a variety of socioeconomic and psychological factors, including lifestyle, culture and health beliefs^53^. Nonetheless, both DC1 and DC2 were moderately heritable (both 16% Table 1) in line with reports from twin studies^9,54^ and consistent with animal studies that imply biological driving forces underpinning self-selection of diet^55^. The proportion of variance explained by genome-wide SNPs was 6% and 8% for DC1 and DC2. Hence this common variation explains 31% and 44% of the estimated heritability, respectively. Twenty-nine independent loci passed the GWA threshold for DC1 and sixty three for DC2. The *FGF21* gene reached GWA significance in the gene-wide analyses for both DC1 and DC2 (which were phenotypically and genetically uncorrelated), replicating previous GWA studies on macronutrient intake^11,12^. Moreover, 4 out of 7 brain mQTL SNPs and 6 out of 12 blood-expressed mQTL SNPs were associated with DC1. Five mQTL SNPs and one eQTL SNP expressed in the brain and eight mQTL SNPs expressed in the blood were associated with DC2 were at the 19q13.3 locus, including the izumo sperm-egg fusion 1 (*IZUMO1*) gene, the MEF2 activating motif, and SAP domain containing transcriptional regulator (*MAMSTR*) gene and the RAS-interacting protein 1 (*RASIP1*) gene supporting a role of the 19q13.3 locus in diet intake^10–12^.

### Dietary intake and schizophrenia

Our primary hypothesis that there would be a genetic correlation between dietary intake and schizophrenia was supported. It is important to note that we chose to examine food type consumption rather than macronutrient intake, a decision informed by a published GWA meta-analysis (*n* = 91,114) that did not find evidence of a phenotypic relationship of macronutrient intake and schizophrenia^10^. Based on the diet questionnaire data available, our DCs reflect self-selected diet composition and quantity.

We found that genetic factors contributing to high DC2 values, indicating higher consumption, mainly, of fish (oily and non-oily) and cooked vegetables were positively and significantly correlated with genetic factors associated with schizophrenia (regardless of whether BMI was included as a covariate in analyses) (Supplementary table 12). On first consideration, taking into account wealth of evidence indicating that higher consumption of fruit and vegetables is related to a variety of positive health and psychological outcomes, including decreasing risk for cancer and heart disease^56,57^, lower incidence of depression^58^, better emotional health^59^, greater happiness and life-satisfaction^60,61^, the direction of our association of dietary intake and schizophrenia seems unexpected. However, our results suggest a more complex relationship between self-selected diet and schizophrenia risk, and that the reported negative health consequences of schizophrenia are likely to be consequences of factors associated with the illness (e.g., lack of access to care, economic disadvantage) or drug treatment (where drugs targeting schizophrenia pathways could impact DC2 pathways). Mendelian Randomisation analyses indicated that while results were consistent with schizophrenia being related with a reduction of DC1, that the relationship between schizophrenia and DC2 is more likely to reflect pleiotropy.

### Dietary intake and BMI

Given the wording of the dietary questions used to generate the DCs, we expect that the DCs reflect both dietary compositions and quantity. Hence, a relationship with BMI and other obesity-related traits is expected. Indeed, we found a strong genetic correlation between DC1 and waist circumference and genetic correlations between DC2 and many BMI-related traits, including obesity and being overweight. We decided not to include these traits as covariates to our analysis to avoid potential biases arising from using residuals^62^ and from conditioning on heritable covariates^63^. However, as a sensitivity analysis, we re-ran our analyses adjusting DC1 and DC2 for BMI and then, as predicted^63^ observed a significant genetic correlation between DC2 and BMI (Supplementary Tables 13 & 14), and an induced correlation between the DC1 and BMI; these results affirmed our decision not to adjust for BMI at the phenotypic level. We also used Genome-Wide Inferred Study (GWIS)^64^, where we conditioned DC1 on waist circumference and DC2 on BMI; this analysis forces the genetic correlation between the conditioned trait (e.g., DC2 conditioned on BMI) and the conditioning trait (e.g., BMI) to be zero. In that case, all the previously significant genetic correlations between DC1, DC2 and the other traits became non-significant (Supplementary Table 15), even though the overlap in liberally associated SNPs was low (Supplementary Fig. 9). Our results illustrate the complex relationship between the DCs and obesity-related traits, with DC1 and DC2 independent dimensions; the former related to waist circumference and waist/hip ratio, and the latter associated with BMI. The relationship between DC2 and BMI is particularly complex. The genetic correlation is in the direction of genetic factors associated with eating more fish, fruit and vegetables as associated with higher BMI.

### Implications

The low-heritability estimates of DC1 and DC2 attest to dietary intake being a mostly environmental, and therefore potentially modifiable, behavioural trait^65^. Although we observed genetic correlations between genetic liability to dietary choice, schizophrenia, and bipolar disorder, the patterns of results from the MR tests suggested this is likely to be primarily the result of genetic pleiotropy. In other words, some of the alleles that influence dietary choice also influence liability to psychiatric disorder, but those alleles do not do so by their effects on diet itself. Nevertheless, given that diet and nutrition affect biological processes potentially involved in psychiatric (and definitely in some physical disorders), such as inflammation^66^, oxidative processes^67^, and brain plasticity^68^, it is important to further examine potentially shared pathways between dietary intake and disease to gain a better understanding of the underlying biology. Although the direction of the association results between the DCs and schizophrenia was unexpected, similar seemingly paradoxical results have been type found for 2 diabetes (T2D). Zhu et al.^50^ recently used GSMR Mendelian randomisation to report a negative association between BMI and type 2 diabetes (T2D), despite BMI being a known risk factor (confirmed through randomised control trial^69^). They interpret the relationship between BMI and T2D as a complex mixture of causality, reverse causality and pleiotropy. It seems that this broad interpretation is likely to represent the relationship between dietary intake, BMI and schizophrenia.

### Limitations

We used a self-report questionnaire to assess dietary intake. Although this is the typical assessment method used in large population-based studies^70^, there is evidence that higher BMI is associated with under-reporting of the quantity of food consumption^71,72^ and that individuals tend to under-report unhealthy foods and over-report consumption of fruit and vegetables^73^. The assessment we used is subjective and reflects a significant limitation of population and community studies of nutritional science in general^74^. Another limitation is that diet changes over time^75^. The UK biobank participants are not representative of the population in certain sociodemographic characteristics^76^. Finally, ascertainment bias could lead to collider bias (i.e., spurious associations between two unrelated traits, which is driven by each being associated with a trait that influences participation in the study)^77^. Therefore, our findings need to be tested in other cohorts.

## Conclusions

Although the heritability of diet intake measures is low, we identified many independent genome-wide significant loci associated with our two DC traits, genetic correlations, as well as possible causal and shared genetic pathways with schizophrenia and many other traits. Our study adds evidence-based results to the growing recognition of the need of a holistic approach in the context of disorders of the brain. Further studies are needed to help gain a better understanding on the role of diet, nutrition and metabolic traits in disease onset, disease progression and treatment.

## Supplementary information

Supplementary Note

Supplementary Tables

Supplementary Figures

Regional association plots
