# Supplementary material for: Genome-wide association study of dietary intake in the UK biobank study and its associations with schizophrenia and other traits
Source: Transl Psychiatry. 2020 Feb 3;10:51. doi: 10.1038/s41398-020-0688-y (PMC7026164; doi:10.1038/s41398-020-0688-y)
Supplement: Supplementary file 1 — Supplementary Note [file 41398_2020_688_MOESM1_ESM.docx]

**Supplementary material**

**Supplementary methods**

Genotypes

488,377 UK Biobank participants were genotyped using two similar genotyping arrays. 49,950 participants were genotyped using the Applied BiosystemsTM UK BiLEVE AxiomTM Array by Affymetrix (807,411 markers), and 438,427 participants were genotyped using the Applied BiosystemsTM UK Biobank AxiomTM array (825,927 markers). Marker and sample-based QC procedures were applied using PCA to account for population stratification. Testing was conducted for batch and plate, sex, and array effects, as well as departures from Hardy Weinberg equilibrium and discordance between control replicates. Poor quality markers and samples were set to missing. The final number of markers included from both arrays was 805,426 and were imputed with IMPUTE2^1^.

We utilized the latest July 2018 genotype release of imputed data from UKB.

We only included individuals of White European descent with genetic data. Ancestry was defined using a combination of self-report information on ethnic background and genetic information as described^2^. The total number of markers was 25,921,788.

Gene-based test and gene-set analysis

In the gene-based test, SNPs are considered in aggregation to the level of genes (19,436 protein-coding genes) to test the cumulative association signal of the genes with the phenotype. In the gene-set analysis, the genes are categorized in groups based on their certain biological and functional traits, and P values result from a competitive analysis that tested whether genes in one gene set are more strongly associated with the phenotype than other gene sets. Gene-set analysis examined 10894 gene sets. The threshold of statistical significance for this analysis is Bonferroni corrected (p< 5.0 x 10^-6^).

Gene-expression

Gene expression in a specific tissue type and genetic associations were tested, and average gene-expression per tissue type was used as a covariate. The MAGMA gene-property test^3^ was performed to estimate average expression of 30 general tissue types and 53 specific tissue types conditioning on average expression across all tissue types.

**Sensitivity analyses**

We conducted the following sensitivity analyses:

1. We conducted GWAS and genetic correlation analyses for each questionnaire item that was included in the PCA (**Supplementary tables 5 and 12**).
2. To further examine the relationships between the DCs and BMI, we repeated the GWAS, genetic correlation analyses and gsmr analyses, by adjusting the DCs for age, sex, chip, batch, educational attainment, Townsend Deprivation Index (TDI), and BMI (**Supplementary tables 13 and 14**), because educational attainment, TDI and BMI are also strongly related to schizophrenia^4^.
3. To examine the extent to which the DCs capture SNPs related to BMI, we also run a Genome Wide Inferred Study (GWIS) (**Supplementary Table 15**).
4. Taking into account the reported differences in dietary intake between males and females, we examined whether the associations between genes and the two DCs differ by sex. Specifically, we repeated the GWAS and conducted genetic correlations analyses for the DCs per sex separately (**Supplementary tables 16 and 17**). It is likely that the composition of the DCs themselves differs between males and females, and although this is an interesting question, it is outside the focus of our study.

**Results of sensitivity analyses**

GWAS results

**Individual traits**

A varying number of independent GWAS SNPs was associated with the individual dietary intake items (**Supplementary Table 5**). Among the top three groups of SNPs associated with DC1, rs66495454 was also associated with processed meat (p=4.3 x 10^-14^) and rs429358 was also associated with lamb consumption (5 x 10^-12^). Among the top three groups of SNPs associated with DC2, rs946711 was also associated with fruit consumption (p=4.7 x 10^-20^) and rs35287743 was also associated with oily-fish consumption (p=7.2 x 10^-17^) and pork consumption (p=7.2 x 10^-17^).

Genetic correlation analyses

**Individual traits**

The total observed scale heritability ranged between 0.03 (se=0.002) for poultry and to 0.07 (se=0.003) for fruit consumption (**Supplementary Table 12**).

Schizophrenia was negatively correlated with beef consumption (rg=-0.16, p=1.3 x 10^-6^), pork consumption (rg=-0.20, p=4.5 x 10^-09^) and positively correlated with cooked vegetables (rg=0.17, p=2.3 x 10^-07^), oily fish consumption (rg=0.22, p=7.1 x 10^-15^), and non-oily fish consumption (rg=0.20, p=8.4 x 10^-7^). BMI and BMI related traits were also highly correlated with a number of traits.

Our sensitivity analyses using GWAS results for DCs that included BMI as a covariate, generated a statistically significant correlation between DC1 and BMI, which points towards an bias due to adjustment for covariates^5^.

When we conditioned DC1 on waist –hip-ratio and DC2 on BMI using GWIS, all genetic correlations disappeared.

**References**

1 Bycroft, C. *et al.* The UK Biobank resource with deep phenotyping and genomic data. *Nature* **562**, 203-209, doi:10.1038/s41586-018-0579-z (2018).

2 Yengo, L. *et al.* Imprint of assortative mating on the human genome. *Nature Human Behaviour* **2**, 948-954, doi:10.1038/s41562-018-0476-3 (2018).

3 de Leeuw, C. A., Mooij, J. M., Heskes, T. & Posthuma, D. MAGMA: generalized gene-set analysis of GWAS data. *PLoS Comput Biol* **11**, e1004219, doi:10.1371/journal.pcbi.1004219 (2015).

4 van Os, J. & Kapur, S. Schizophrenia. *Lancet* **374**, 635-645, doi:S0140-6736(09)60995-8 [pii]

10.1016/S0140-6736(09)60995-8 (2009).

5 Aschard, H., Vilhjalmsson, B. J., Joshi, A. D., Price, A. L. & Kraft, P. Adjusting for heritable covariates can bias effect estimates in genome-wide association studies. *Am J Hum Genet* **96**, 329-339, doi:10.1016/j.ajhg.2014.12.021 (2015).
