# Supplementary material for: Genome-wide association study of dietary intake in the UK biobank study and its associations with schizophrenia and other traits
Source: Transl Psychiatry. 2020 Feb 3;10:51. doi: 10.1038/s41398-020-0688-y (PMC7026164; doi:10.1038/s41398-020-0688-y)
Supplement: Supplementary file 3 — Supplementary Figures [file 41398_2020_688_MOESM3_ESM.docx]

Supplementary figures

**Supplementary Figure 1.** Loadings of the third principal component derived from the diet questionnaire


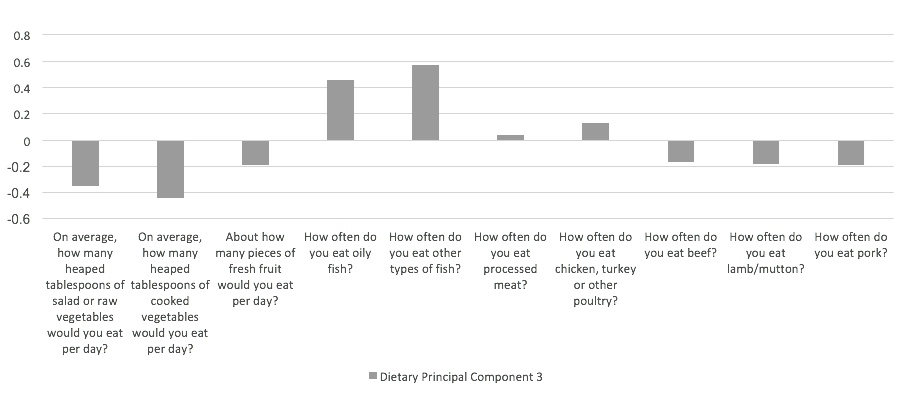


**Supplementary Figure 2:** Schematic diagram with number of individuals at each stage of selection. Percentages represent percentage of people removed per category (e.g., 11% of individuals removed out of 405,238 individuals, 7% of individuals removed out of 360,315 individuals)


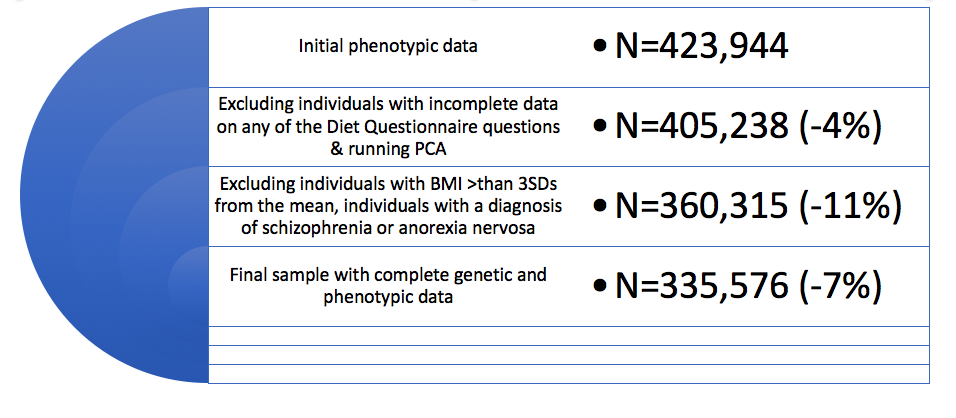


**Supplementary Figure 3.** Distributions of BMI, and waist hip ratio. Before restricting to individuals within 3 standard deviations from the mean, BMI ranged from 12.12 to 74.68, and Waist Hip Ratio (WHR) from 0.2 to 2.97. In our final sample, BMI ranged from 13.61 to 42.46, while WHR values ranged from 0.2 to 2.13.


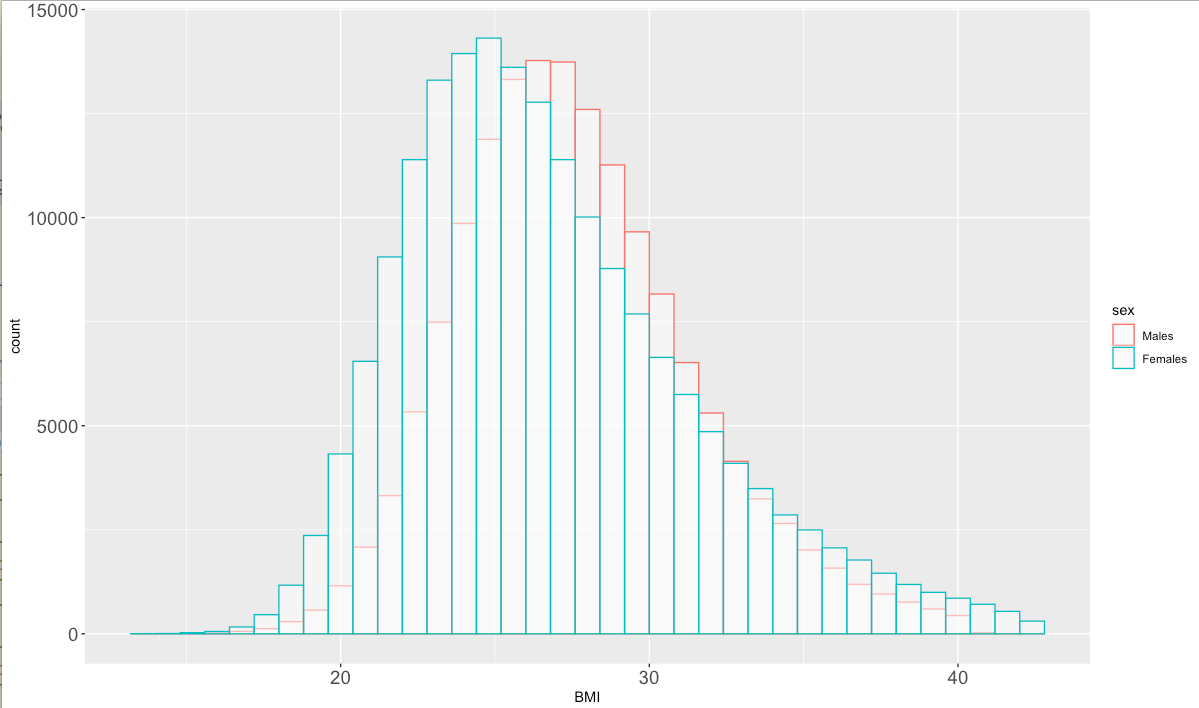


.


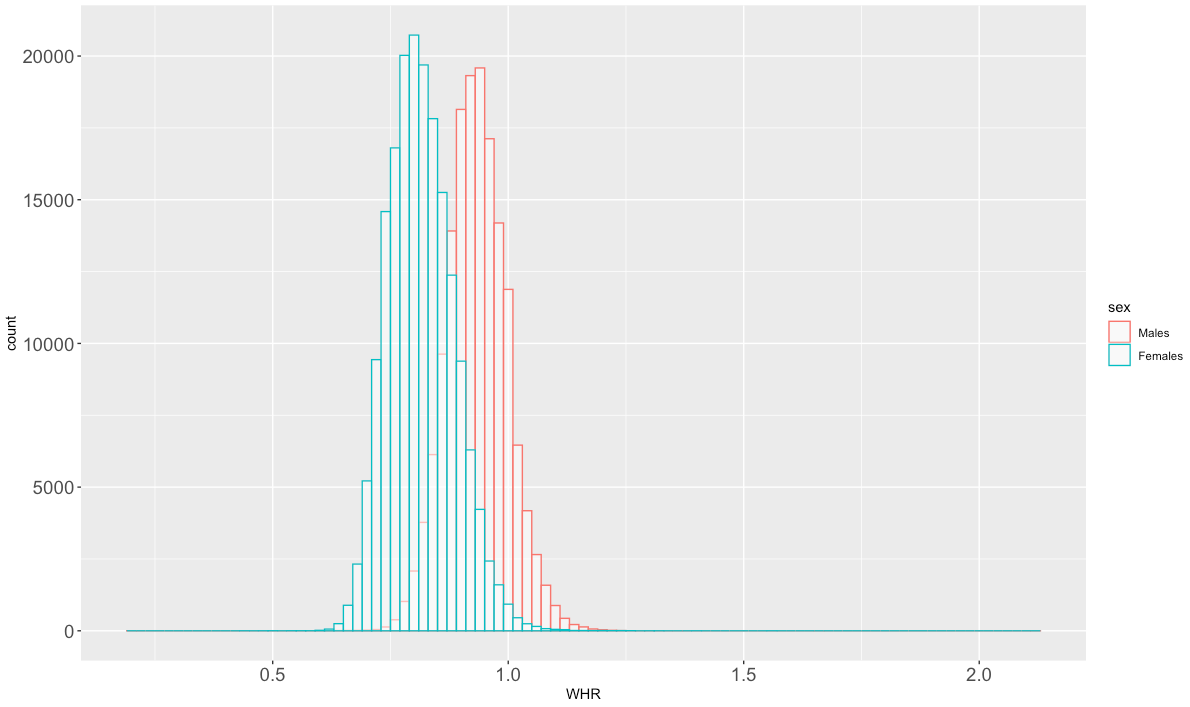


**Supplementary Figure 4.** Geographical distribution of Diet Component 1 before and after adjustment for confounders.

1. Diet Component 1 (whole sample) - Moran coefficient=0.78, p=1x10^-4^
2. Diet Component 2 (unrelated individuals and adjusted for confounders) – Moran coefficient=0.1, p=1.7 x 10^-3^

A

B


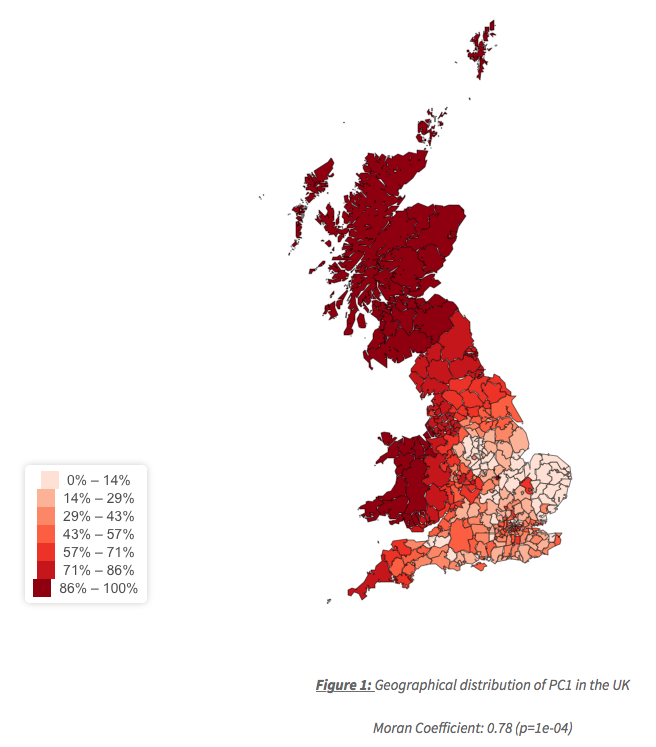

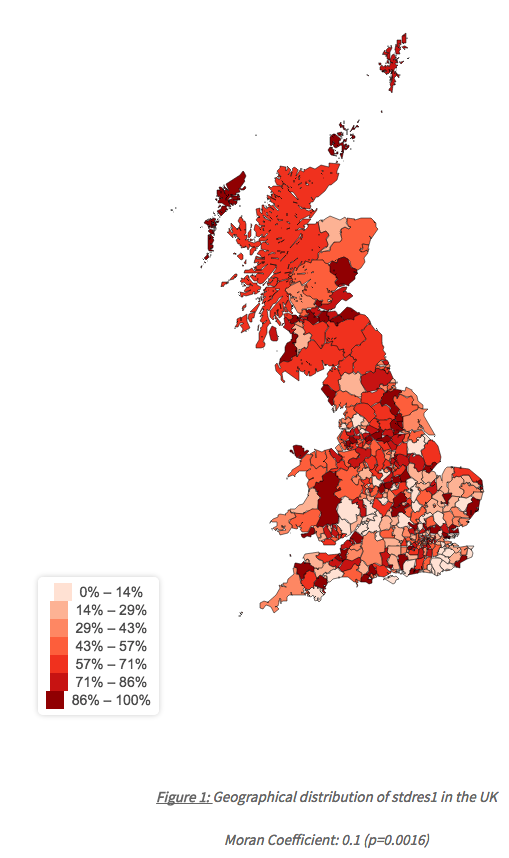

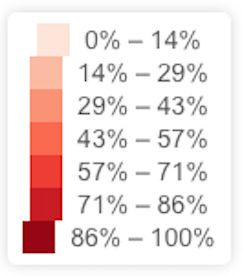


**Supplementary Figure 5.** Geographical distribution of Diet Component 2 before and after adjustment for confounders

1. Diet component 2 (whole sample) – Moran coefficient=0.83, p=1 x 10^-4^
2. Diet component 2 (unrelated Europeans and adjusted for confounders) – Moran coefficient=0.08, p=1.3 x 10^-2^

B


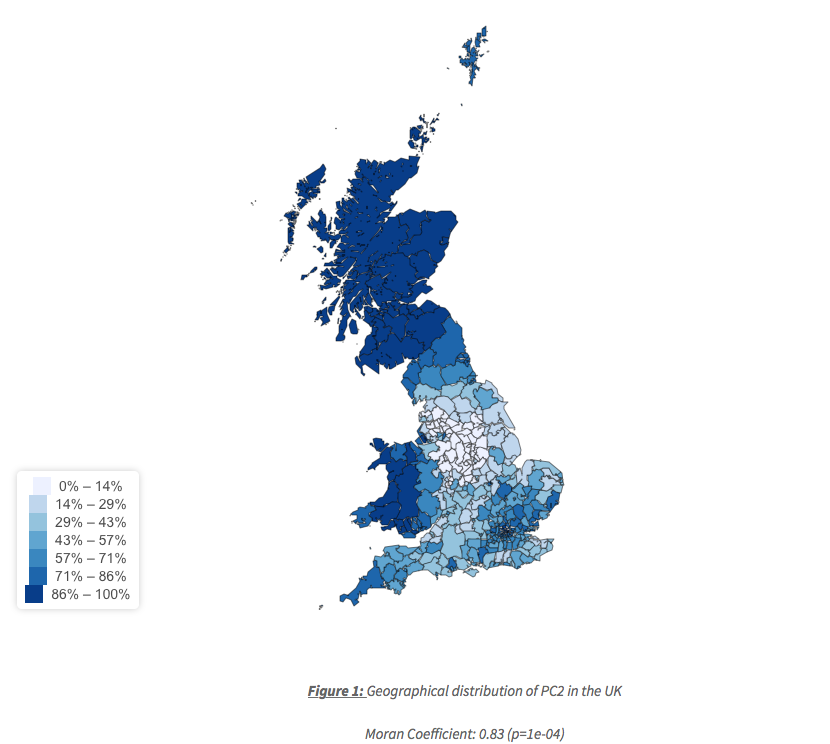

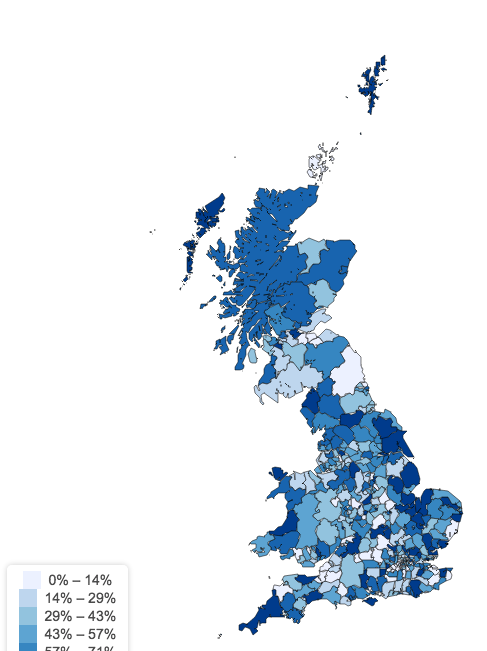

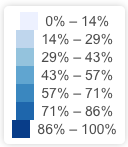


A

**Supplementary Figure 6:** Q-Q plots for all Diet Components

DC2

DC1


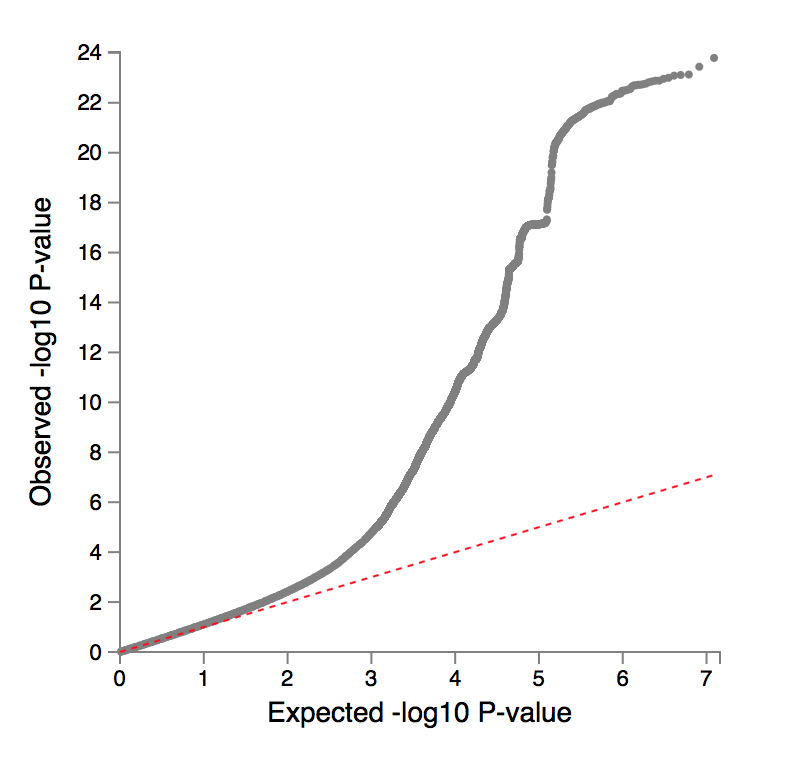

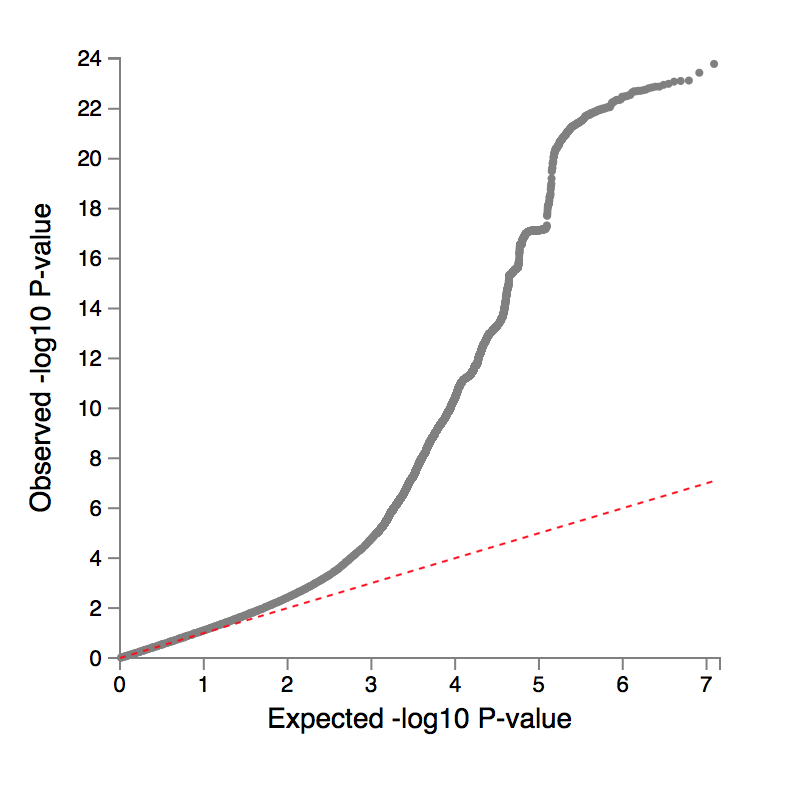


DC2: Lambda GC=1.43, Intercept=1.015(0.009)

DC1: Lambda GC=1.31, Intercept=1.029(0.008)

**Supplementary Figure 7:** General and specific tissue expression (DC1) – Graph is output from FUMA


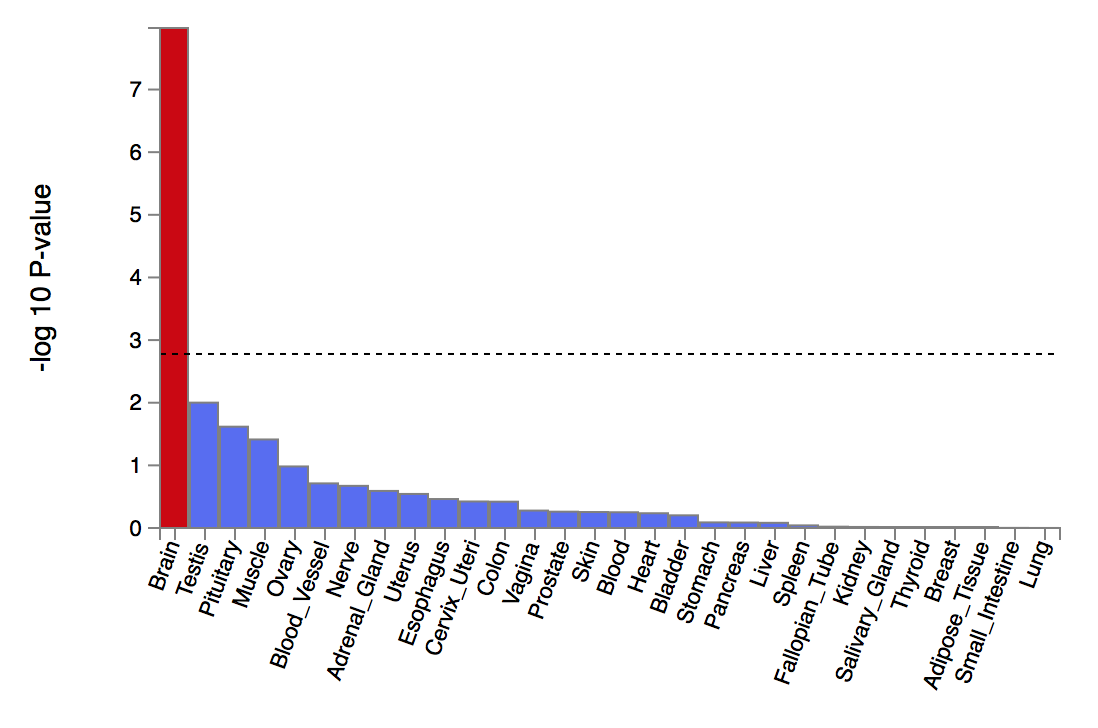


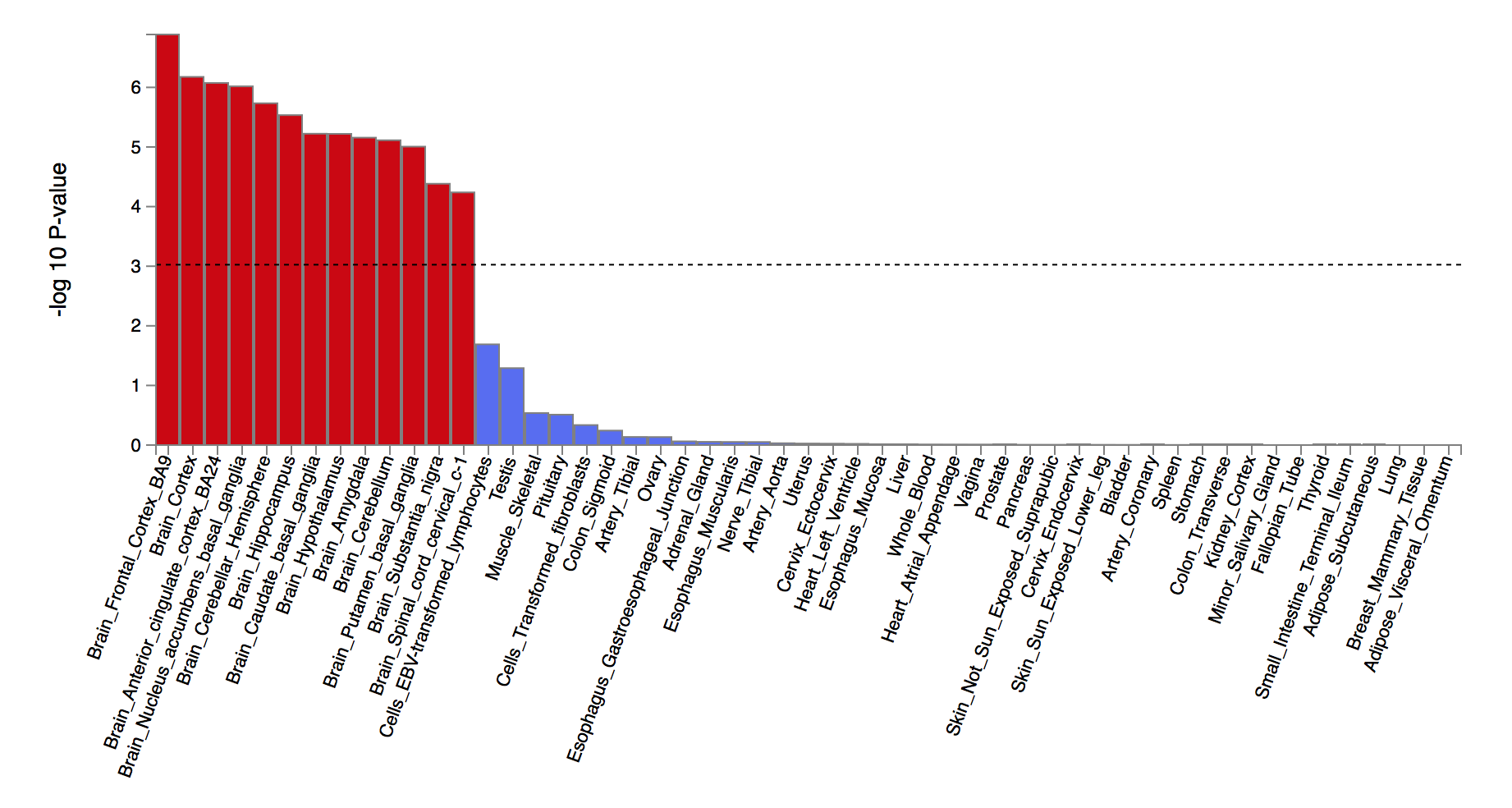


**Supplementary Figure 8:** General and specific tissue expression (DC2) – Graph is output from FUMA


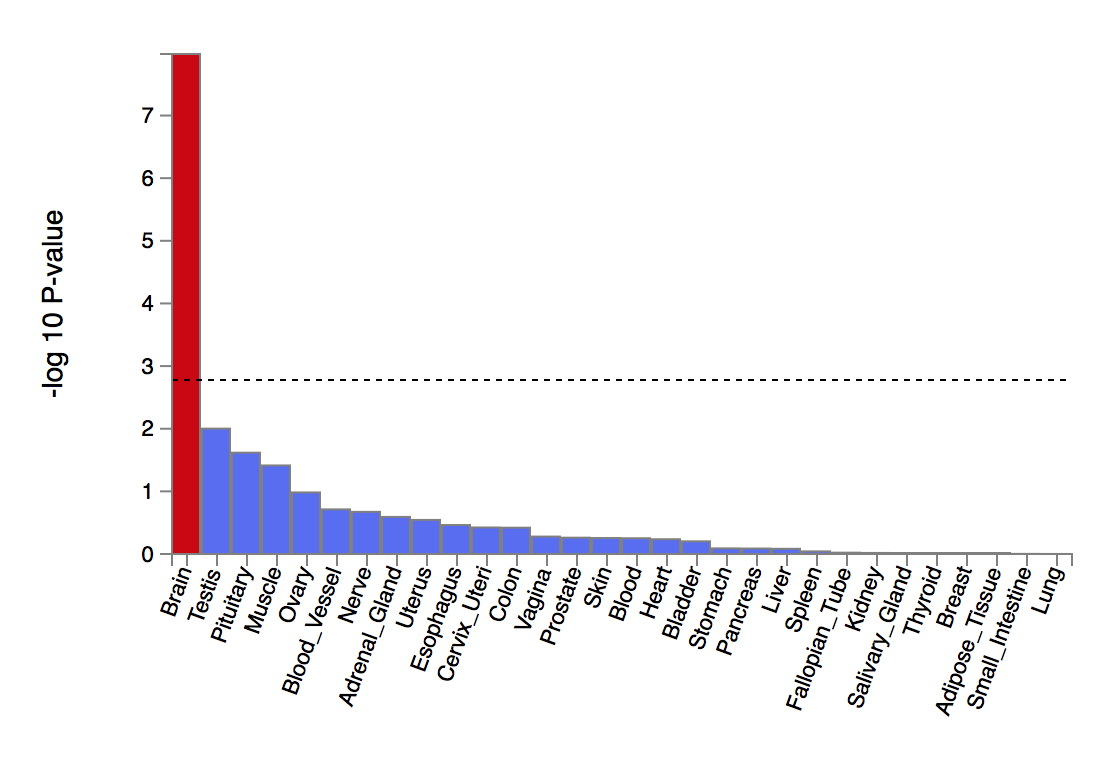


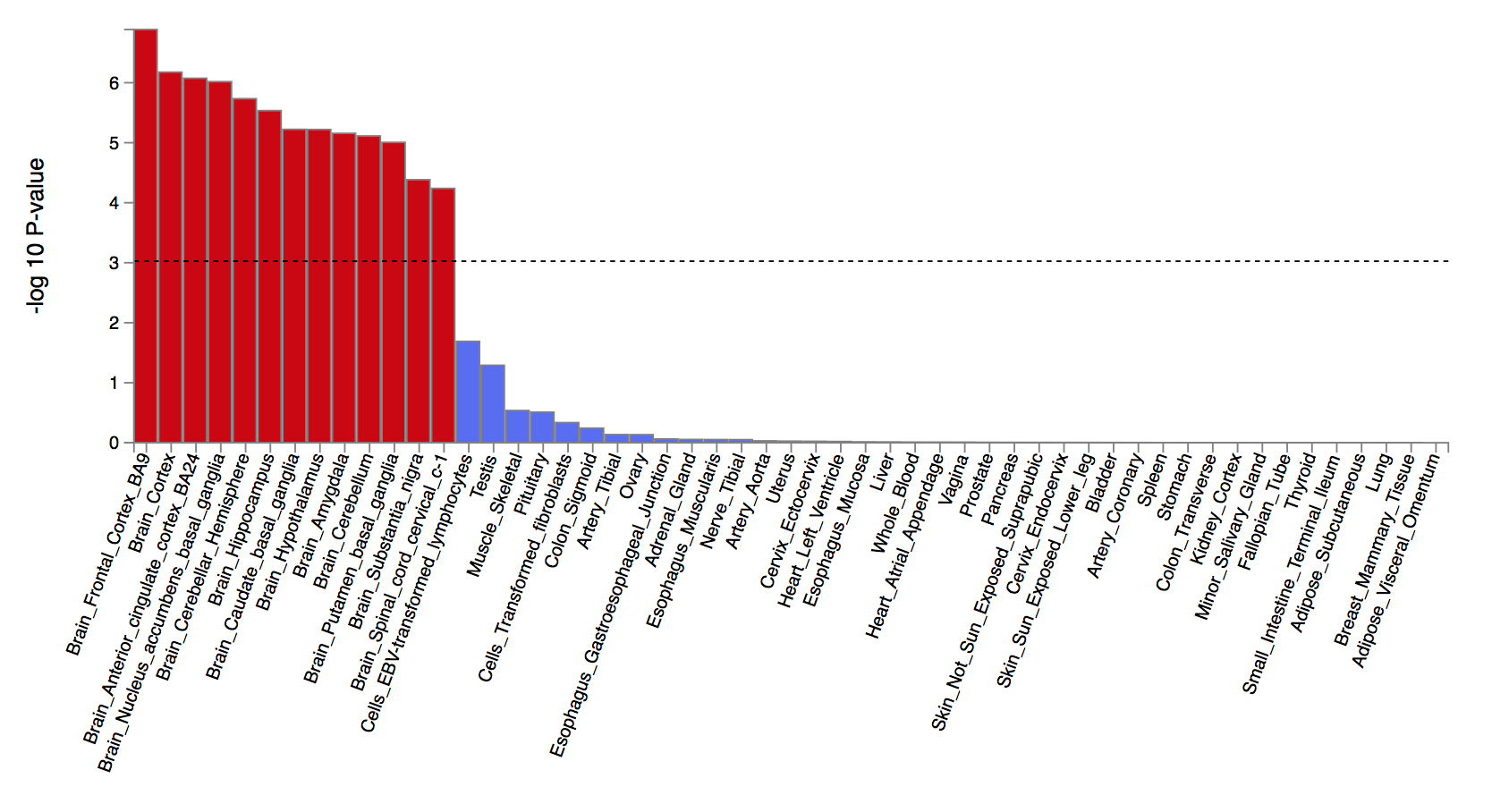


**Supplementary Figure 9.** GSMR results for BMI and Waist Hip Ratio


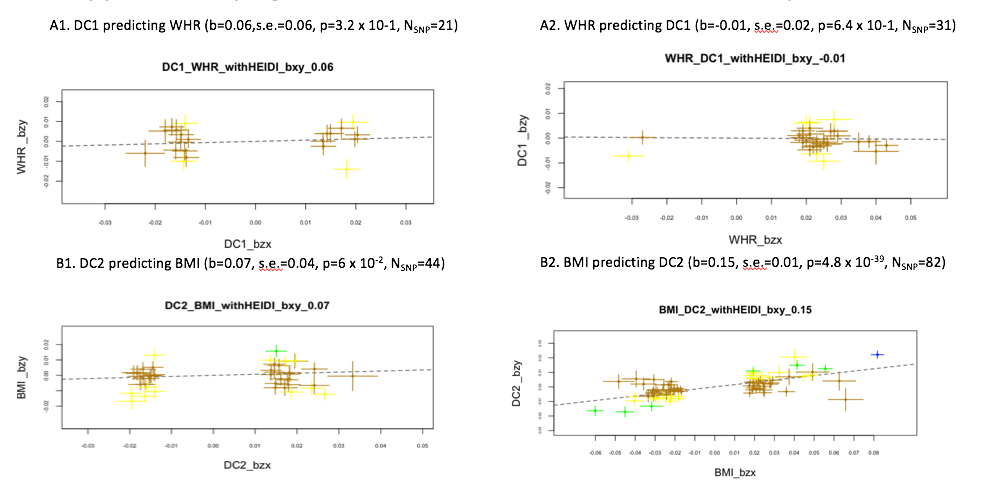


Notes: SNPs with 0.001<p<0.05 are in yellow, SNPs with 5e-08<p<0.0001 are in green and a SNPs with p<5e-08 are in blue. bxy= effects of risk factor (x) to outcome (y), bzy= effects of the instruments on outcome, bzx=effects of the instruments on risk factor, N_SNP_=number of SNPs.
