# Supplementary material for: Genome-wide association study of dietary intake in the UK biobank study and its associations with schizophrenia and other traits
Source: Transl Psychiatry. 2020 Feb 3;10:51. doi: 10.1038/s41398-020-0688-y (PMC7026164; doi:10.1038/s41398-020-0688-y)

Regional Associations Plots created using FUMA

Diet Component 1

rs10125463

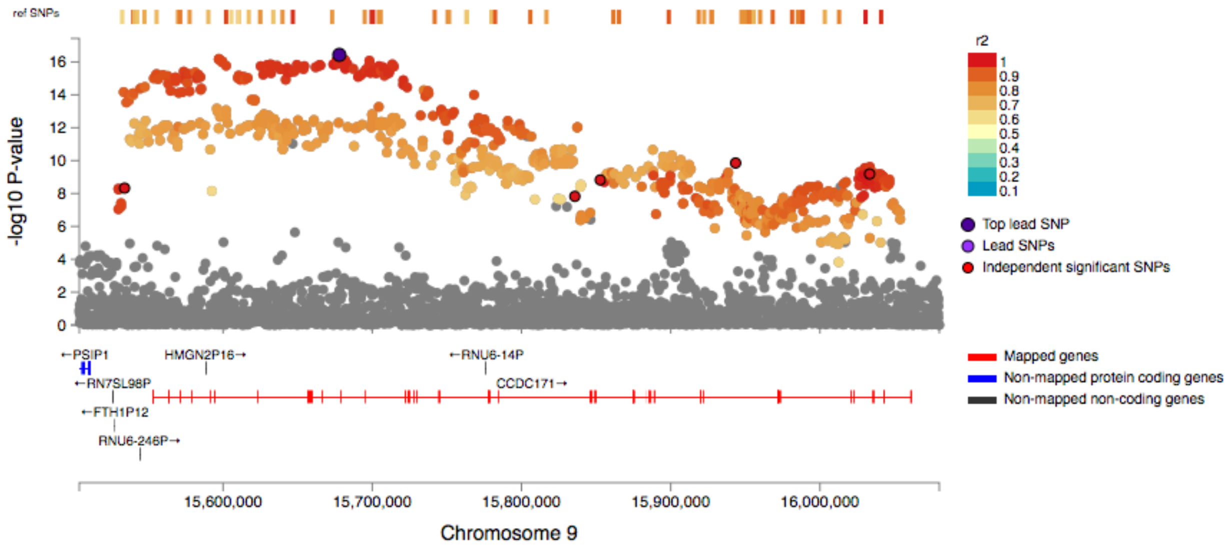

rs66495454

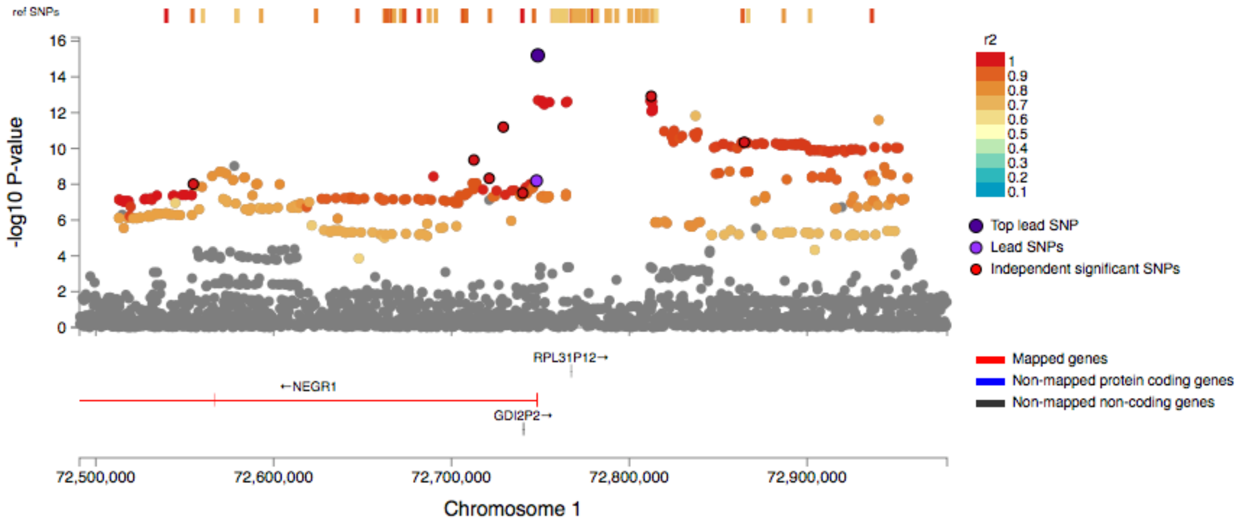

rs429358

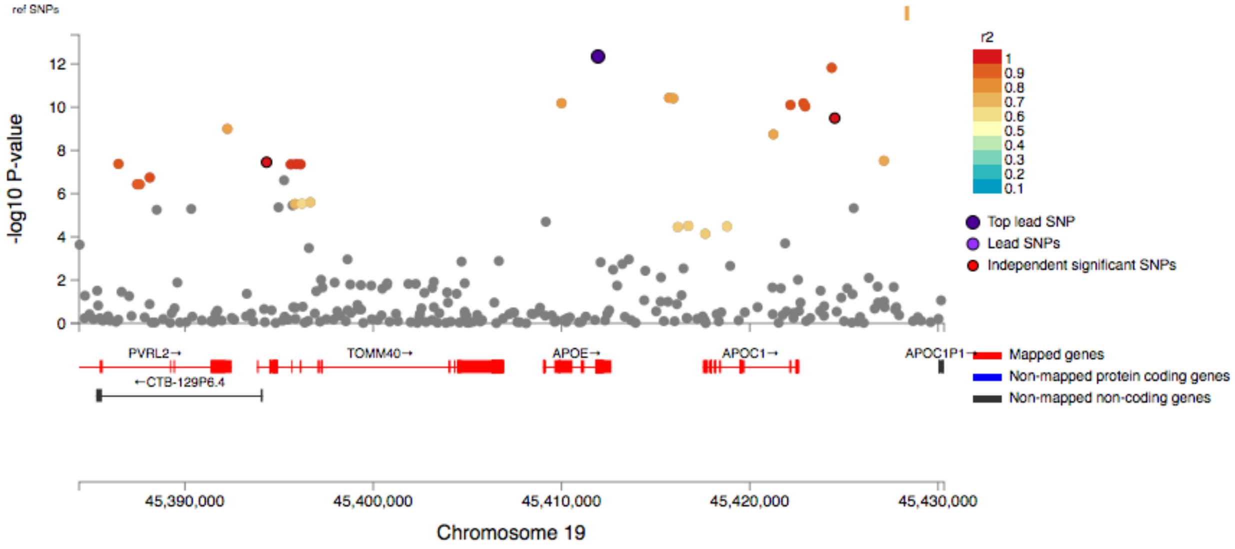

rs806794

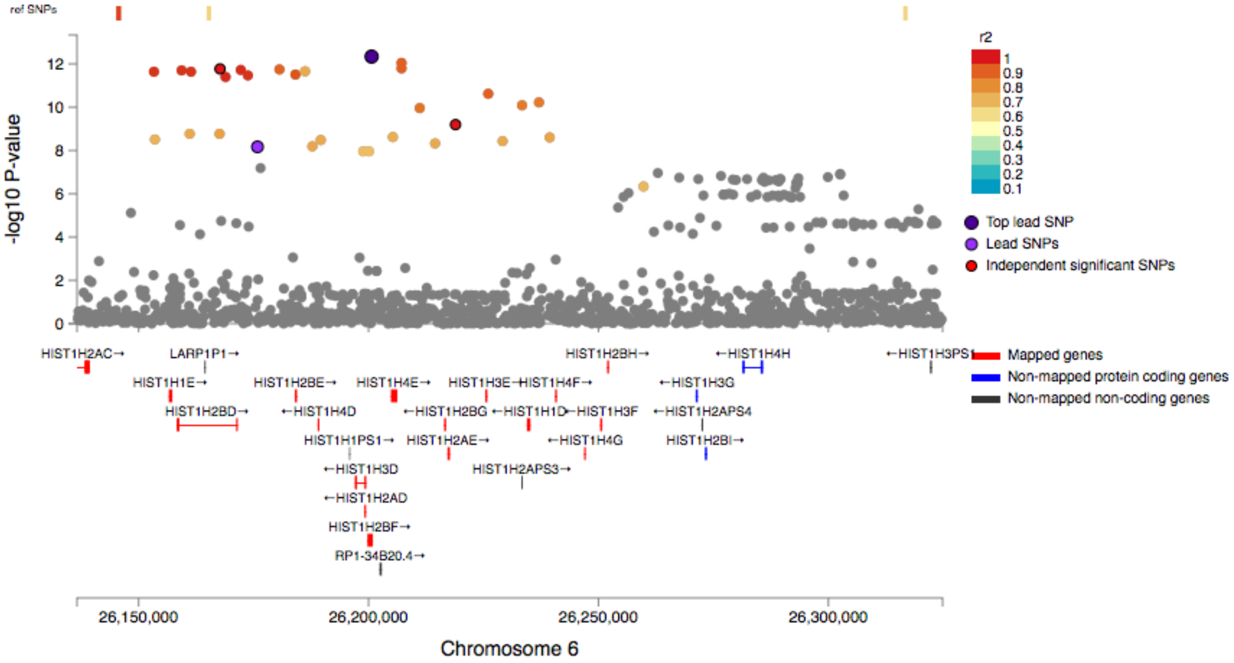

rs838144

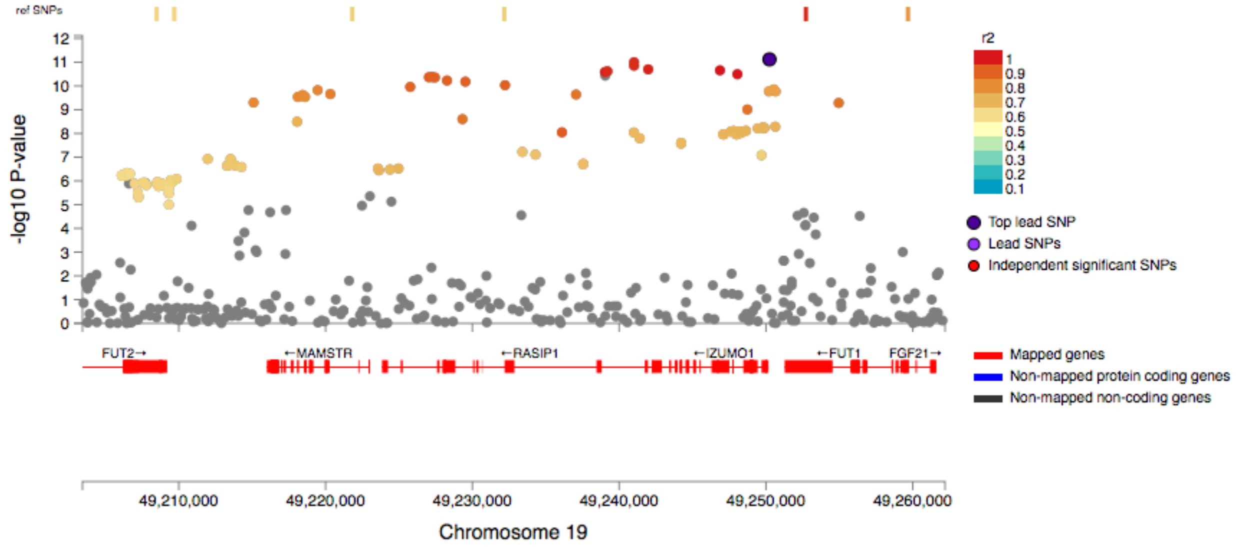

rs35797675

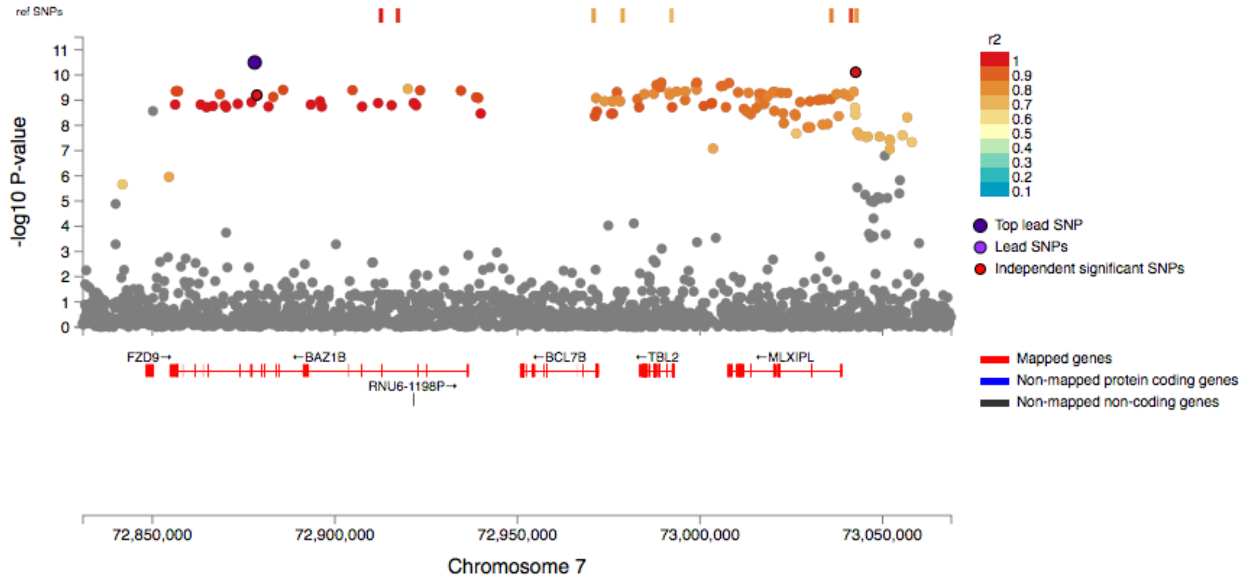

rs10064431

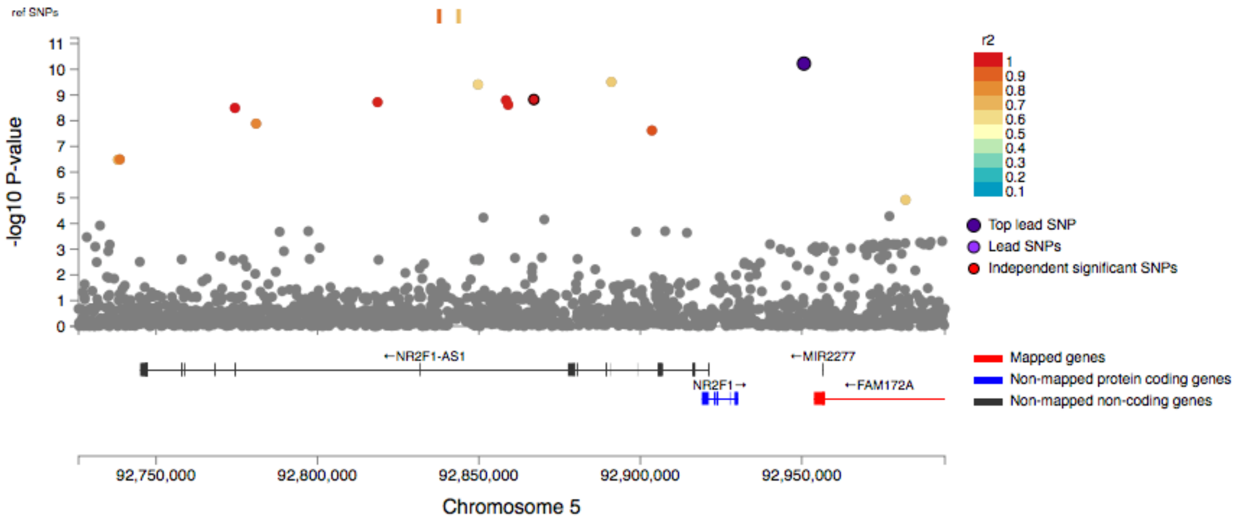

rs6478868

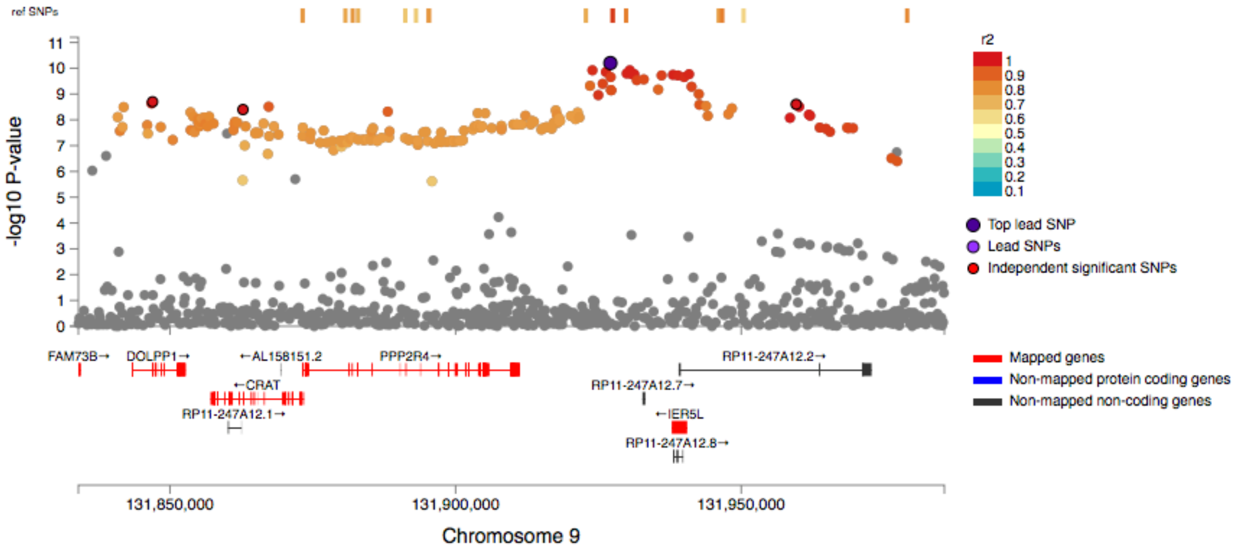

rs62106258

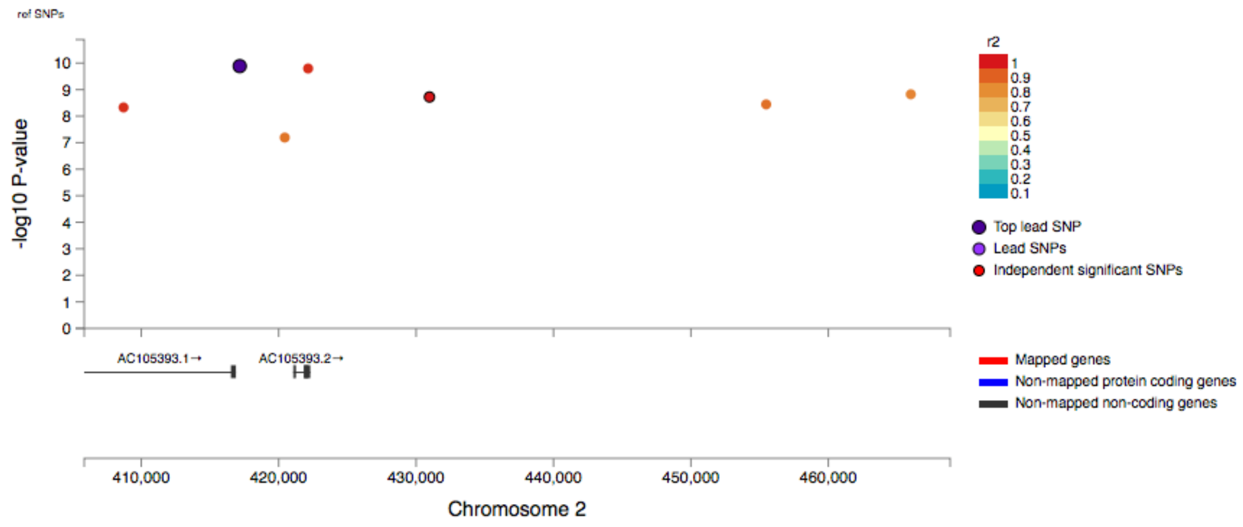

rs1912286

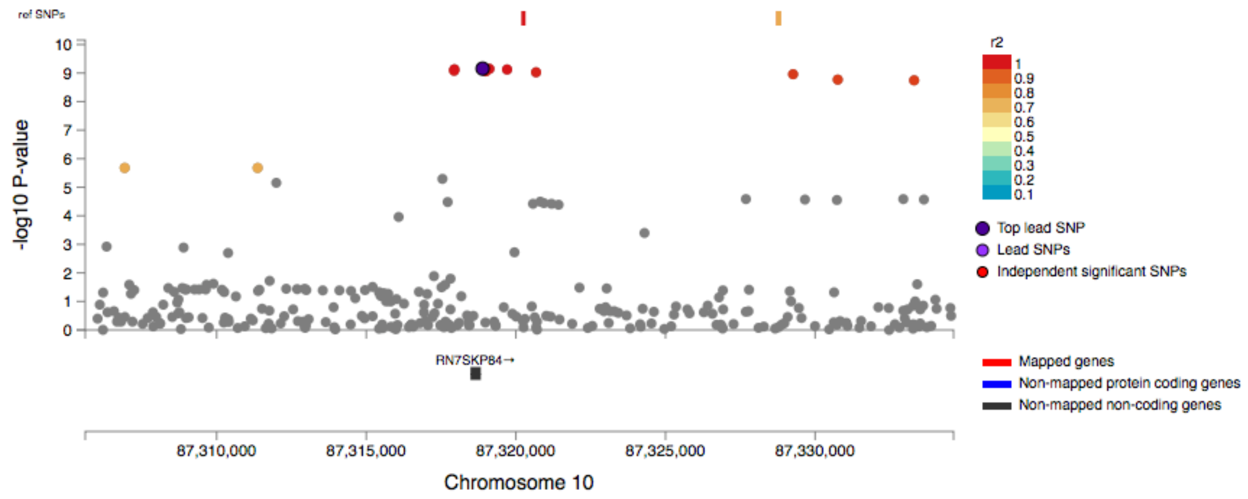

rs4759074

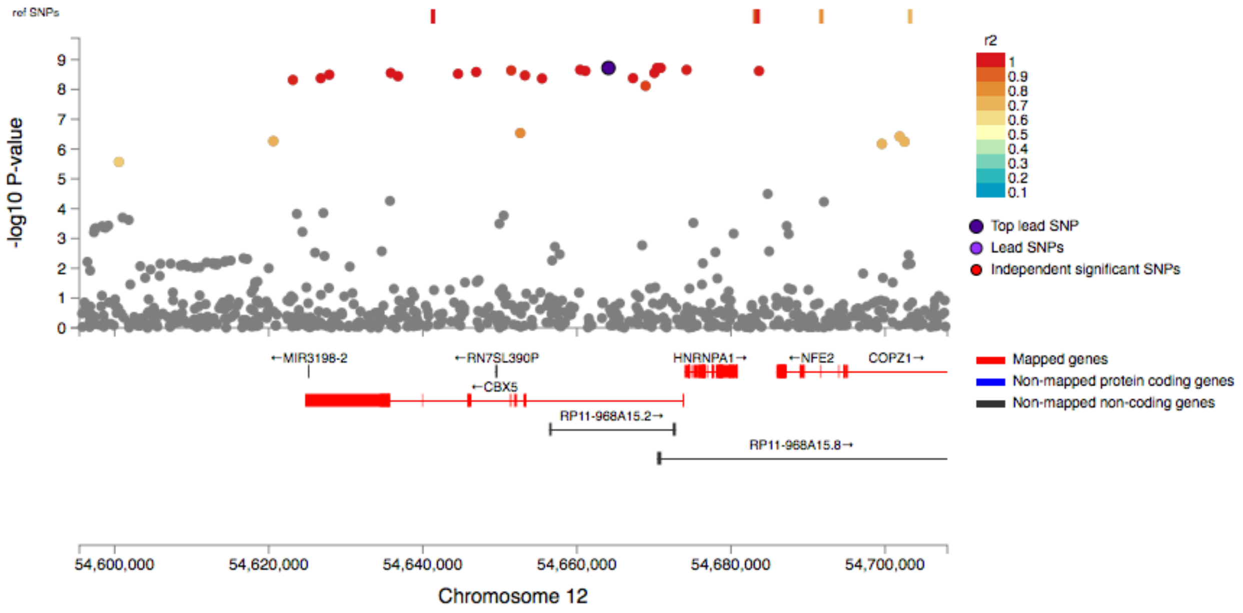

rs12232804

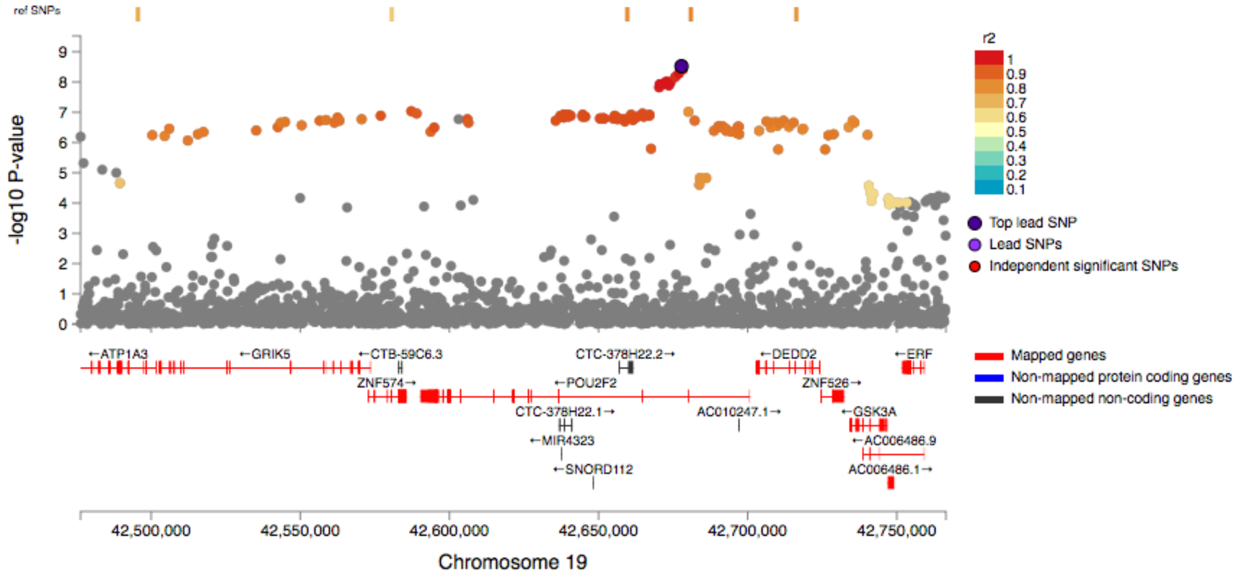

rs136528

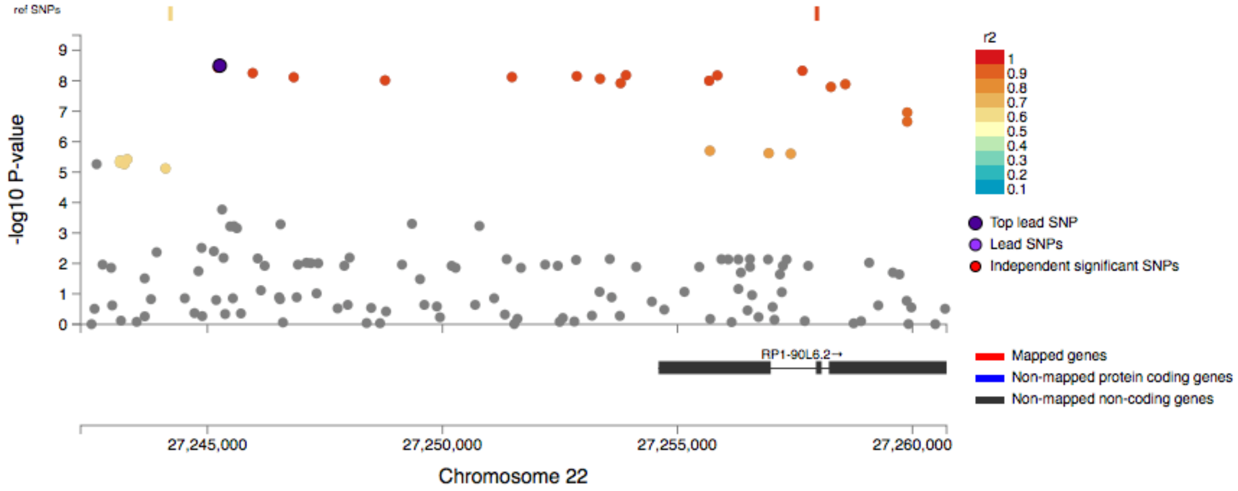

rs7644667

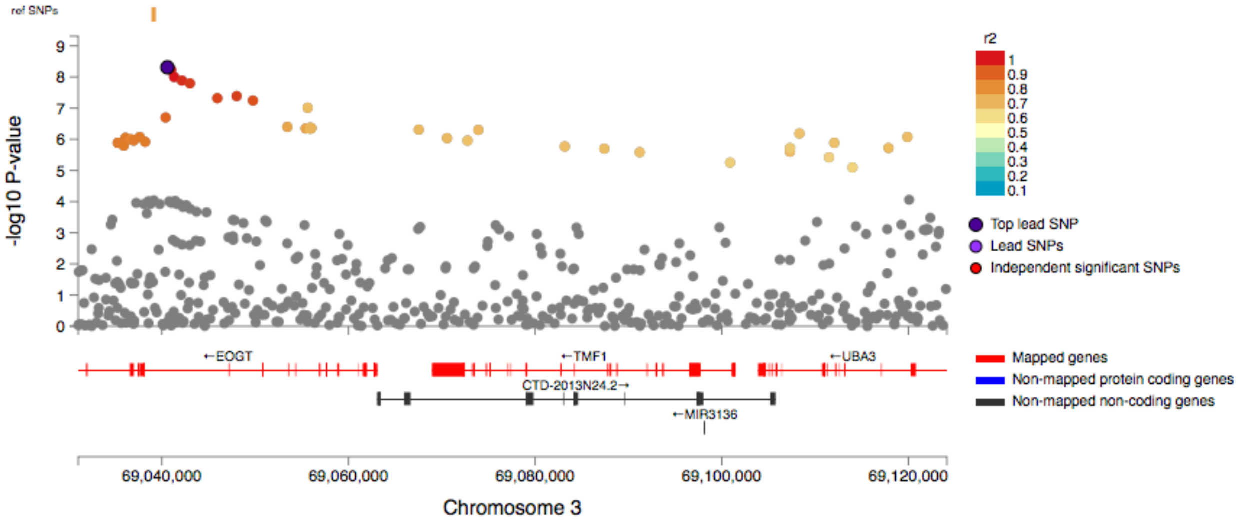

rs3101341

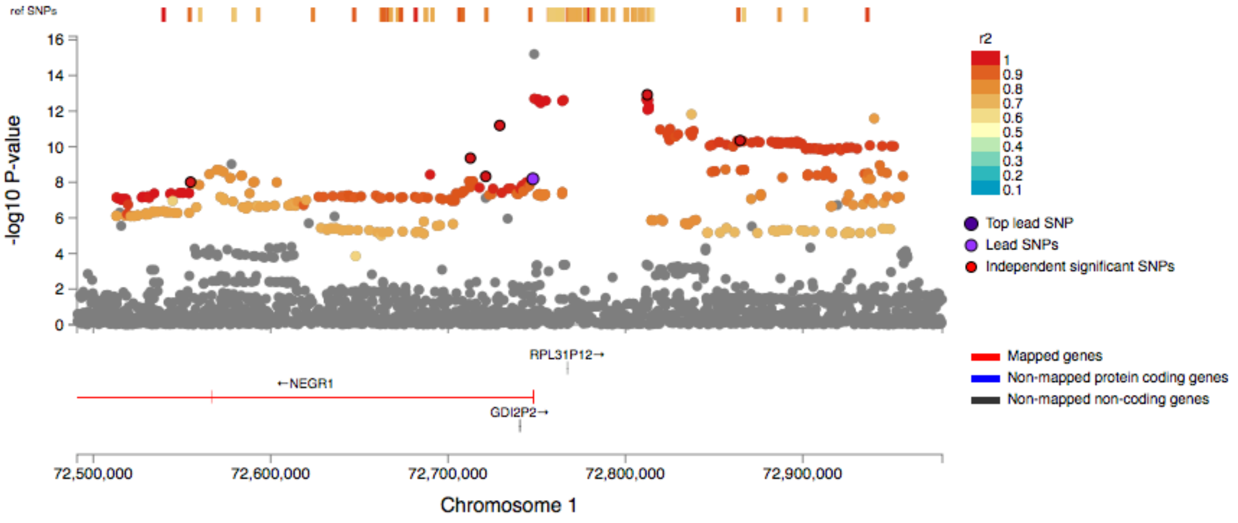

rs9379831

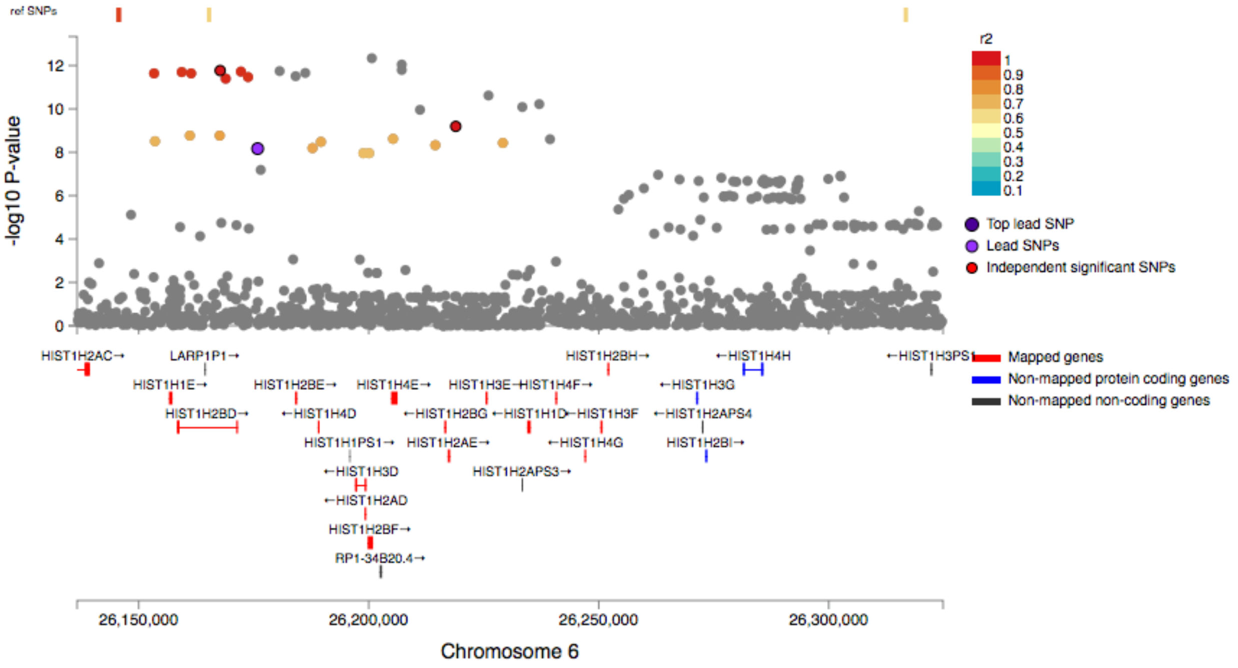

rs6786550

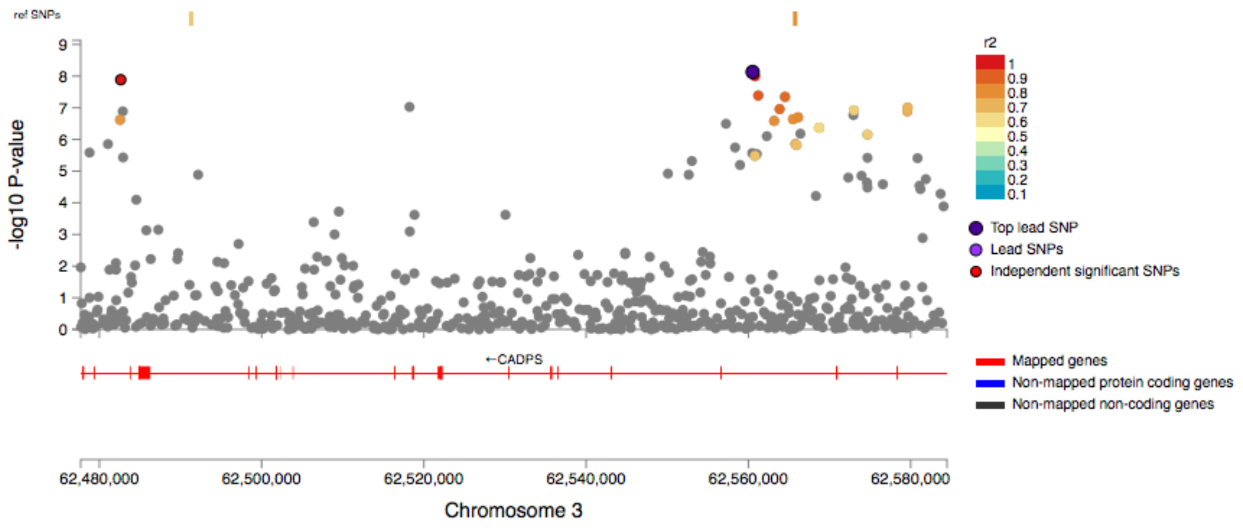

rs79564737

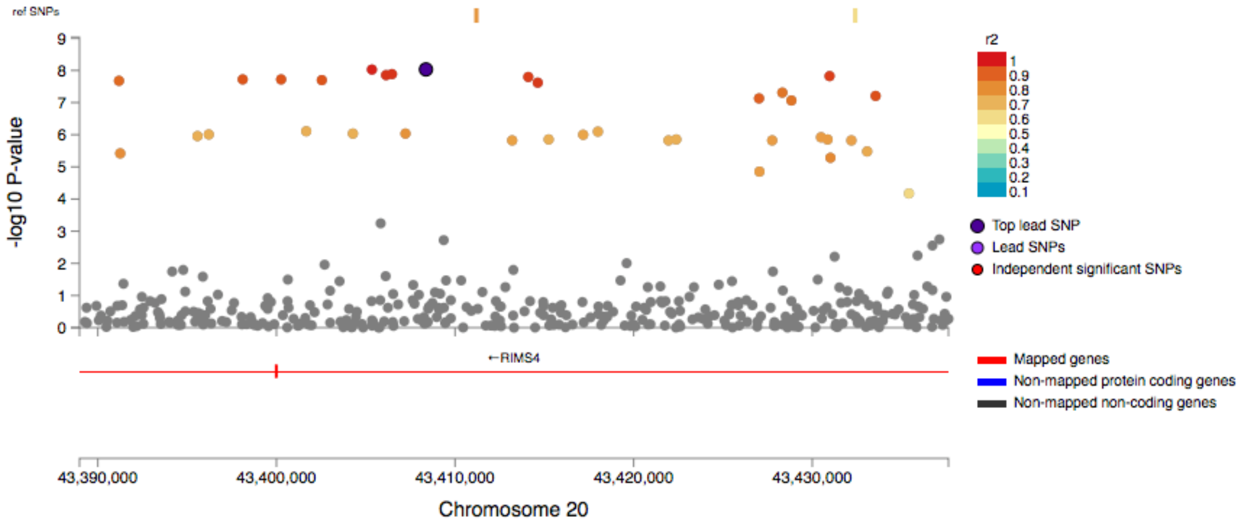

rs10900457

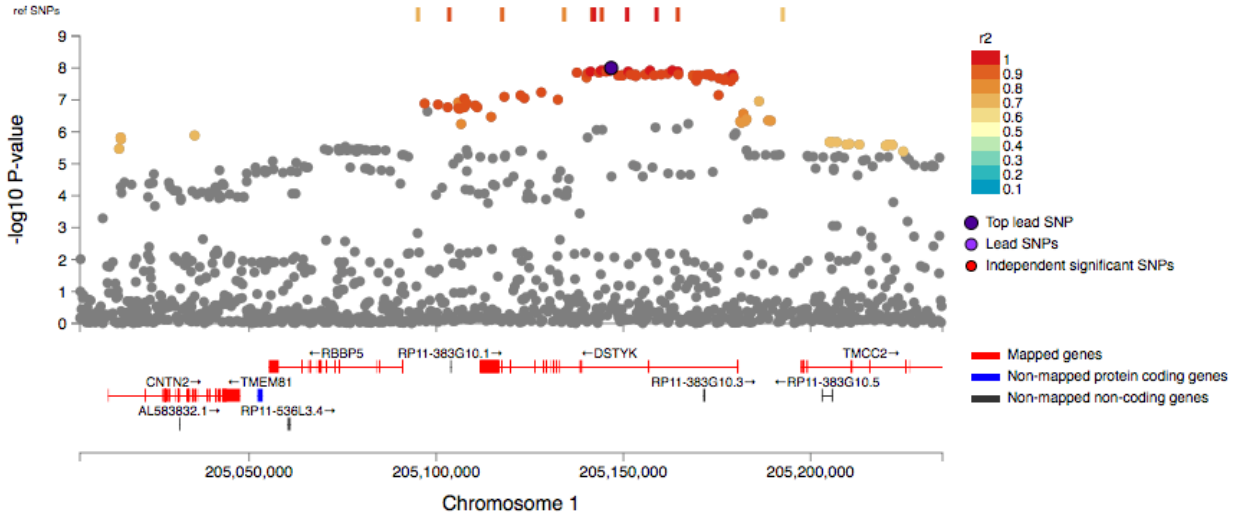

rs139911

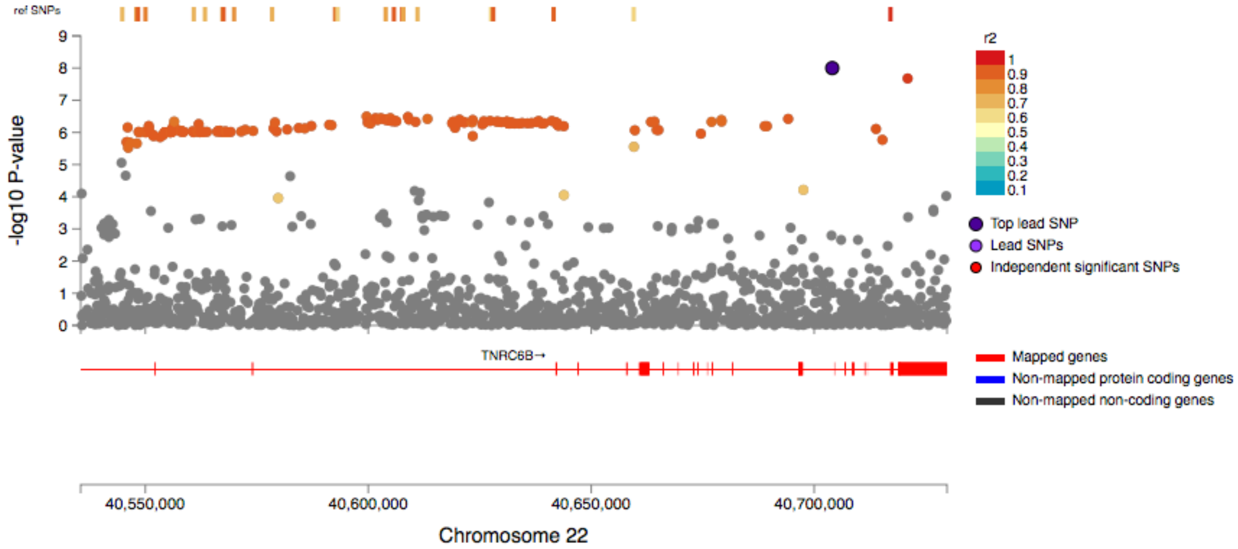

rs13340130

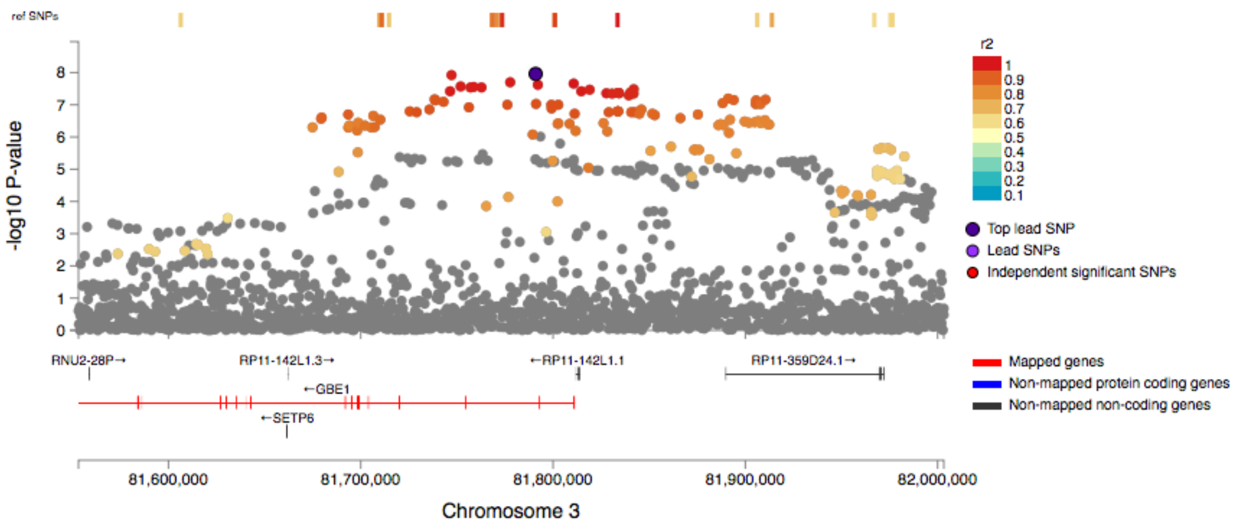

rs12103229

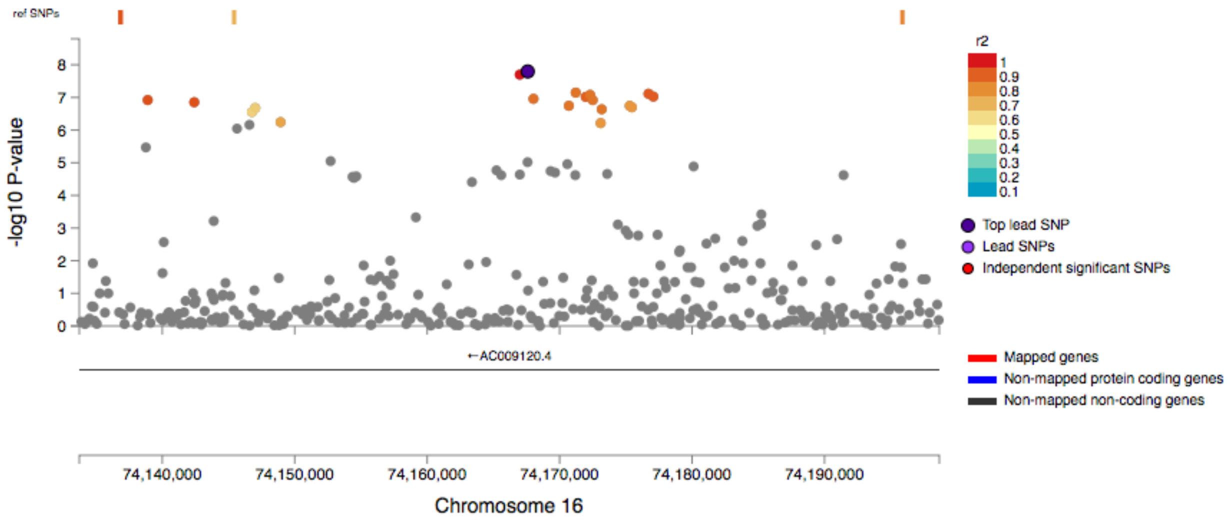

rs3909727

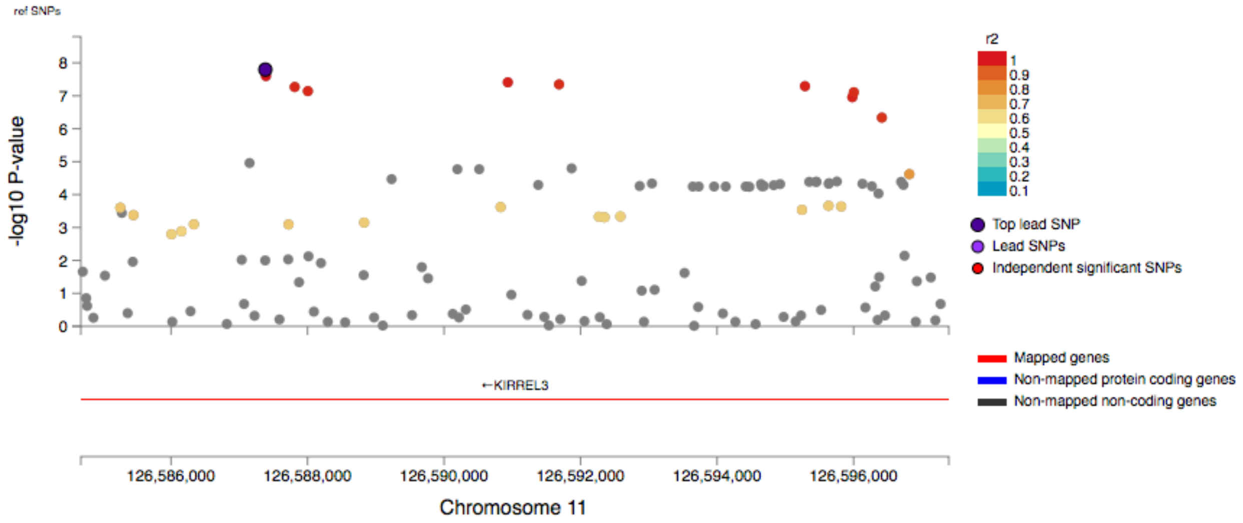

rs4034907

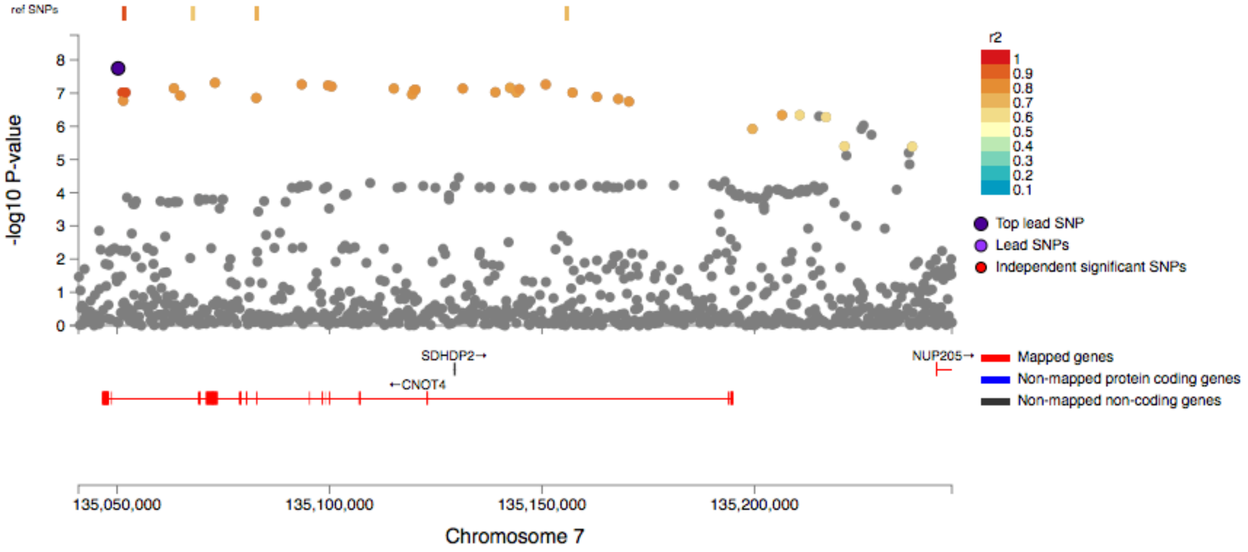

rs36016753

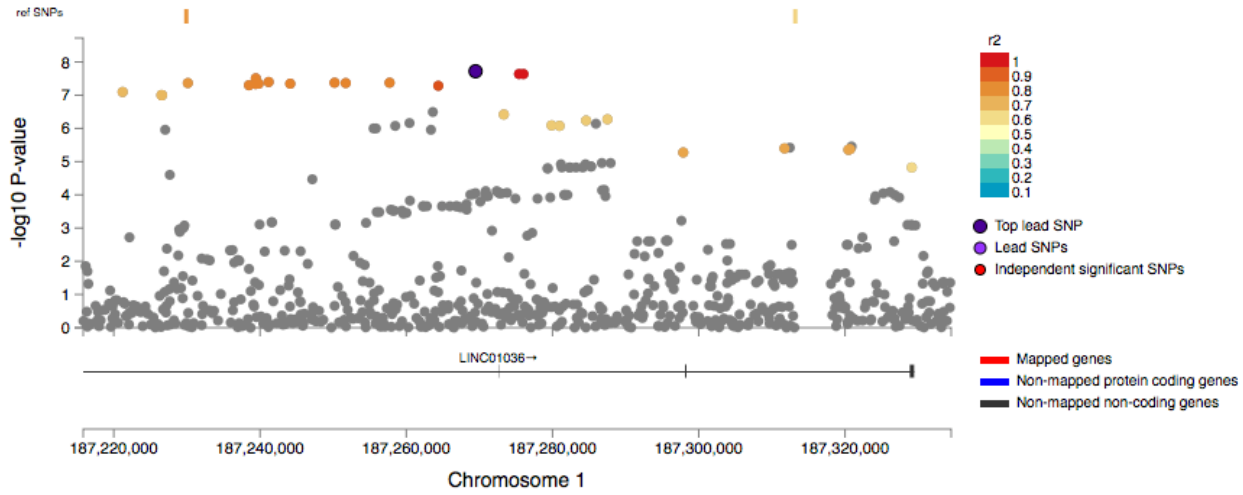

rs701760

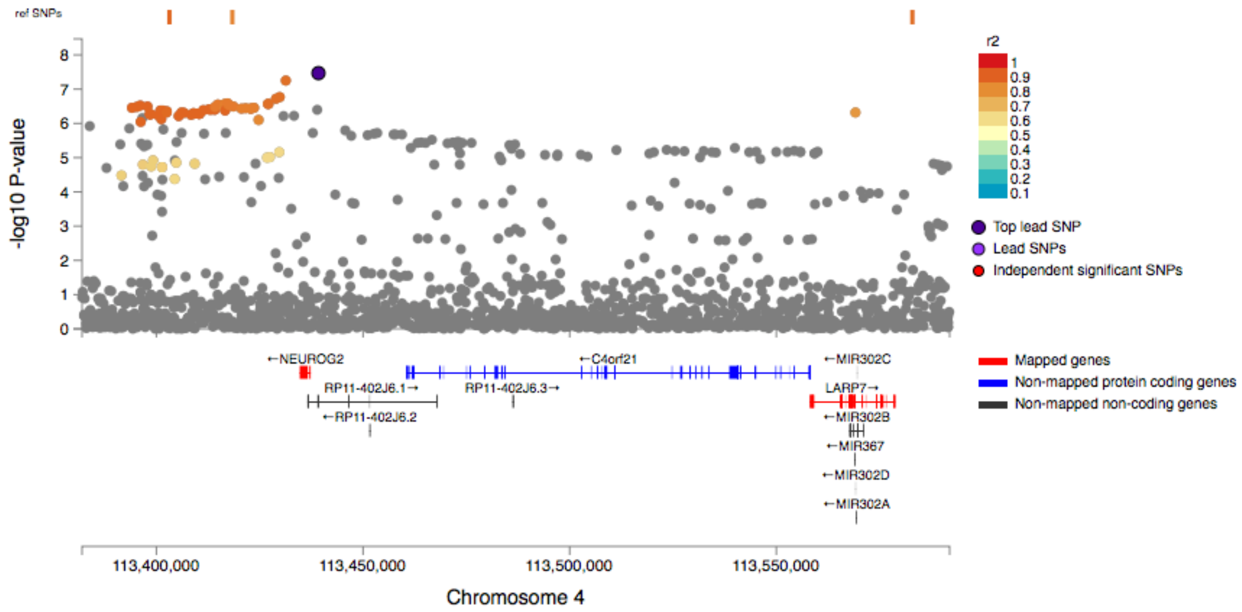

rs202637

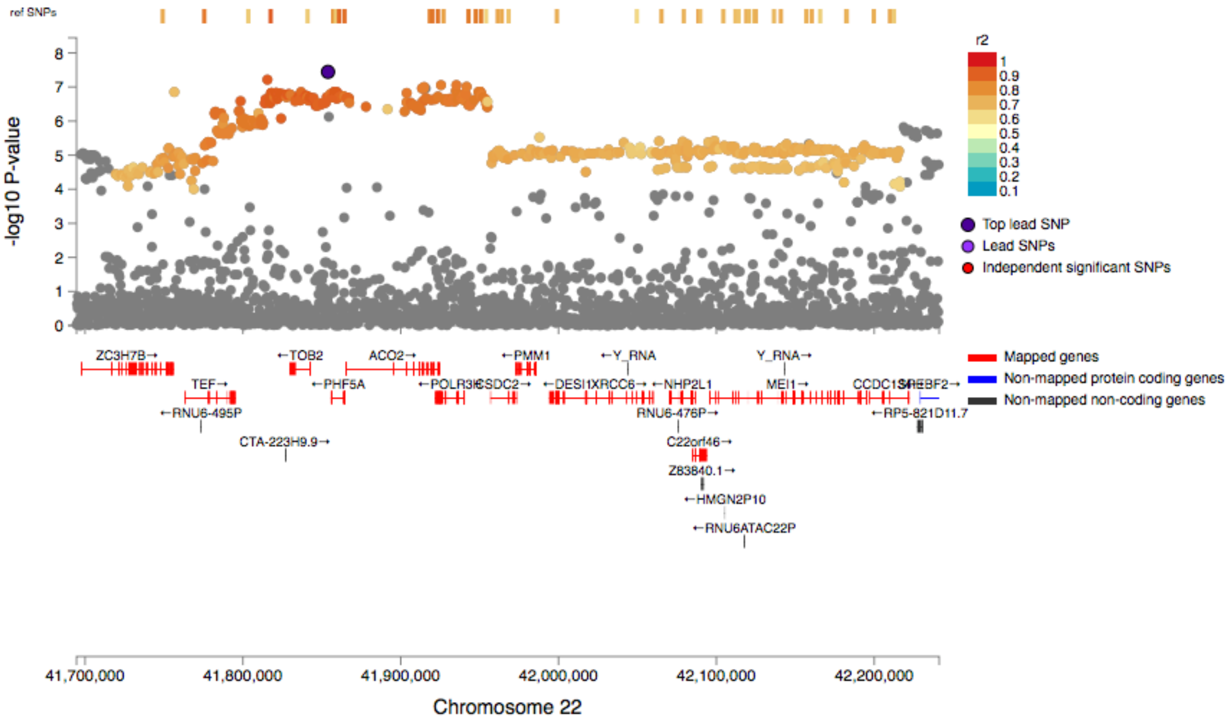

rs506589

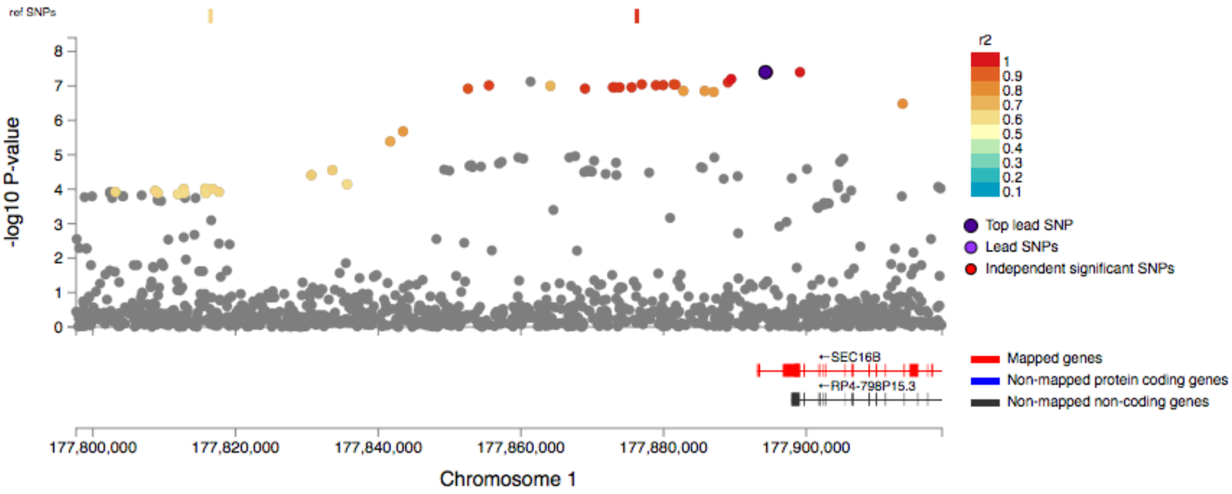

rs300046

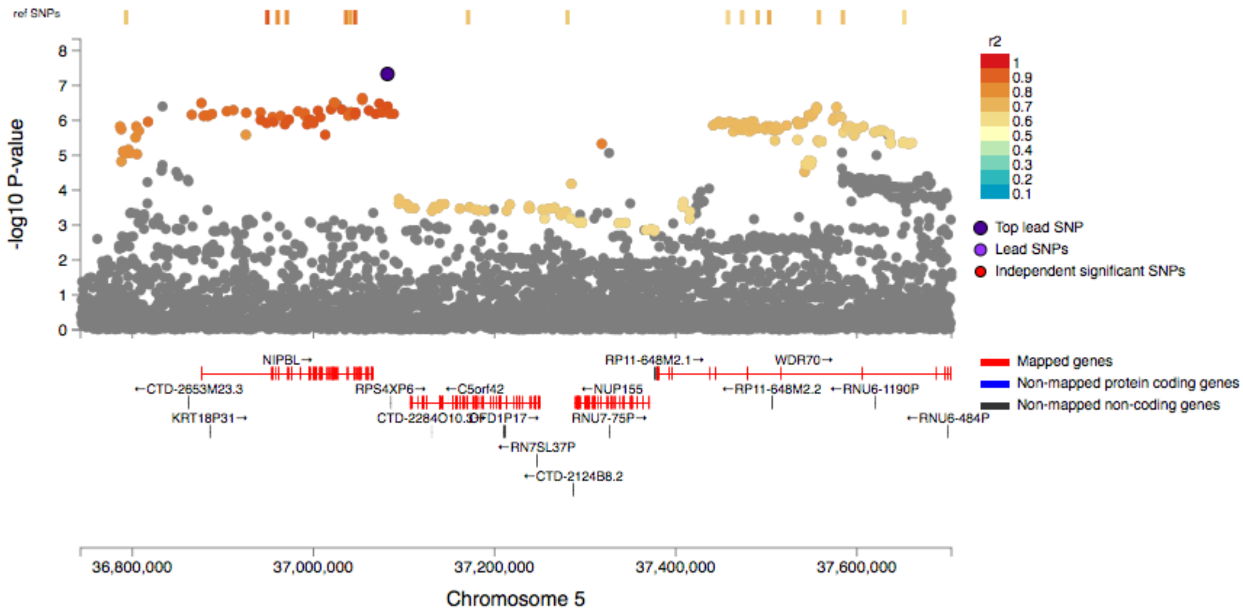

Diet Component 2

rs946711

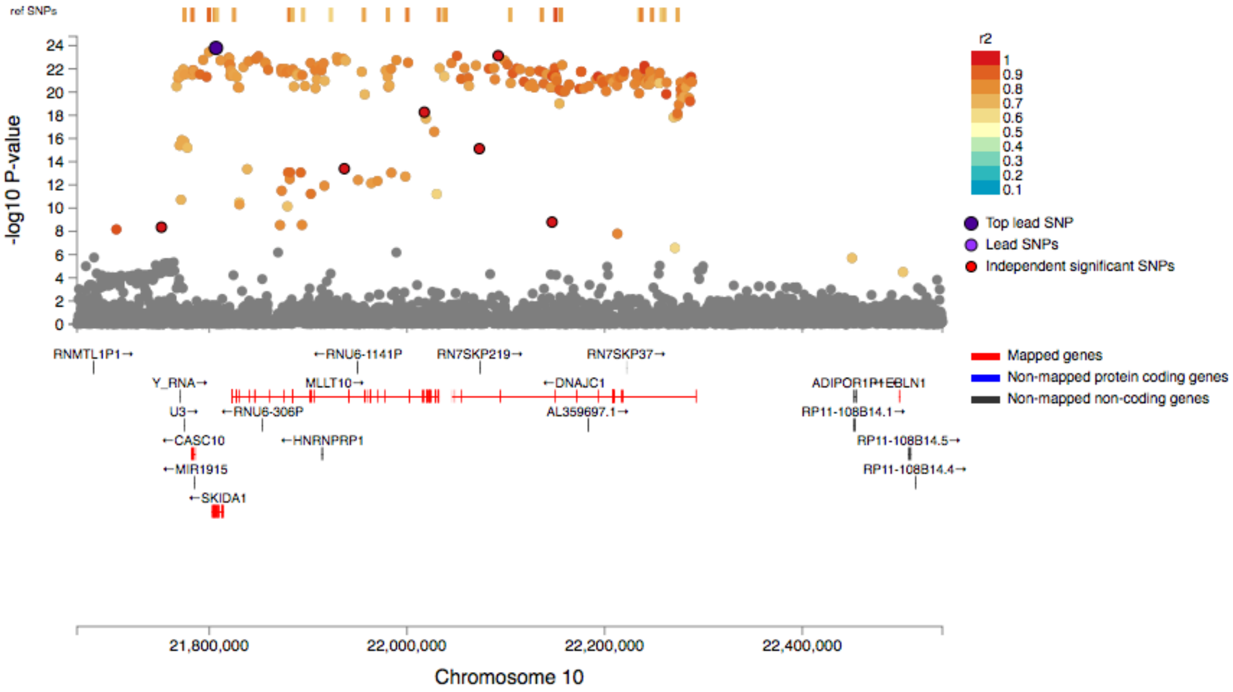

rs56094641

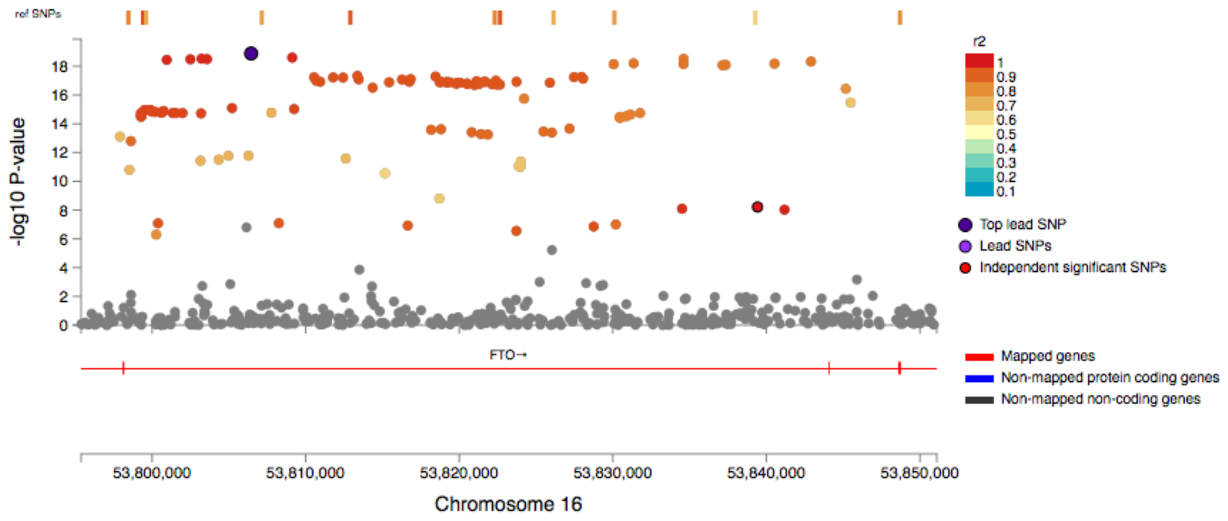

rs35287743

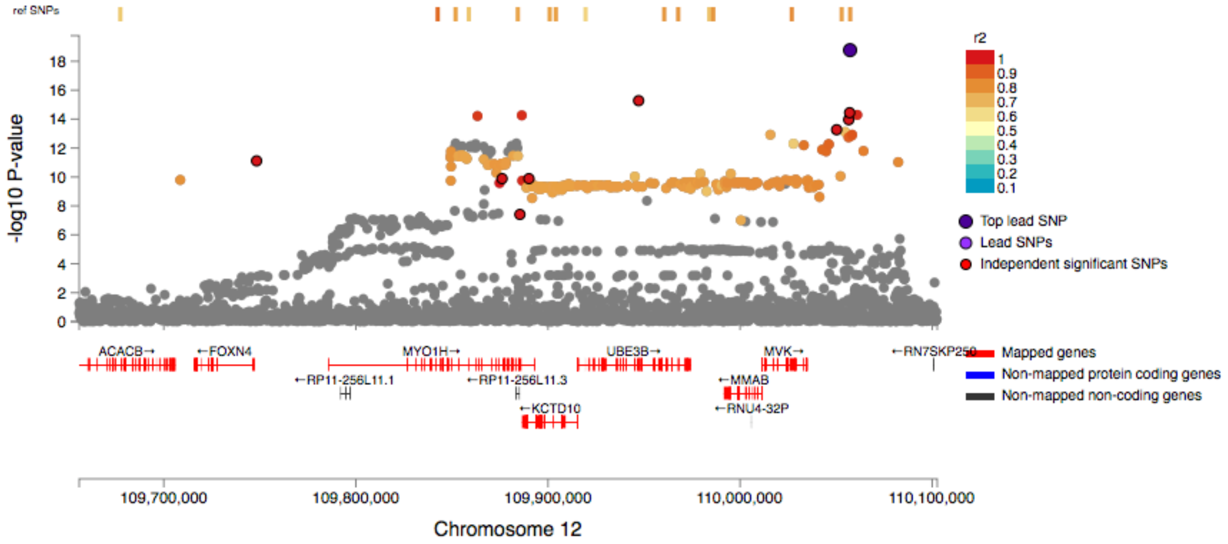

rs1370063

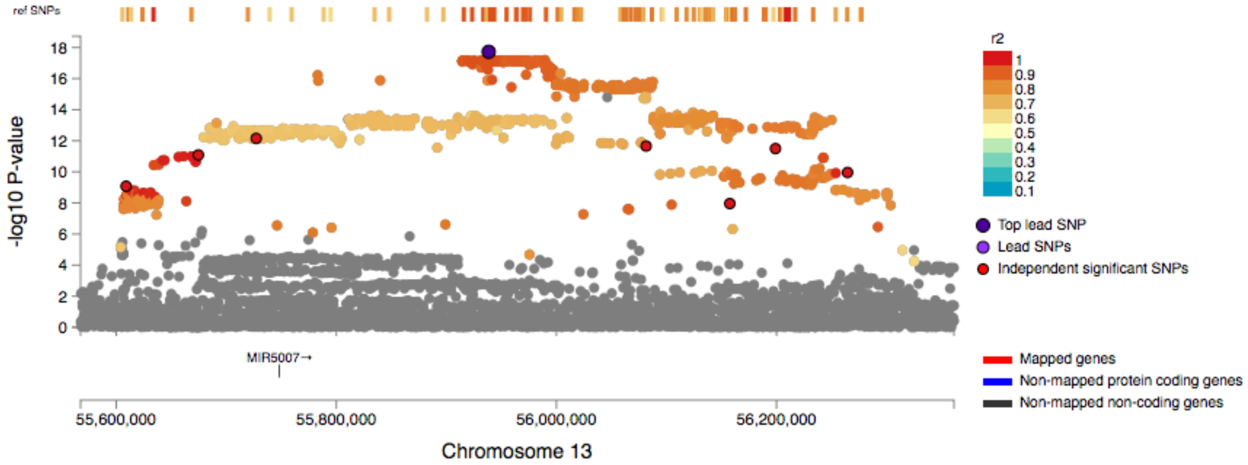
ref SNPs
-log10 P-value
r2
Top lead SNP
Lead SNPs
Independent significant SNPs
MIR5007→
Mapped genes
Non-mapped protein coding genes
Non-mapped non-coding genes
55,600,000
55,800,000
56,000,000
56,200,000
Chromosome 13

rs36095784

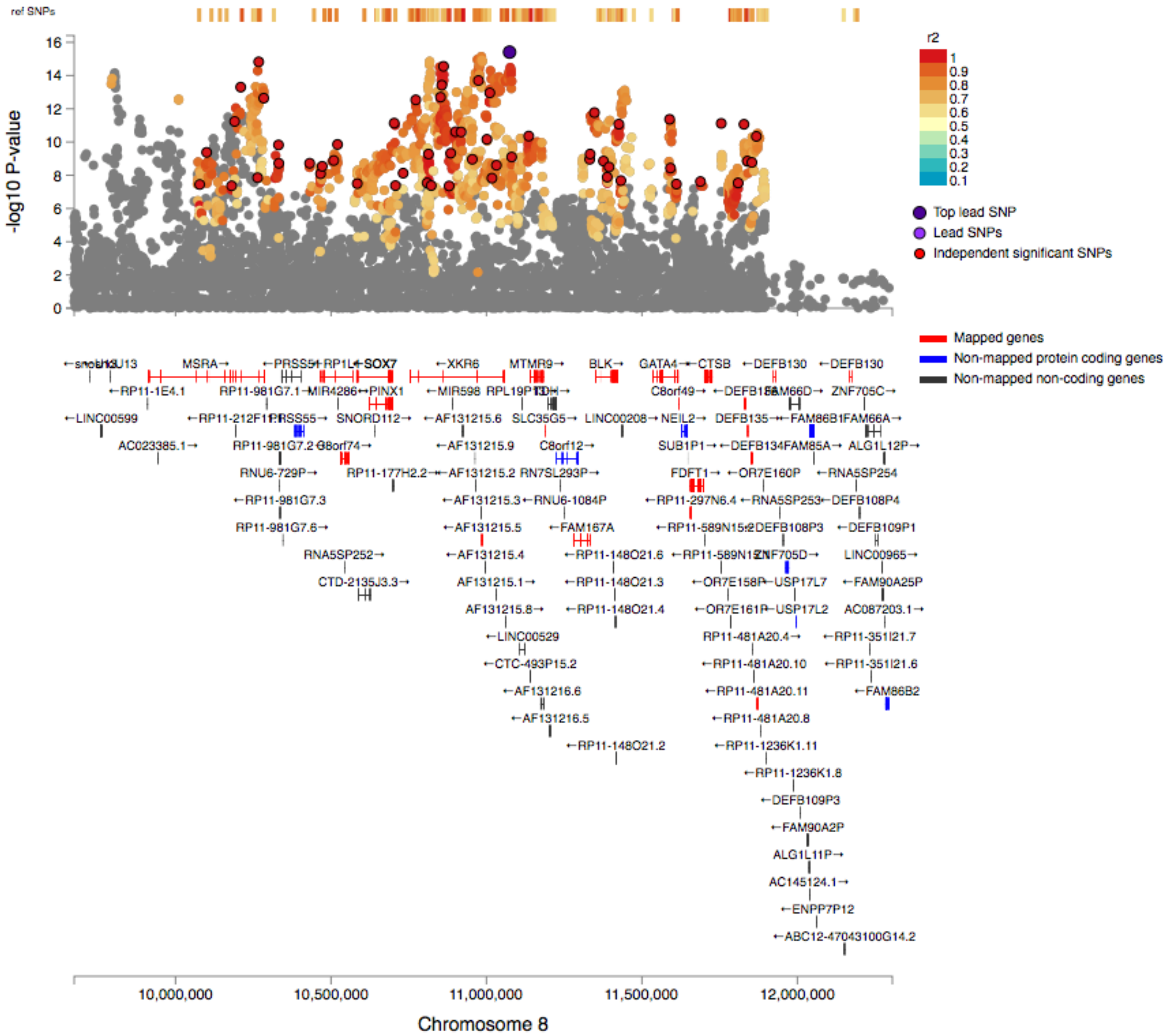
ref SNPs
-log10 P-value
r2
Top lead SNP
Lead SNPs
Independent significant SNPs
Mapped genes
Non-mapped protein coding genes
Non-mapped non-coding genes
MSRA→
←XKR6
MTMR9→
BLK→
GATA4→
←CTSB
←DEFB130
←RP11-1E4.1
←LINC00599
AC023385.1→
←MIR598
SNORD112→
SLC35G5→
LINC00208→
NEIL2→
C8orf12→
SUB1P1→
FDFT1→
←FAM167A
ALG1L12P→
←RNA5SP254
←DEFB108P4
←DEFB109P1
LINC00965→
←FAM90A25P
←LINC00529
←CTC-493P15.2
←AF131216.6
←AF131216.5
←RP11-148O21.2
←FAM86B2
←DEFB109P3
←FAM90A2P
ALG1L11P→
AC145124.1→
←ENPP7P12
←ABC12-47043100G14.2
10,000,000
10,500,000
11,000,000
11,500,000
12,000,000
Chromosome 8

rs9650622

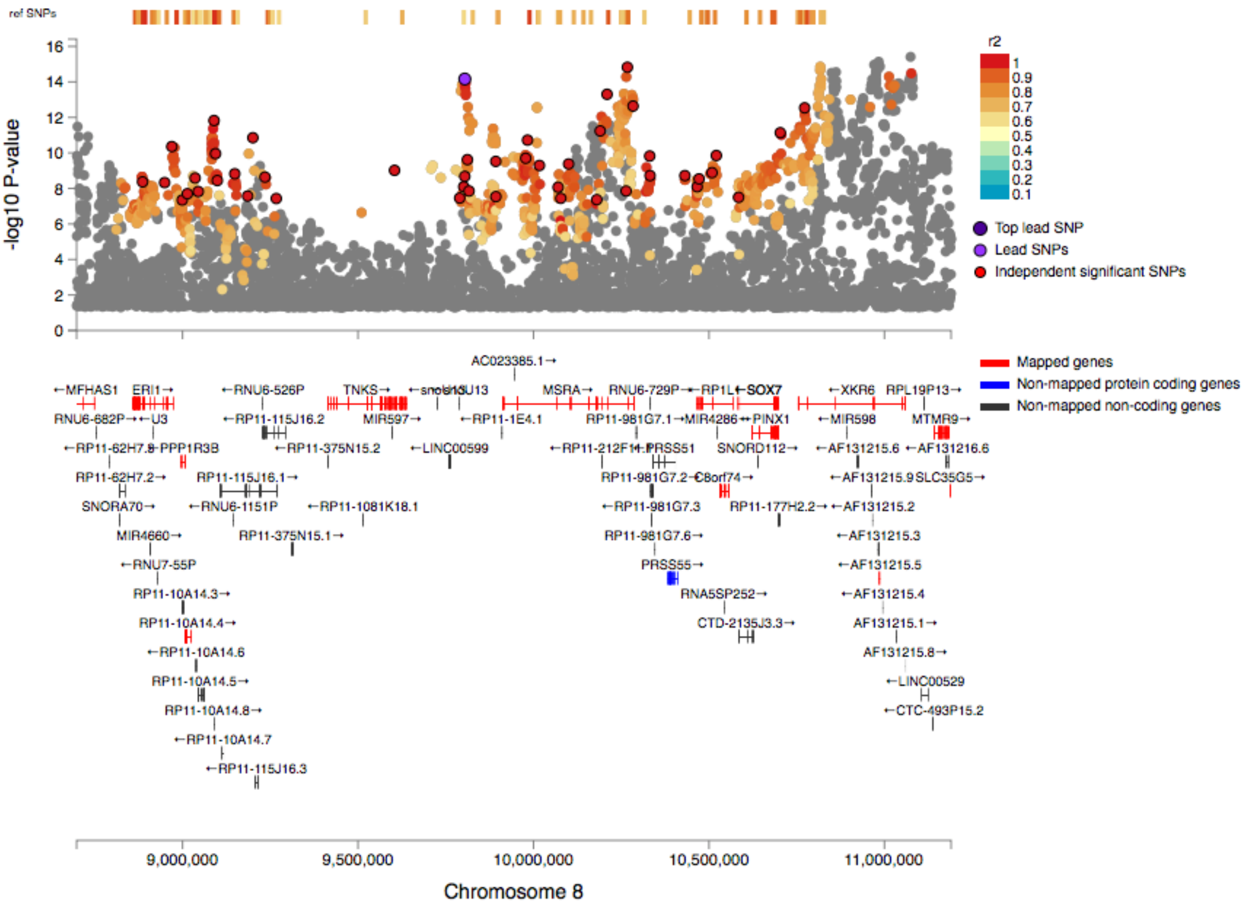

rs1248825

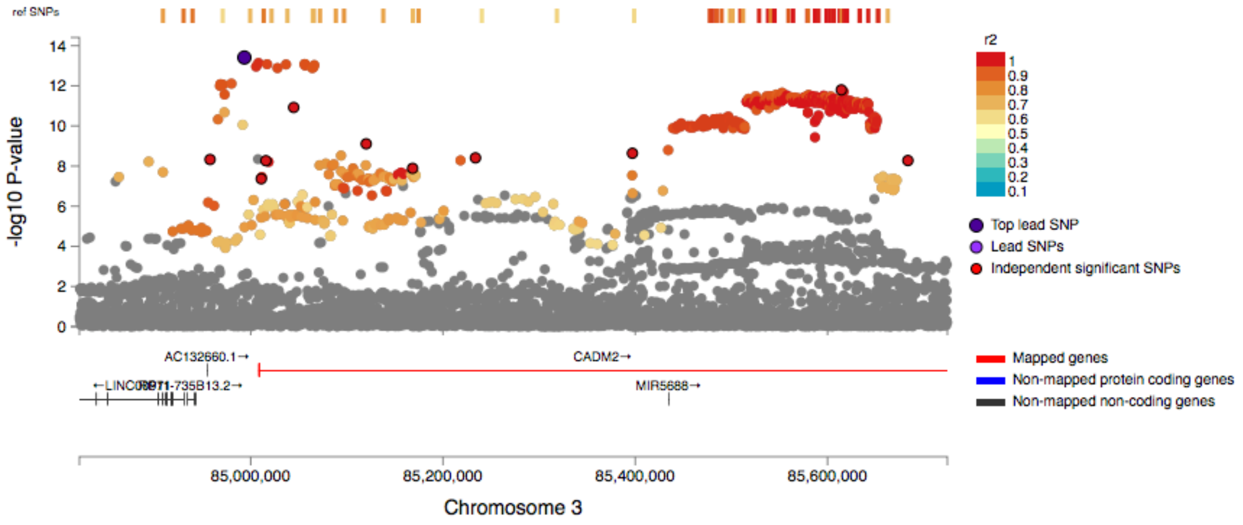

rs6690619

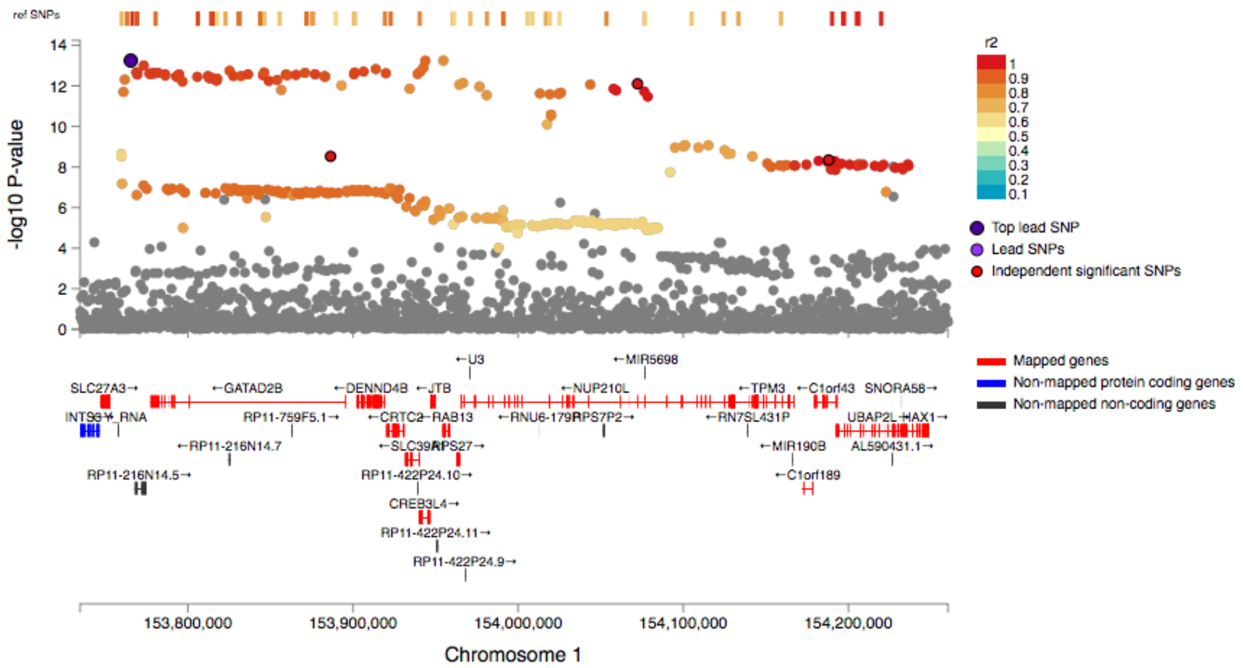

rs4953150

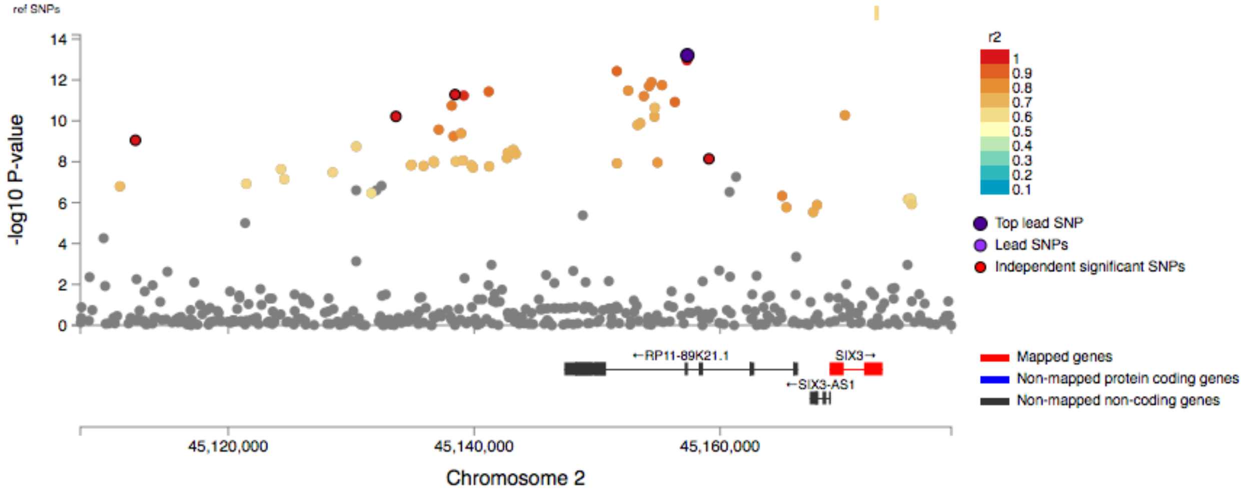

rs7969719

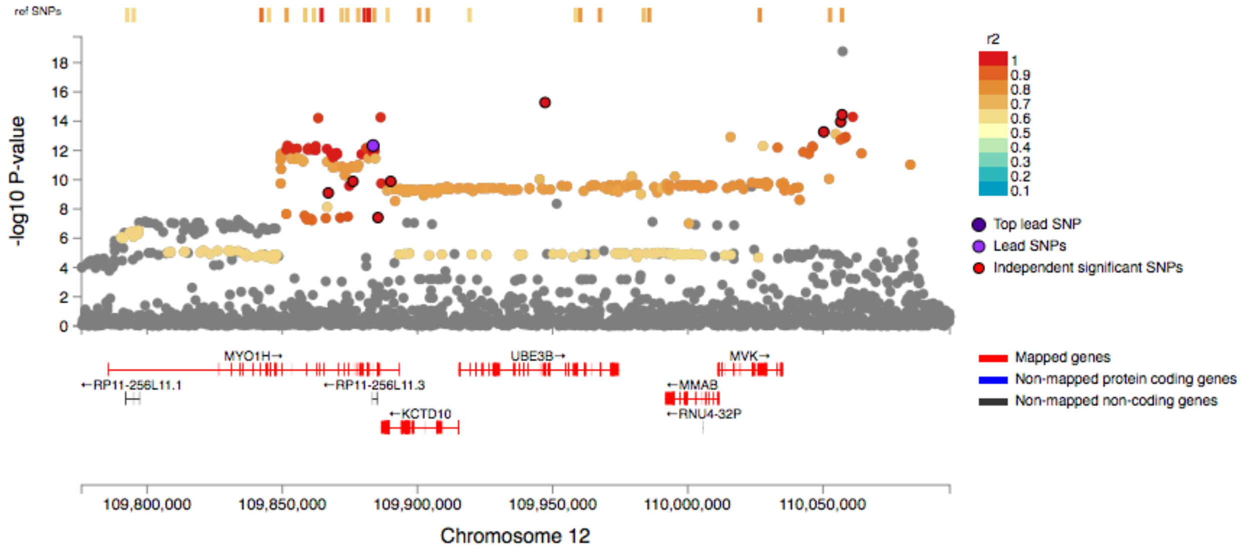

rs380743

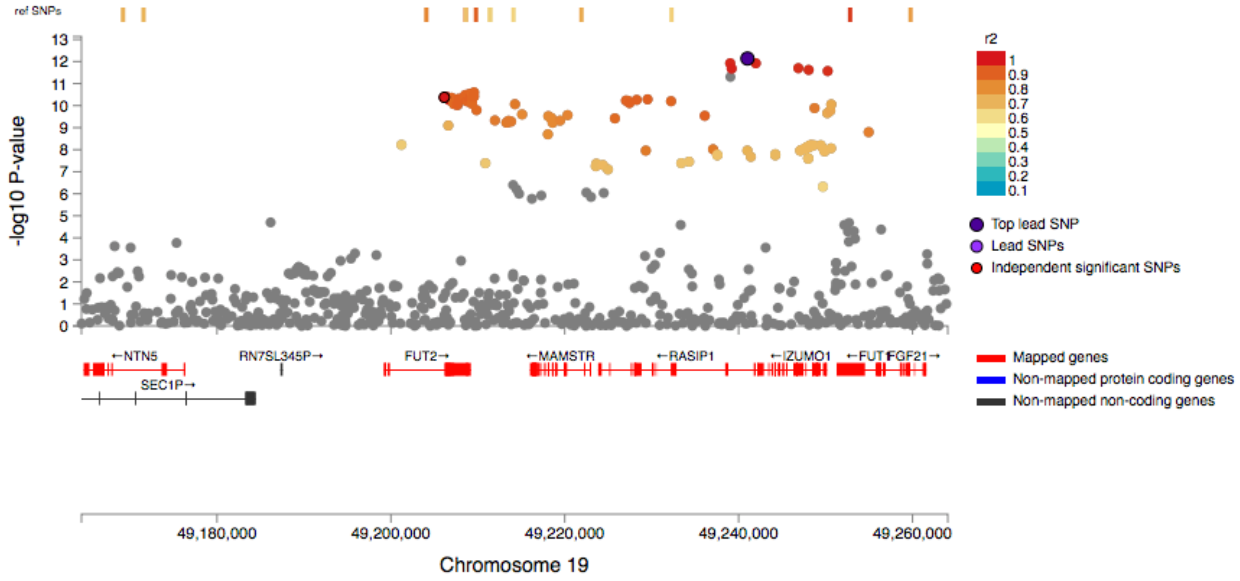

rs56367474

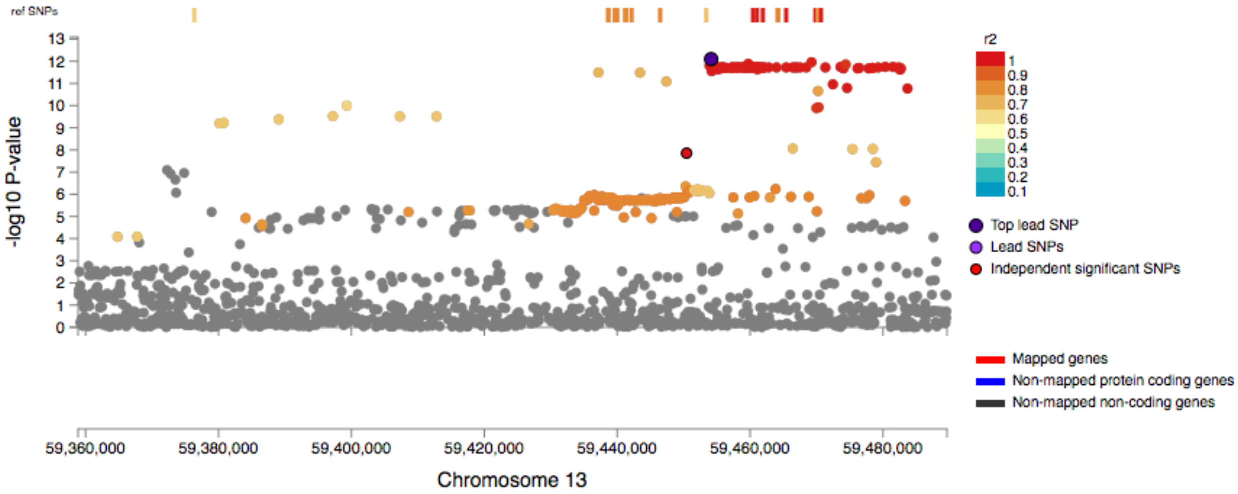

rs10986983

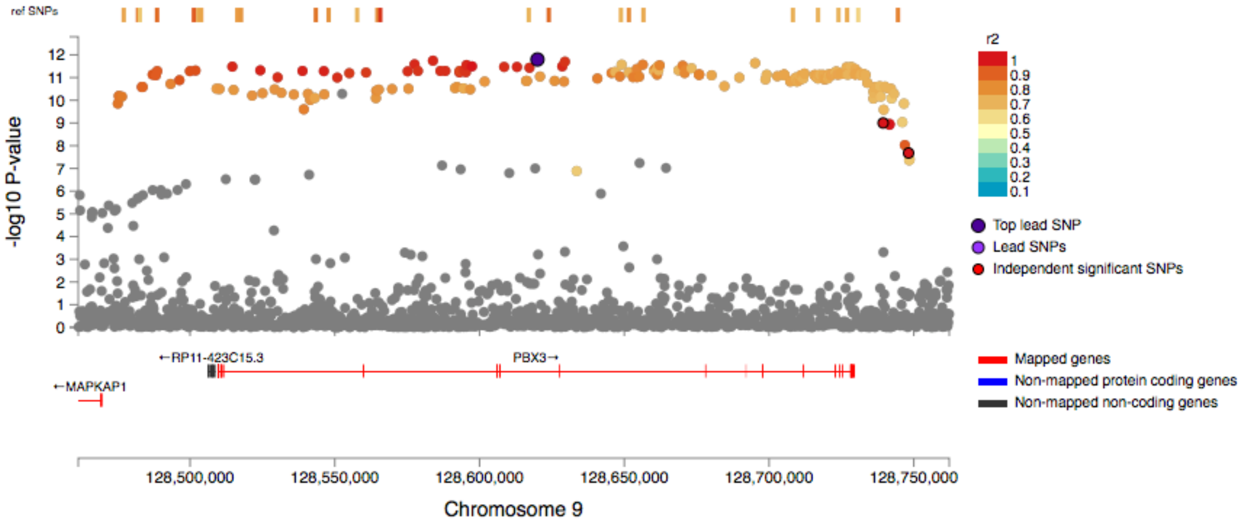

rs12682352

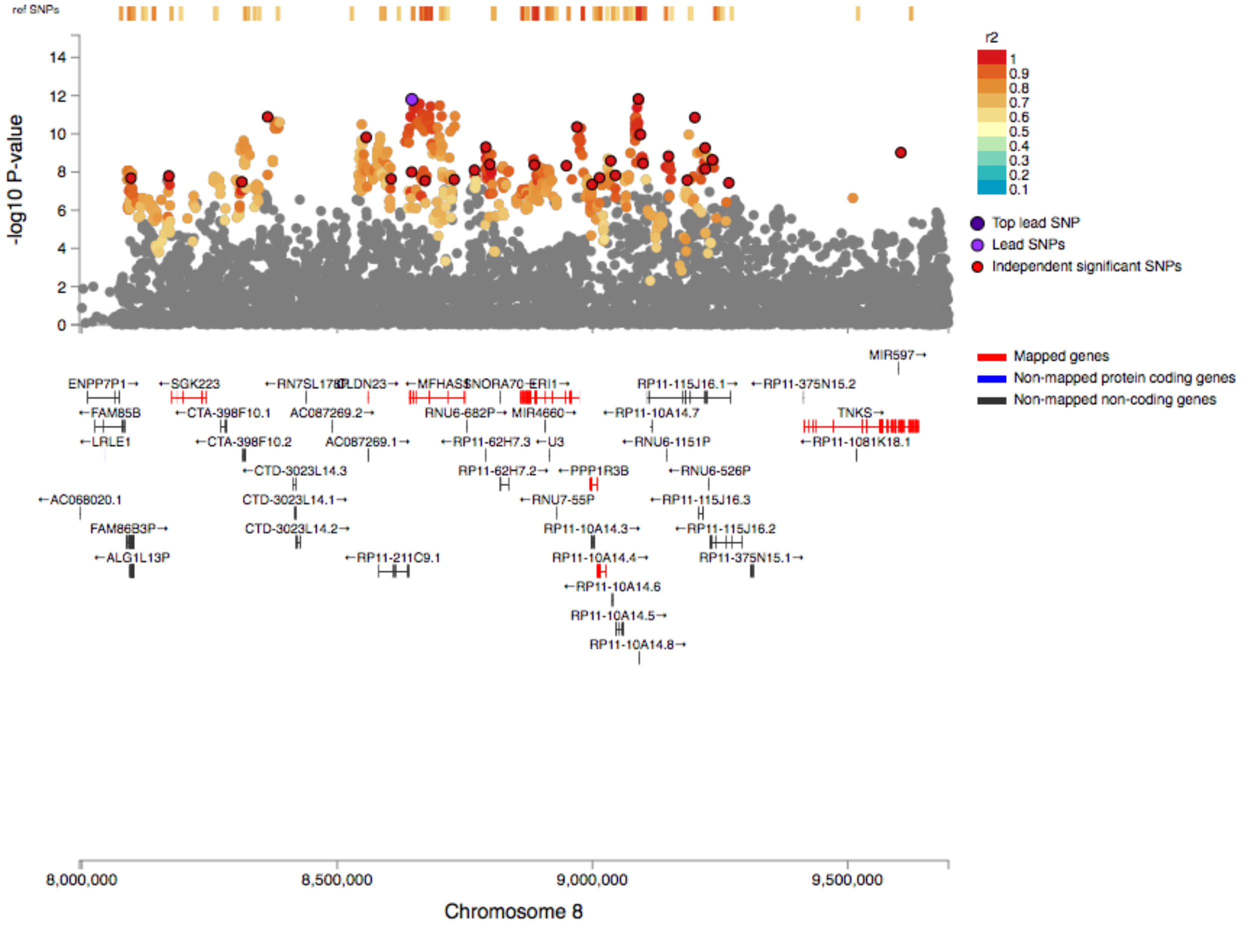

rs6699744

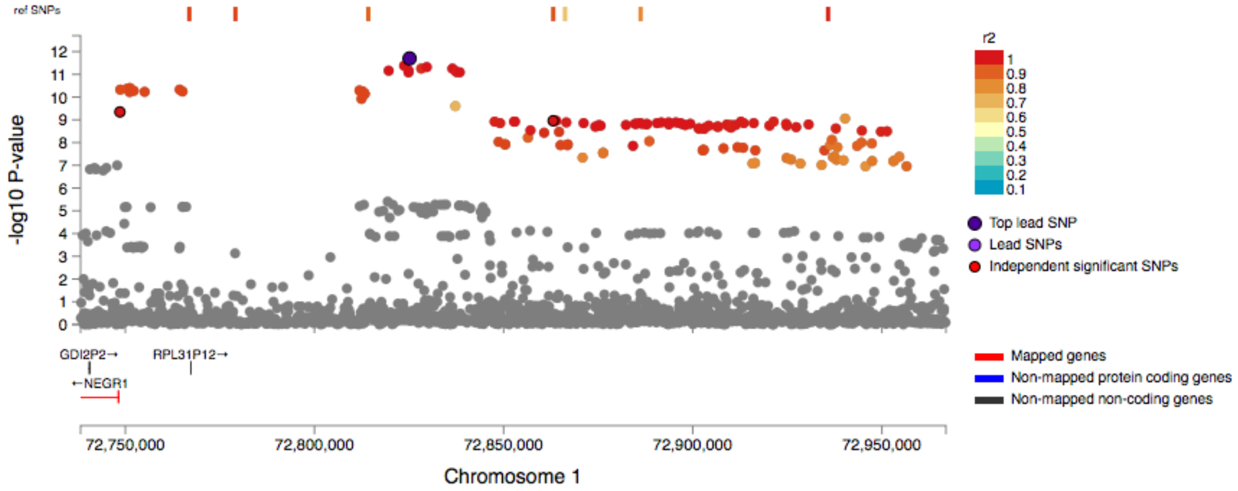

rs61656398

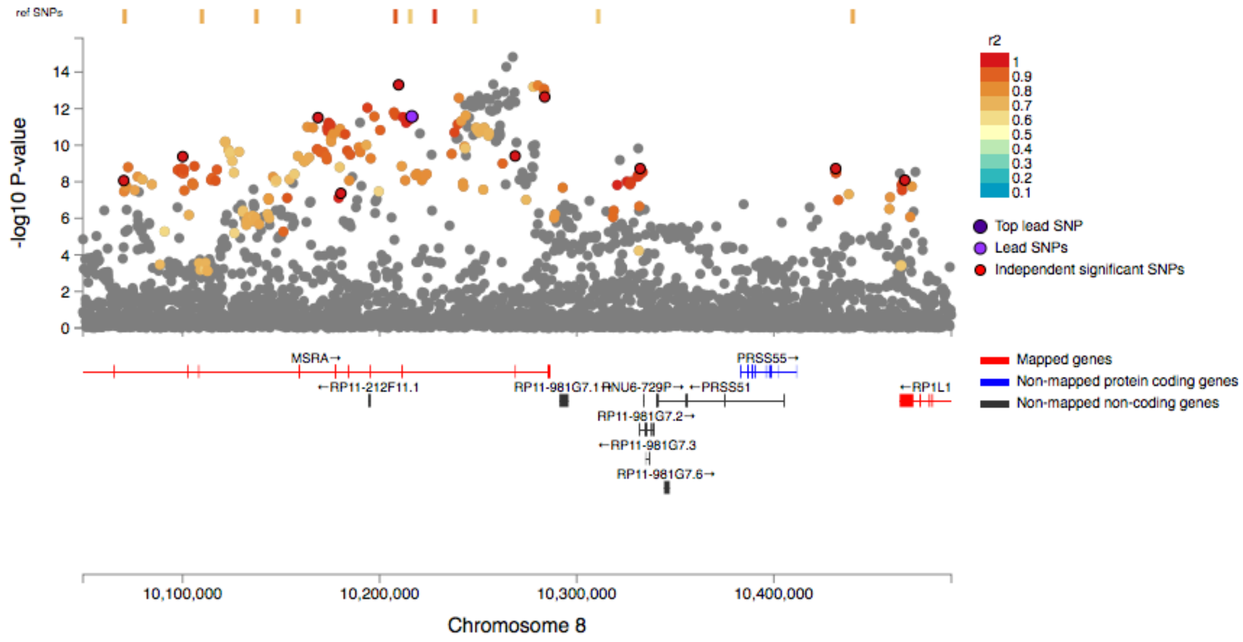
ref SNPs
-log10 P-value
0
2
4
6
8
10
12
14
r2
1
0.9
0.8
0.7
0.6
0.5
0.4
0.3
0.2
0.1
Top lead SNP
Lead SNPs
Independent significant SNPs
MSRA→
←RP11-212F11.1
RP11-981G7.1
RNU6-729P→
←PRSS51
PRSS55→
RP11-981G7.2→
←RP11-981G7.3
RP11-981G7.6→
←RP1L1
Mapped genes
Non-mapped protein coding genes
Non-mapped non-coding genes
10,100,000
10,200,000
10,300,000
10,400,000
Chromosome 8

rs16891727

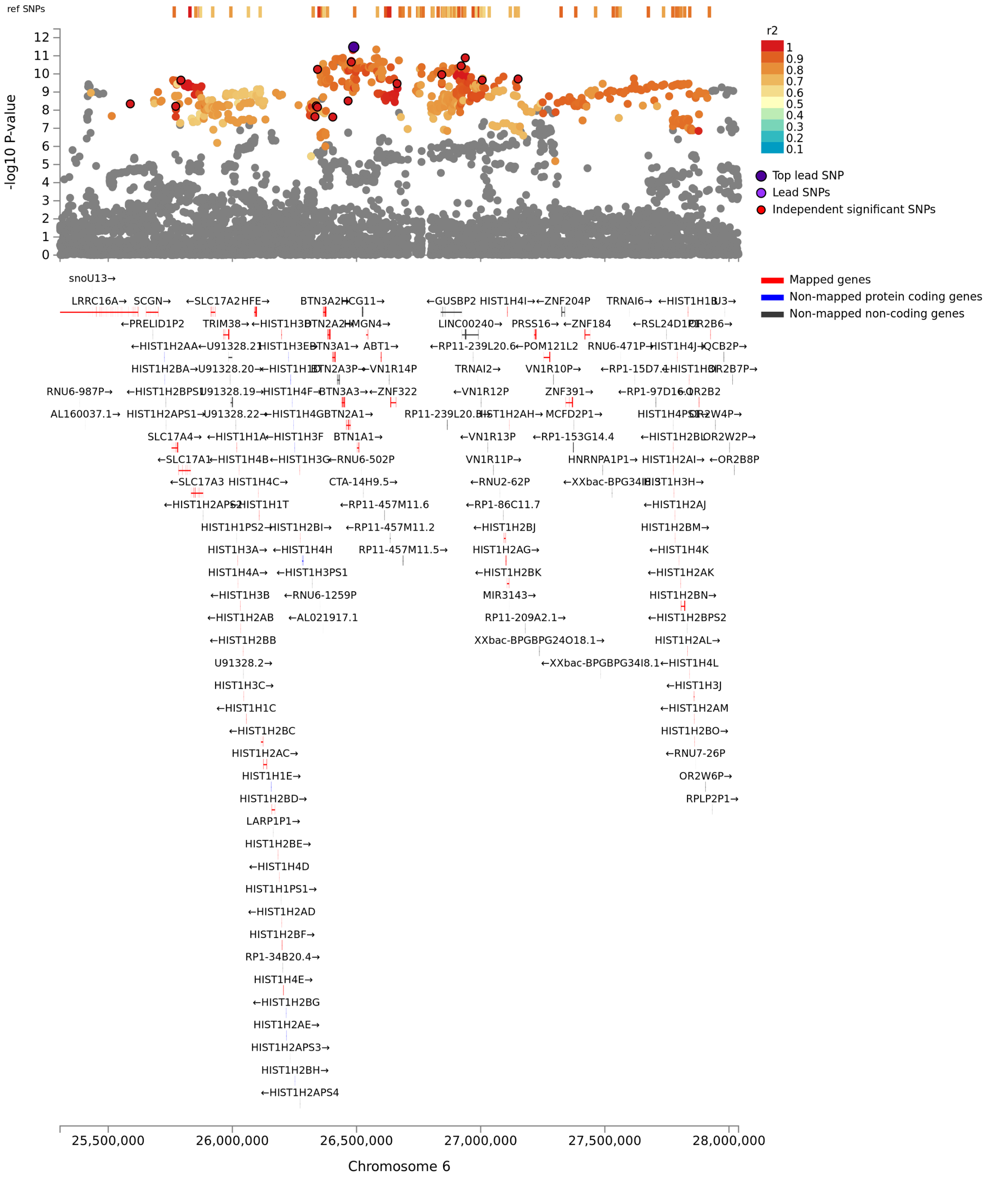
ref SNPs
-log10 P-value
r2
Top lead SNP
Lead SNPs
Independent significant SNPs
Mapped genes
Non-mapped protein coding genes
Non-mapped non-coding genes
25,500,000
26,000,000
26,500,000
27,000,000
27,500,000
28,000,000
Chromosome 6

rs1217105

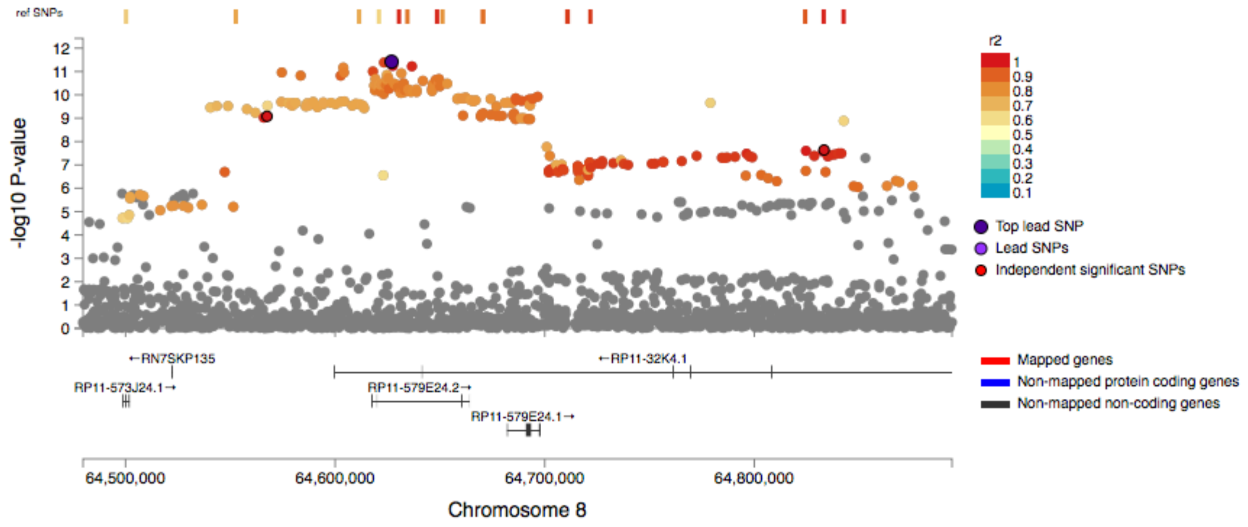

rs11859365

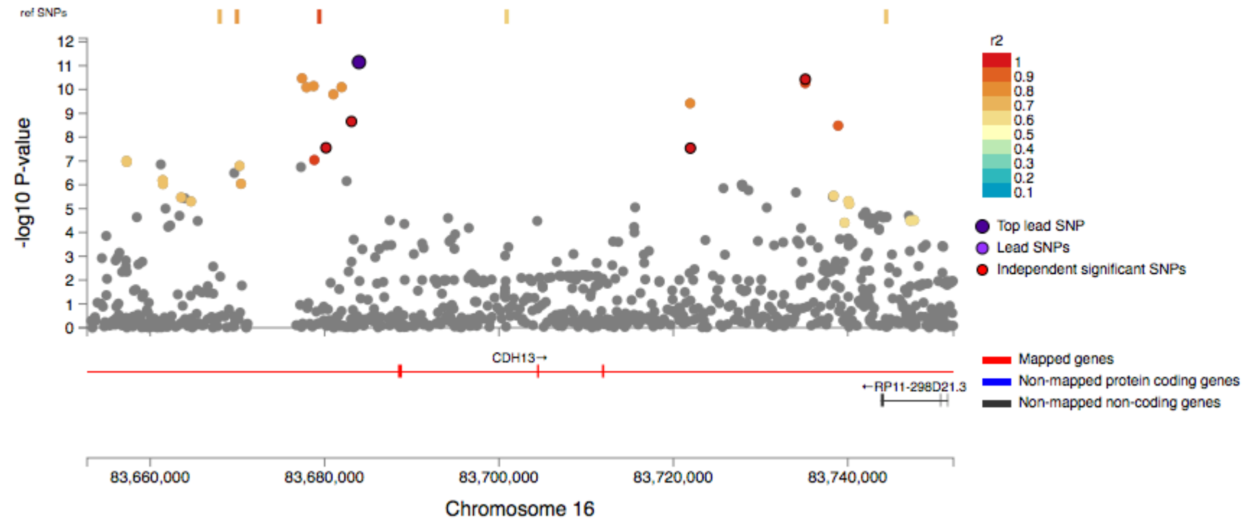

rs2425026

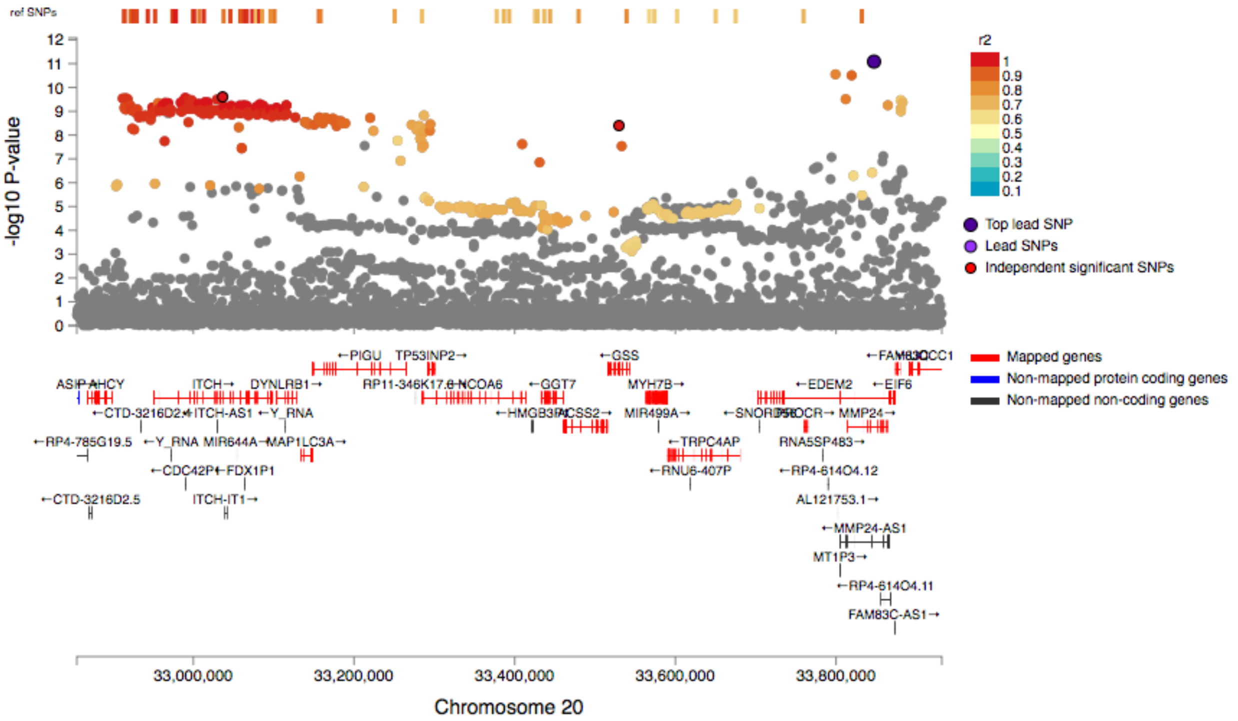

rs2413052

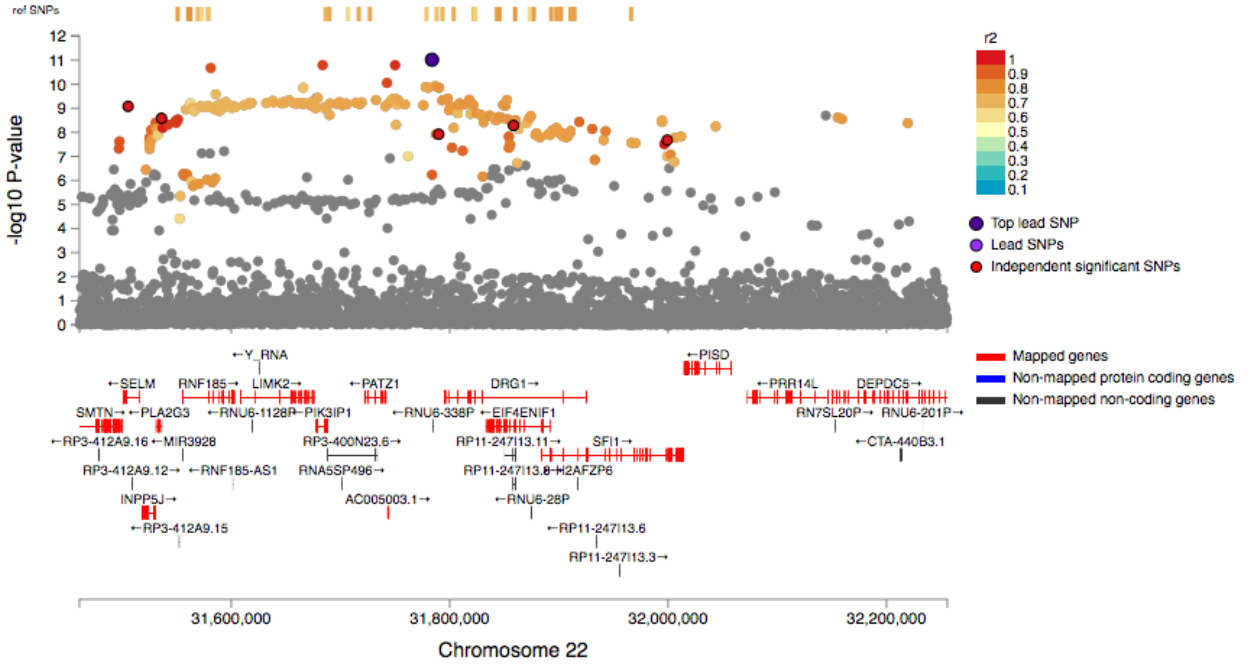

rs11706682

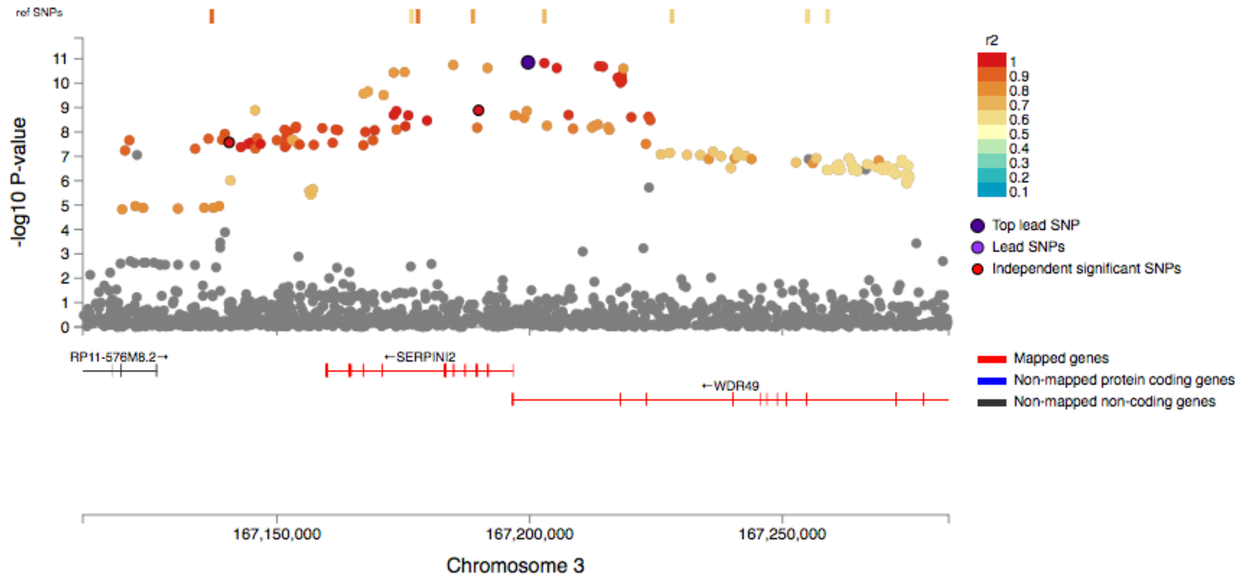

rs6756149

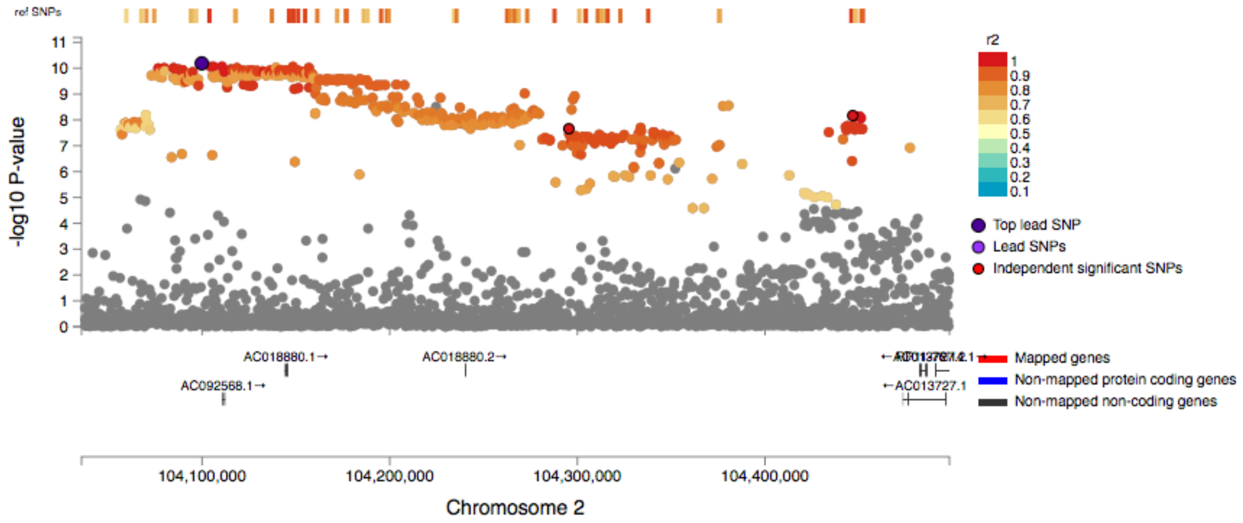

rs10510554

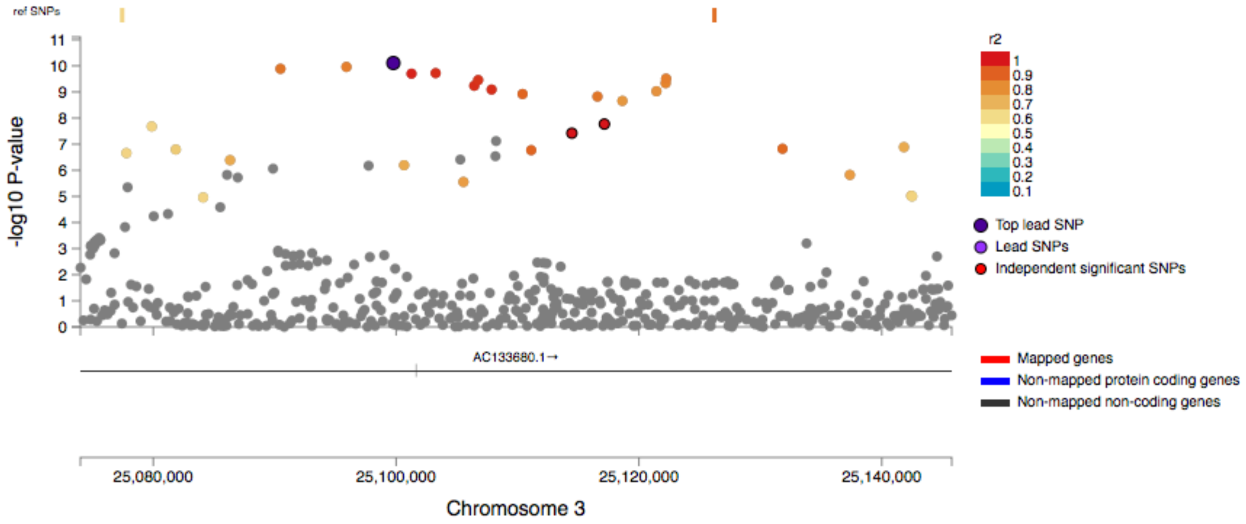

rs9362897

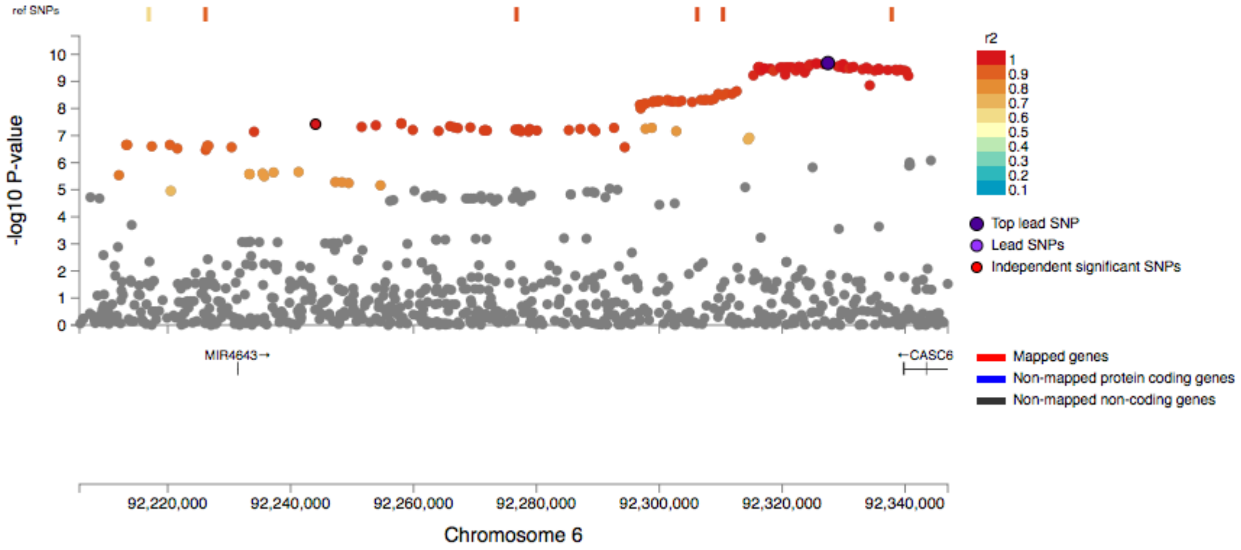

rs7752448

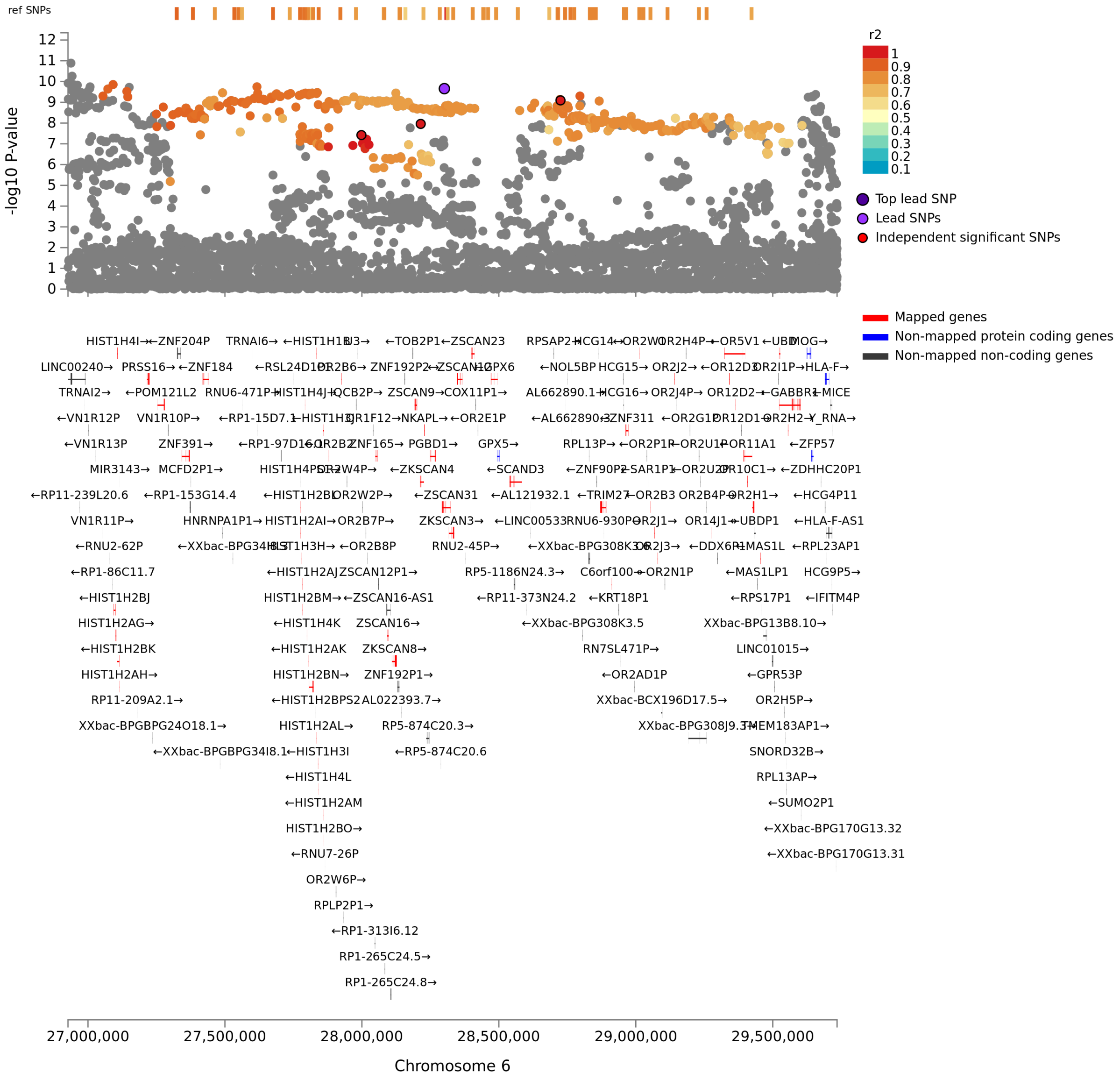

ref SNPs
-log10 P-value
r2
1
0.9
0.8
0.7
0.6
0.5
0.4
0.3
0.2
0.1
Top lead SNP
Lead SNPs
Independent significant SNPs
Mapped genes
Non-mapped protein coding genes
Non-mapped non-coding genes
27,000,000
27,500,000
28,000,000
28,500,000
29,000,000
29,500,000
Chromosome 6

rs6857

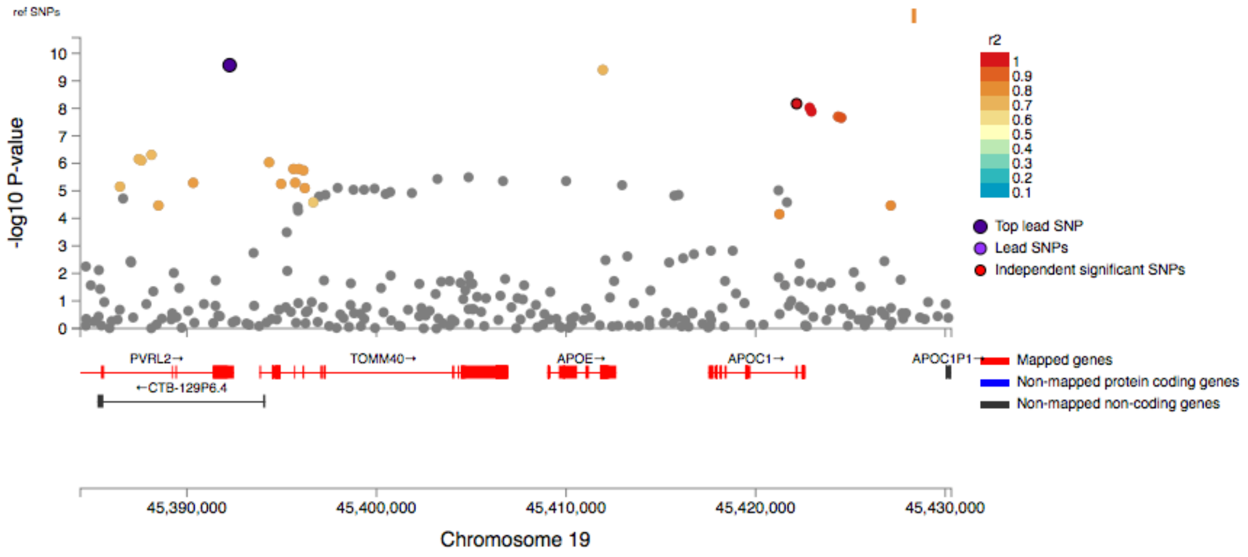

rs1410054

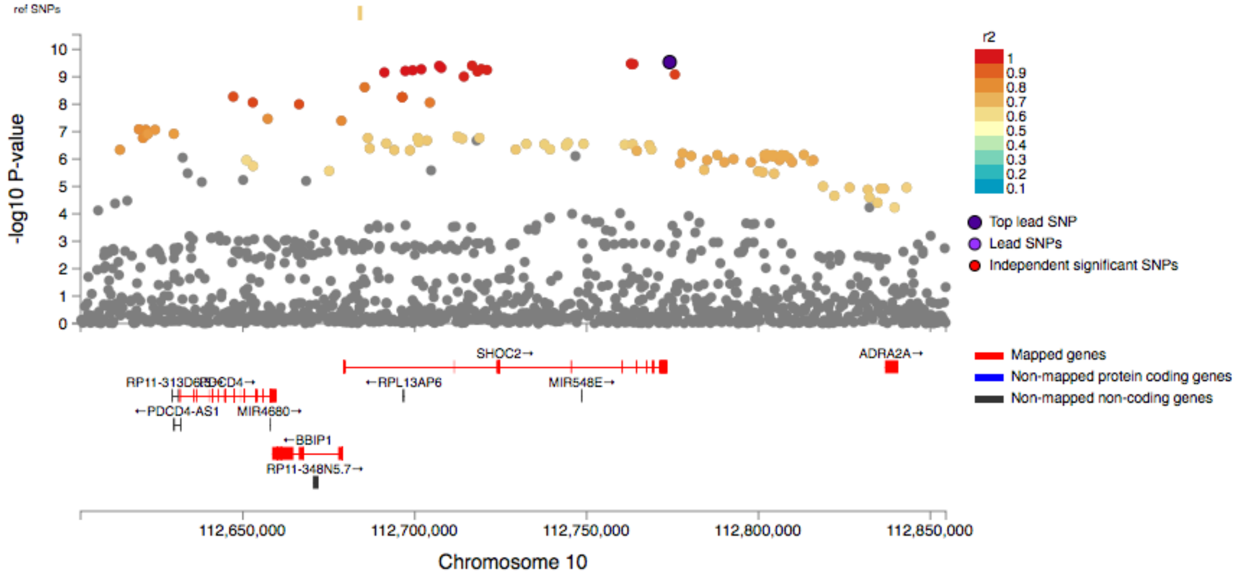

rs913455

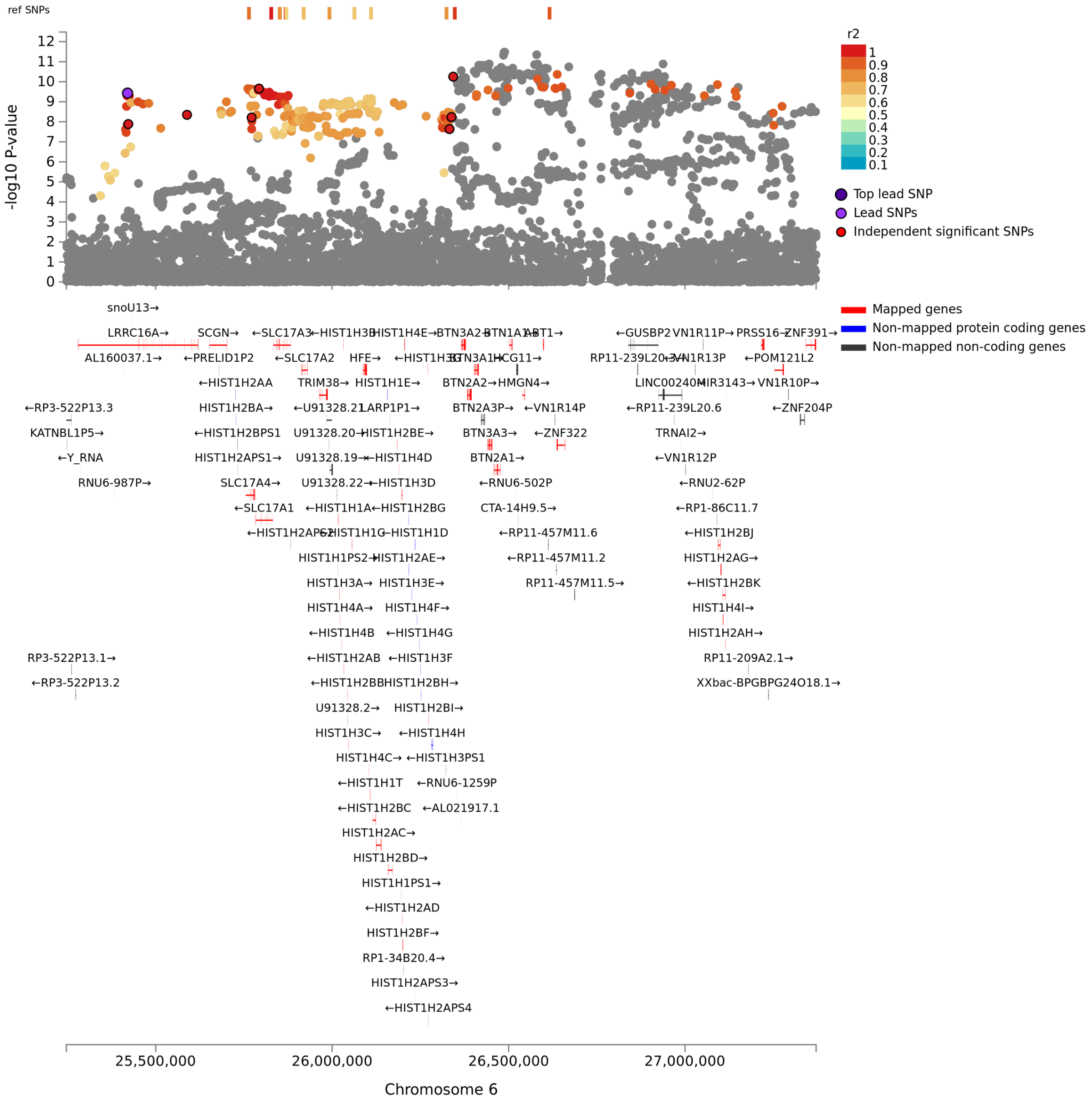
ref SNPs
-log10 P-value
0 1 2 3 4 5 6 7 8 9 10 11 12
r2
1 0.9 0.8 0.7 0.6 0.5 0.4 0.3 0.2 0.1
Top lead SNP
Lead SNPs
Independent significant SNPs
Mapped genes
Non-mapped protein coding genes
Non-mapped non-coding genes
snoU13→
LRRC16A→
SCGN→
←SLC17A3
←HIST1H3B
HIST1H4E→
BTN3A2→
BTN1A1
ABT1→
←GUSBP2
VN1R11P→
PRSS16→
←ZNF391→
AL160037.1→
←PRELID1P2
←SLC17A2
HFE→
←HIST1H3G
BTN3A1→
HCG11→
RP11-239L20.3
←VN1R13P
←POM121L2
←HIST1H2AA
TRIM38→
HIST1H1E→
BTN2A2→
HMGN4→
LINC00240
MIR3143→
VN1R10P→
←RP3-522P13.3
HIST1H2BA→
←U91328.21
LARP1P1→
BTN2A3P→
←VN1R14P
←RP11-239L20.6
←ZNF204P
KATNBL1P5→
←HIST1H2BPS1
U91328.20→
HIST1H2BE→
BTN3A3→
←ZNF322
TRNAI2→
←Y_RNA
HIST1H2APS1→
U91328.19→
←HIST1H4D
BTN2A1→
←VN1R12P
RNU6-987P→
SLC17A4→
U91328.22→
←HIST1H3D
←RNU6-502P
←RNU2-62P
←SLC17A1
←HIST1H1A
←HIST1H2BG
CTA-14H9.5→
←RP1-86C11.7
←HIST1H2APS2
←HIST1H1C
←HIST1H1D
←RP11-457M11.6
←HIST1H2BJ
HIST1H1PS2→
HIST1H2AE→
←RP11-457M11.2
HIST1H2AG→
HIST1H3A→
HIST1H3E→
RP11-457M11.5→
←HIST1H2BK
HIST1H4A→
HIST1H4F→
HIST1H4I→
←HIST1H4B
←HIST1H4G
HIST1H2AH→
RP3-522P13.1→
←HIST1H2AB
←HIST1H3F
RP11-209A2.1→
←RP3-522P13.2
←HIST1H2BB
HIST1H2BH→
XXbac-BPGBPG24O18.1→
U91328.2→
HIST1H2BI→
HIST1H3C→
←HIST1H4H
HIST1H4C→
←HIST1H3PS1
←HIST1H1T
←RNU6-1259P
←HIST1H2BC
←AL021917.1
HIST1H2AC→
HIST1H2BD→
HIST1H1PS1→
←HIST1H2AD
HIST1H2BF→
RP1-34B20.4→
HIST1H2APS3→
←HIST1H2APS4
25,500,000
26,000,000
26,500,000
27,000,000
Chromosome 6

rs4458235

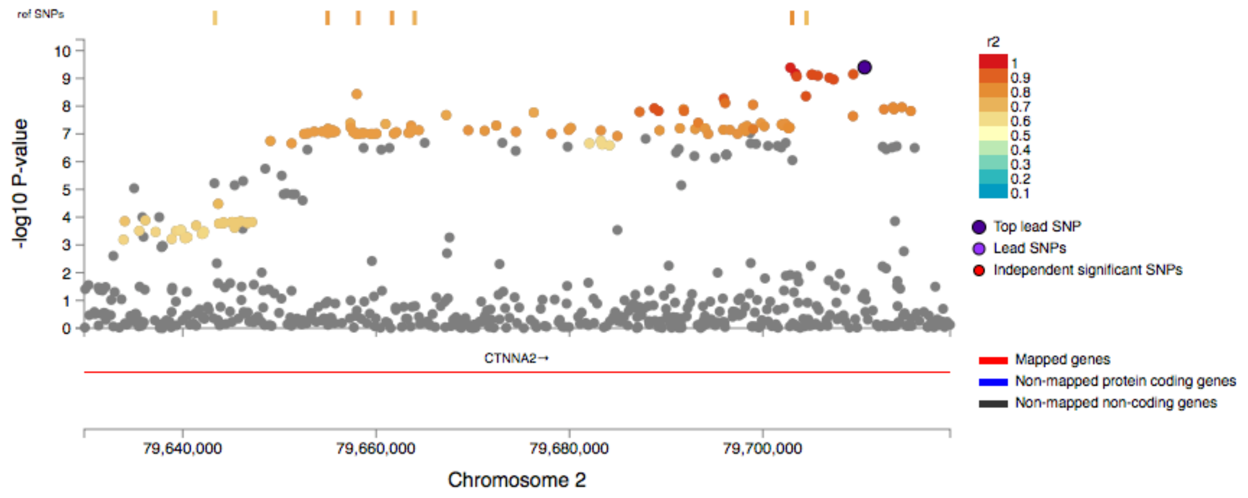

rs45501495

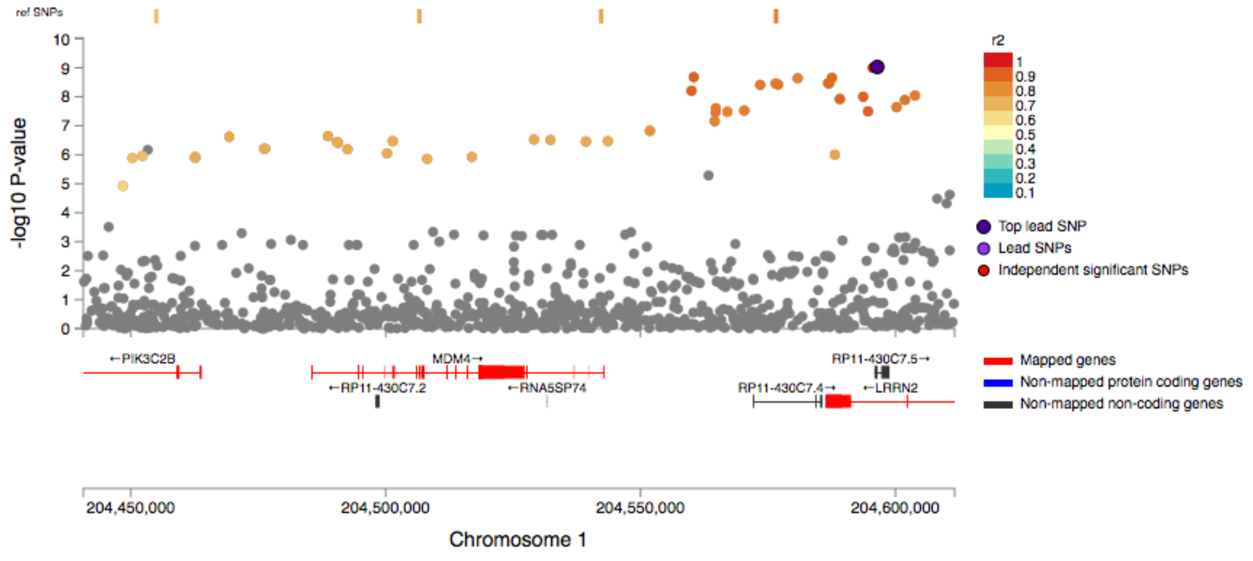

rs364926

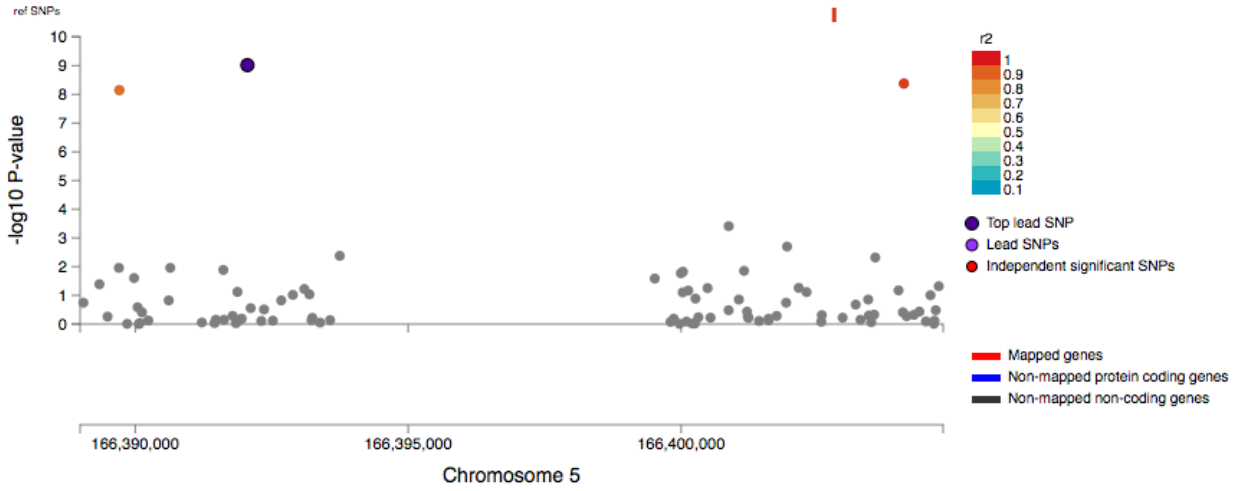

rs12906493

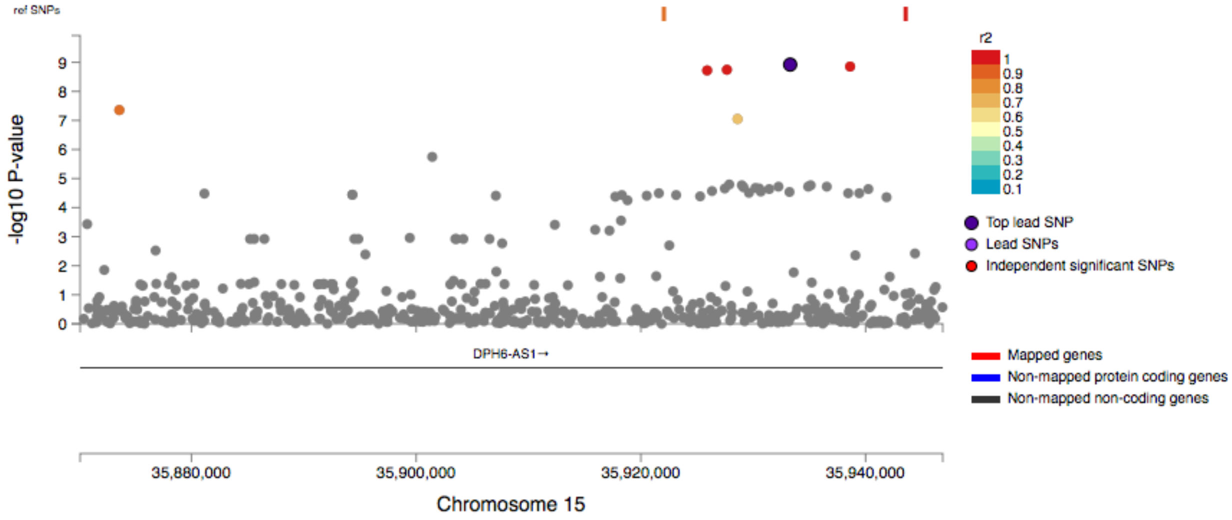

rs10741616

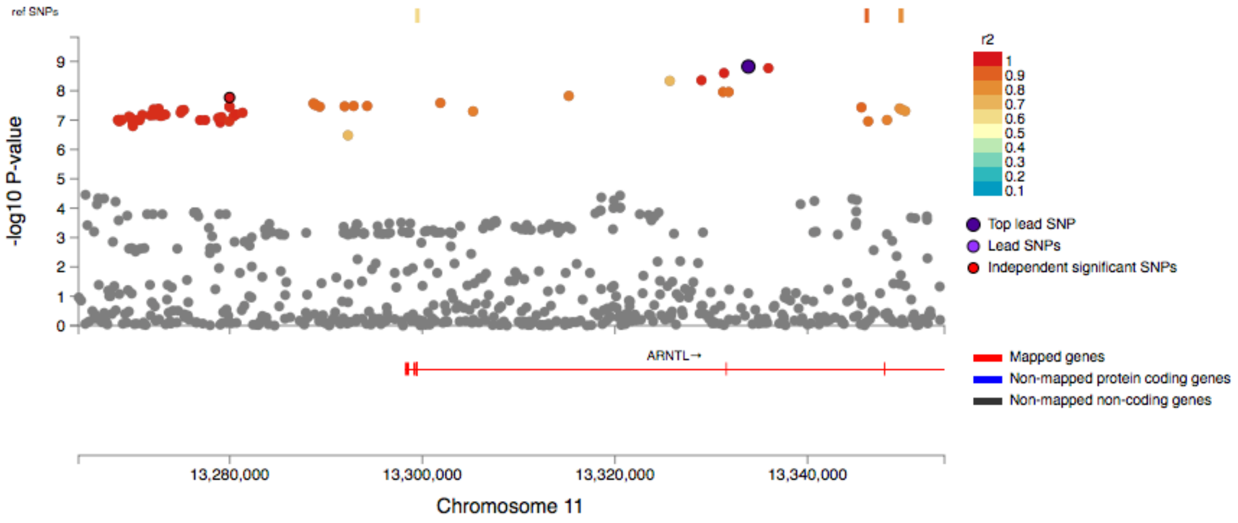

rs17049185

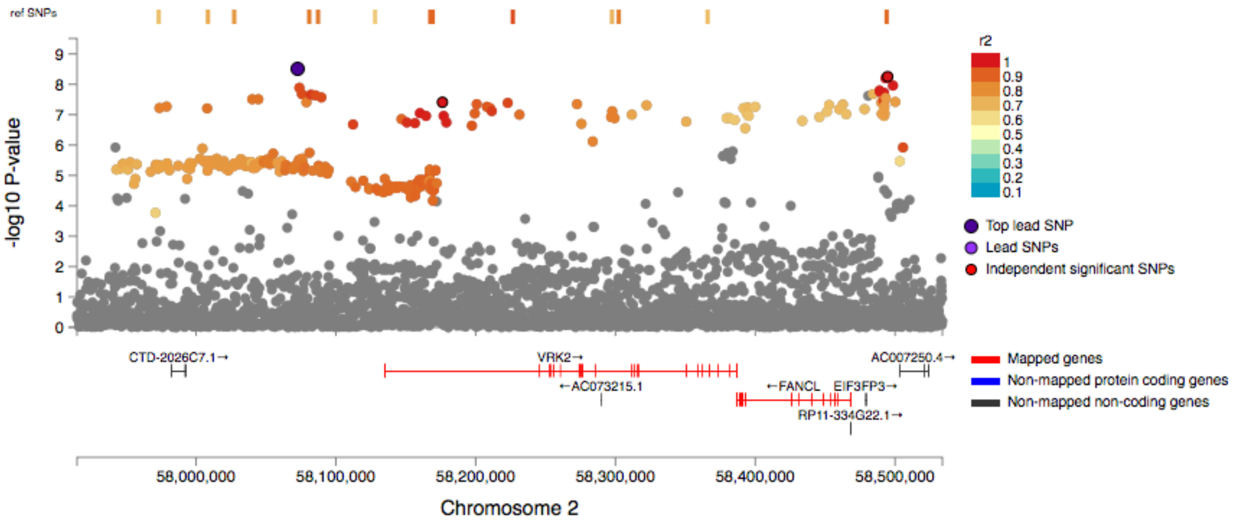

rs2463652

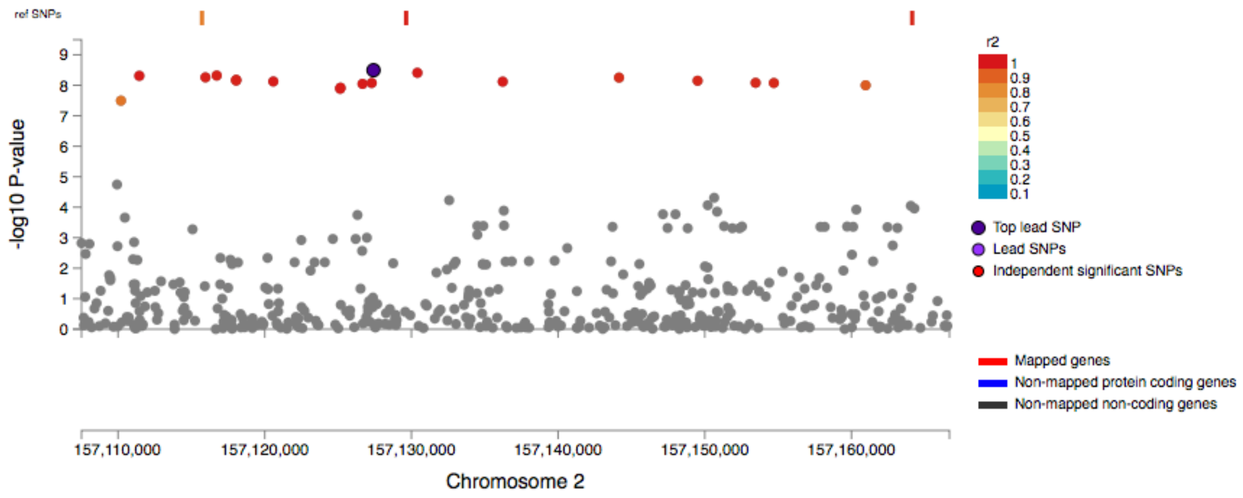

rs34634095

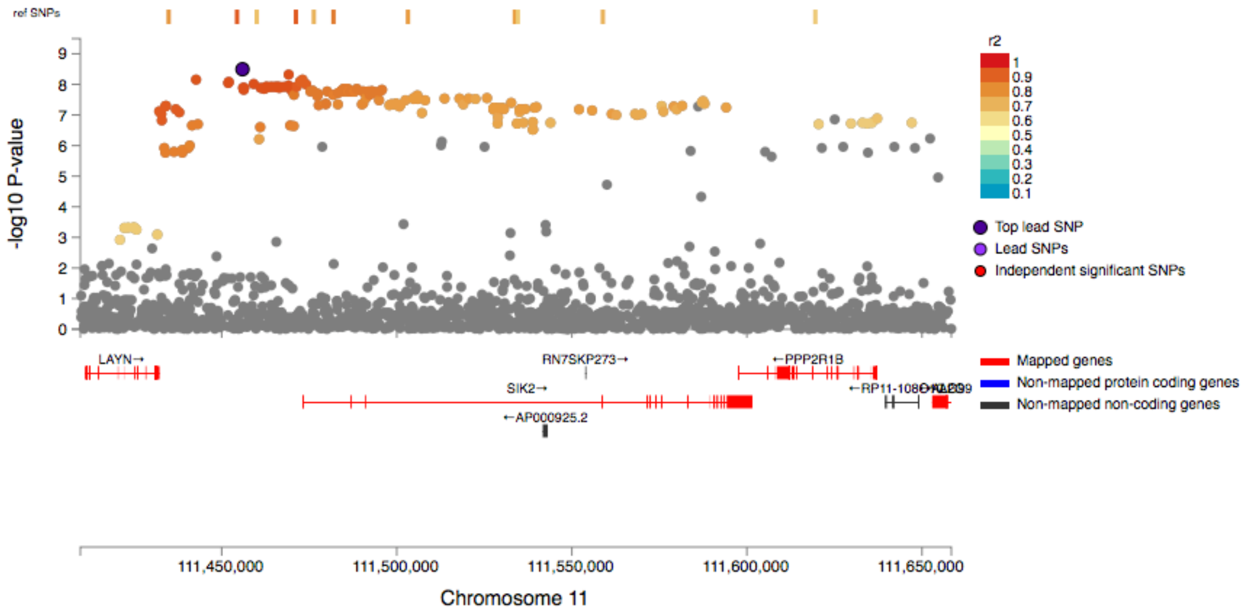

rs613597

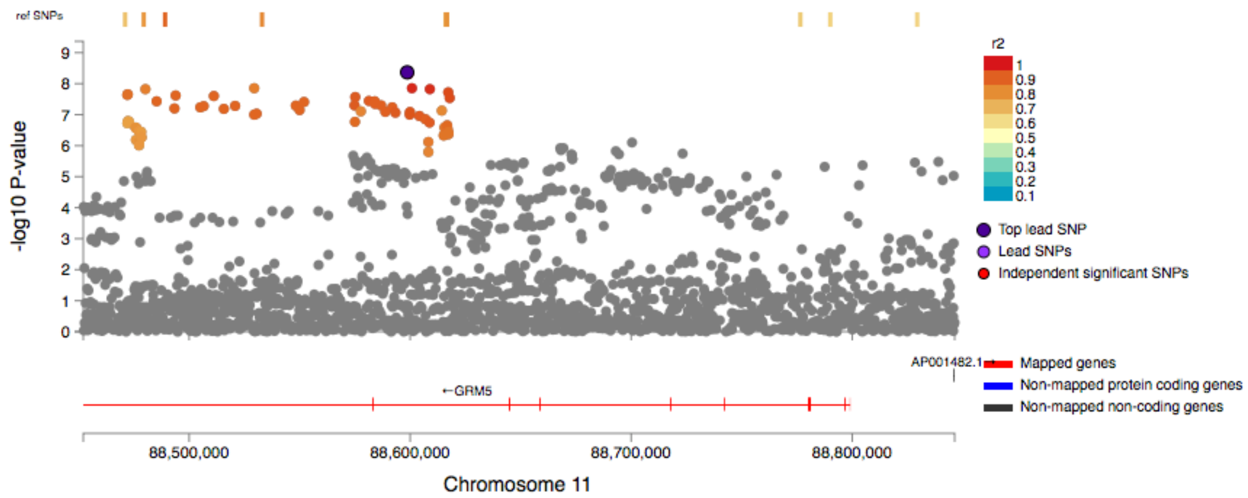

rs2915859

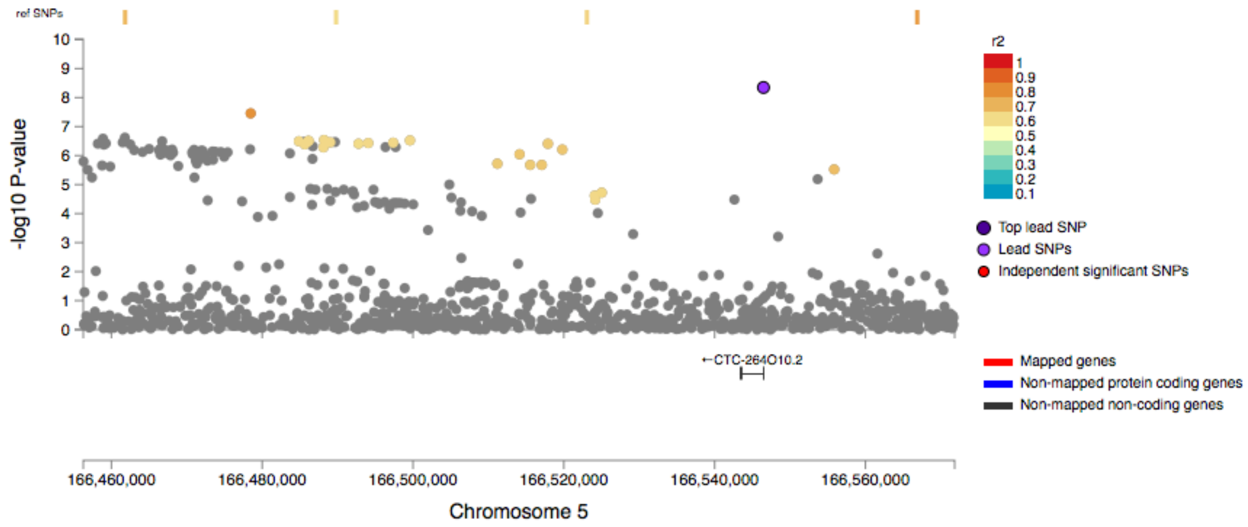

rs1946265

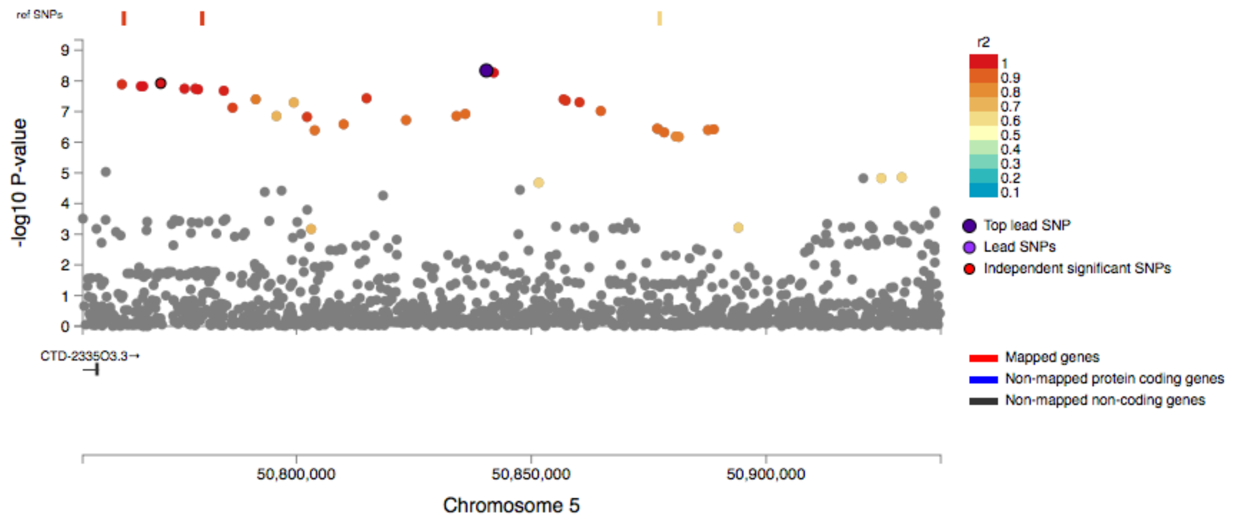

rs12826749

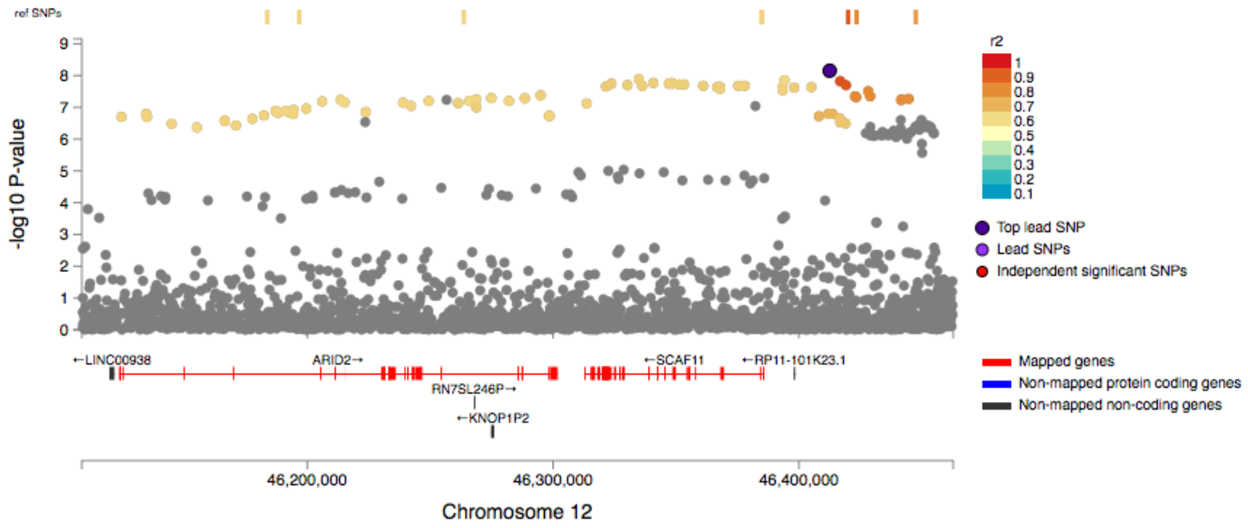

rs7737940

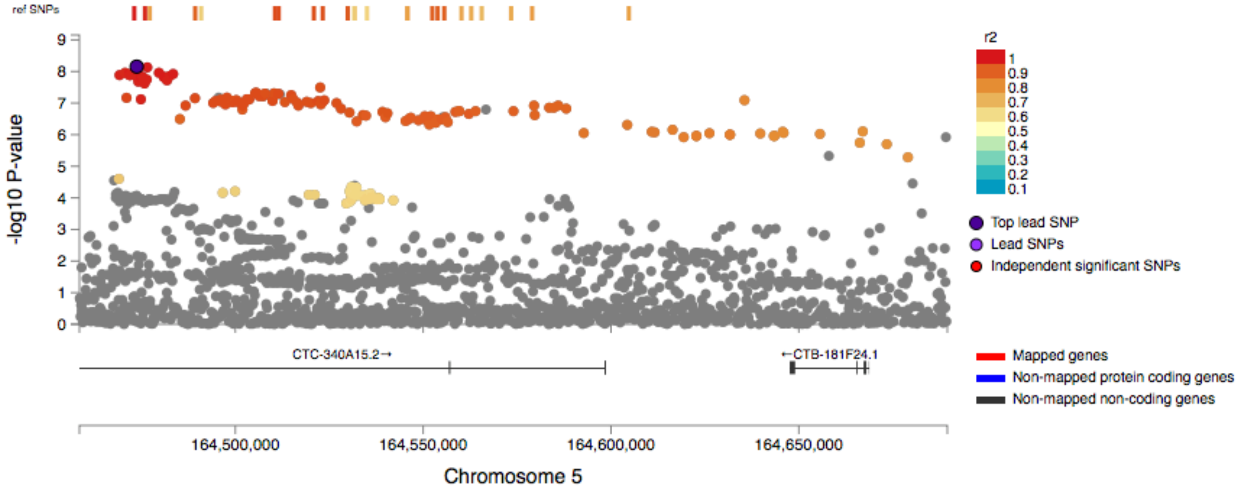

rs13082002

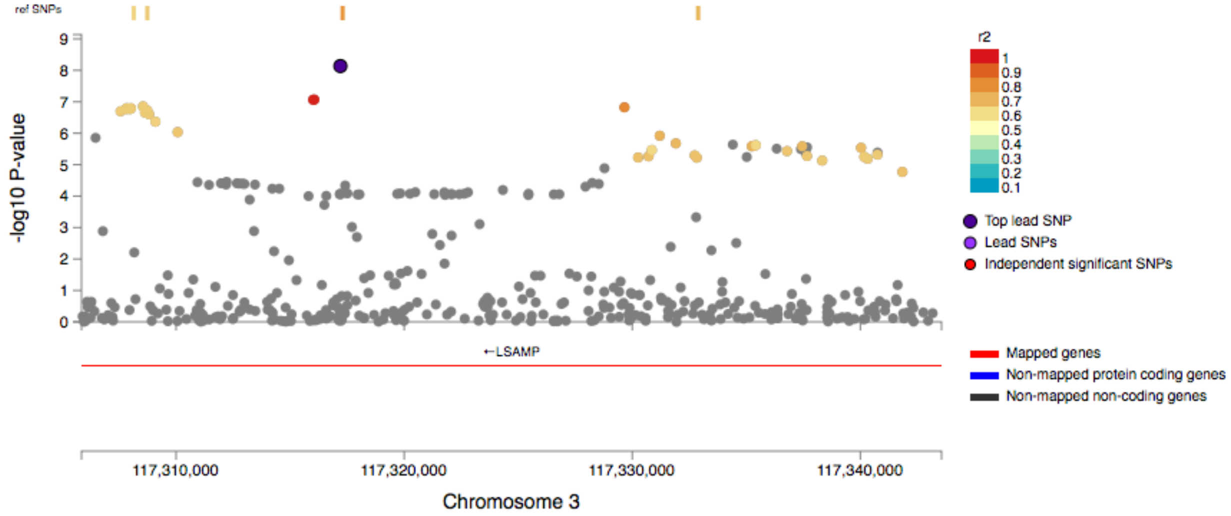

rs3117318

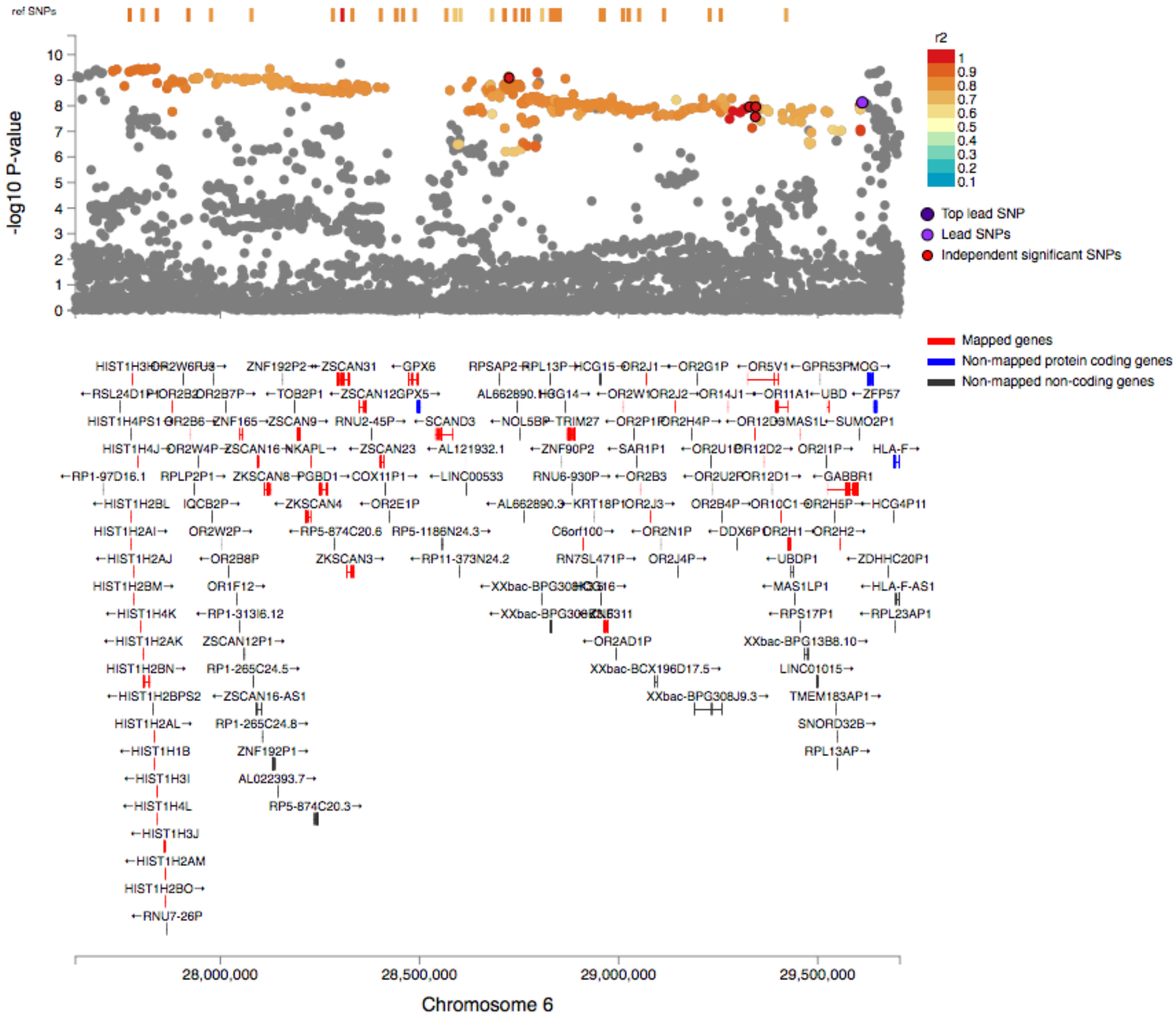
ref SNPs
-log10 P-value
10
9
8
7
6
5
4
3
2
1
0
r2
1
0.9
0.8
0.7
0.6
0.5
0.4
0.3
0.2
0.1
Top lead SNP
Lead SNPs
Independent significant SNPs
Mapped genes
Non-mapped protein coding genes
Non-mapped non-coding genes
ZNF192P2
ZSCAN31
GPX6
OR5V1
GPR53P
MOG
RSL24D1P1
TOB2P1
ZSCAN12
GPX5
OR11A1
UBD
ZFP57
ZNF165
ZSCAN9
RNU2-45P
SCAND3
NOL5BP
TRIM27
MAS1L
SUMO2P1
ZSCAN16
NKAPL
ZSCAN23
AL121932.1
ZNF90P2
SAR1P1
OR2U1
OR2D2
OR2I1P
HLA-F
RP1-97D16.1
RPLP2P1
ZKSCAN8
PGBD1
COX11P1
LINC00533
RNU6-930P
OR2B3
OR2U2P
OR12D1
GABBR1
HIST1H2BL
IQCB2P
ZKSCAN4
OR2E1P
AL662890.3
KRT18P1
OR2J3
OR2B4P
OR10C1
OR2H5P
HCG4P11
HIST1H2AI
OR2W2P
RP5-874C20.6
RP5-1186N24.3
C6orf100
OR2N1P
DDX6P
OR2H1
OR2H2
HIST1H2AJ
OR2B8P
ZKSCAN3
RP11-373N24.2
RN7SL471P
OR2J4P
UBDP1
ZDHHC20P1
HIST1H2BM
OR1F12
MAS1LP1
HLA-F-AS1
HIST1H4K
RP1-313I6.12
RPS17P1
RPL23AP1
HIST1H2AK
ZSCAN12P1
OR2AD1P
XXbac-BPG13B8.10
HIST1H2BN
RP1-265C24.5
XXbac-BCX196D17.5
LINC01015
HIST1H2BPS2
ZSCAN16-AS1
XXbac-BPG308J9.3
TMEM183AP1
HIST1H2AL
RP1-265C24.8
SNORD32B
HIST1H1B
ZNF192P1
RPL13AP
HIST1H3I
AL022393.7
HIST1H4L
RP5-874C20.3
HIST1H3J
HIST1H2AM
HIST1H2BO
RNU7-26P
28,000,000
28,500,000
29,000,000
29,500,000
Chromosome 6

rs3800314

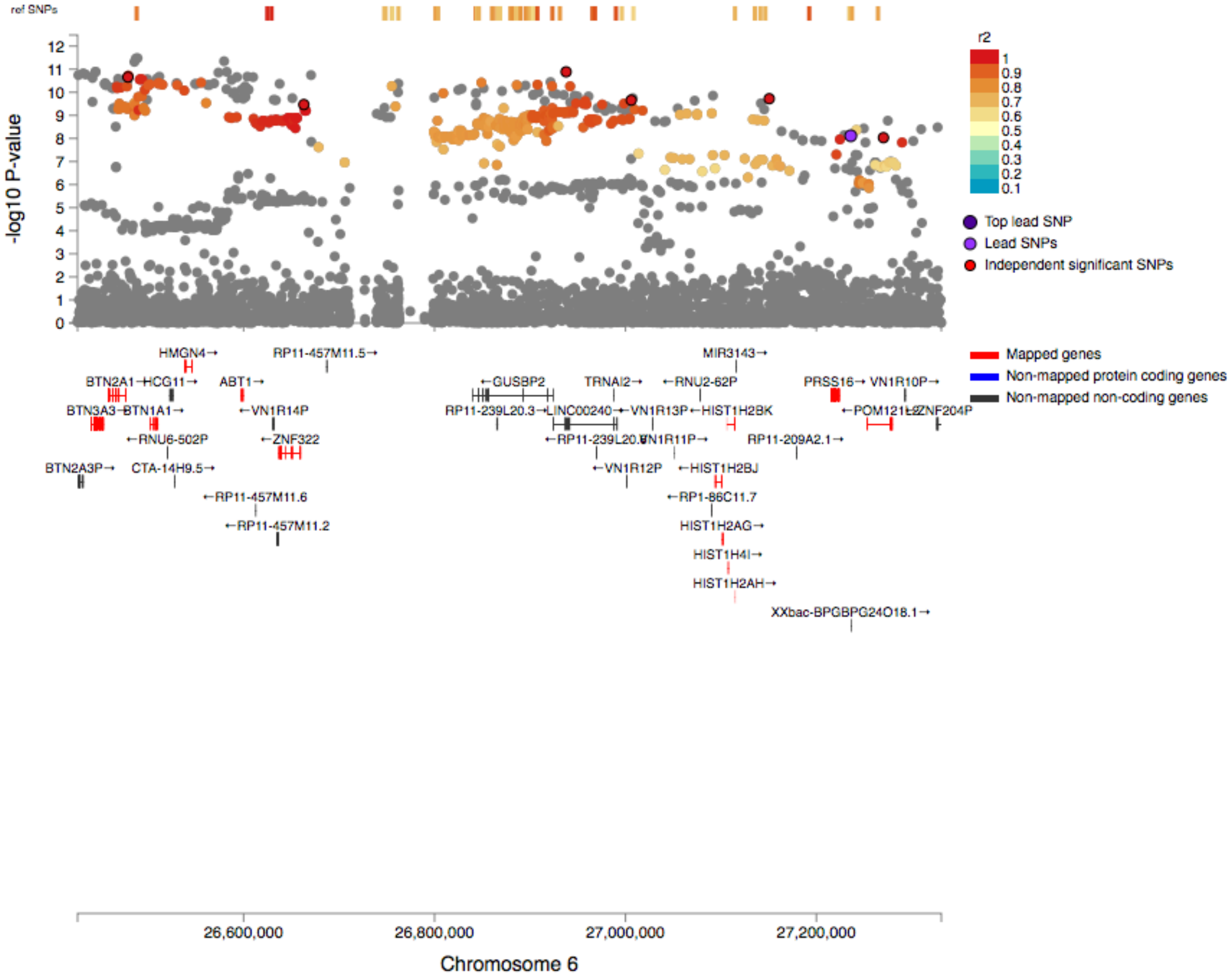

rs4278546

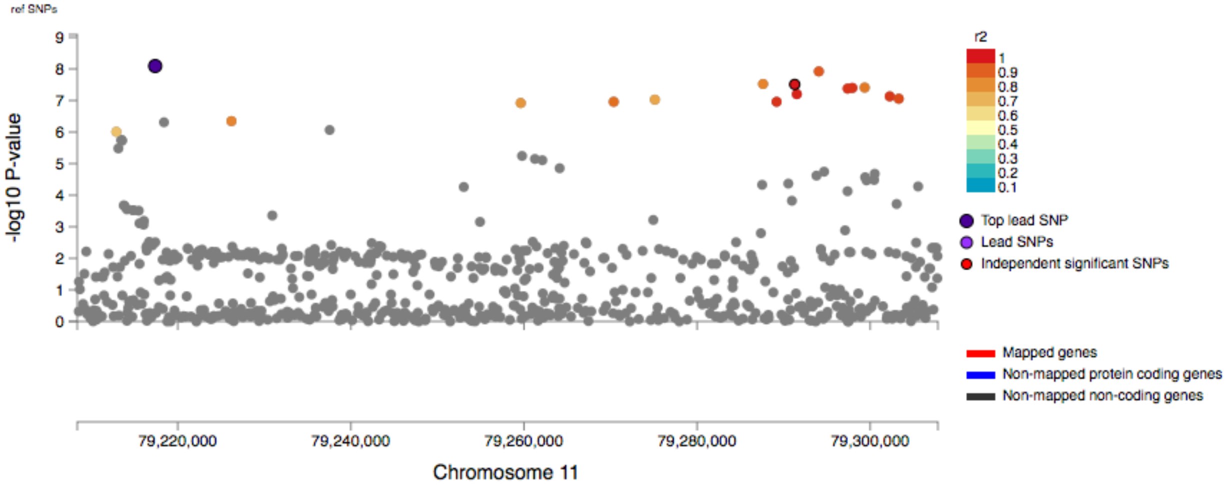

rs80264330

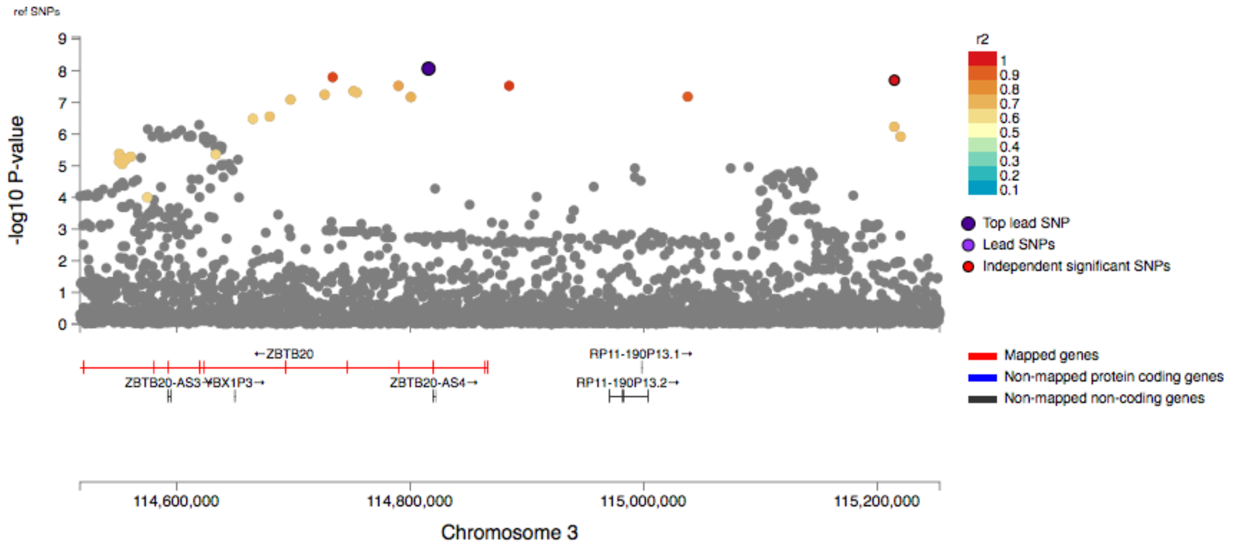

rs16959955

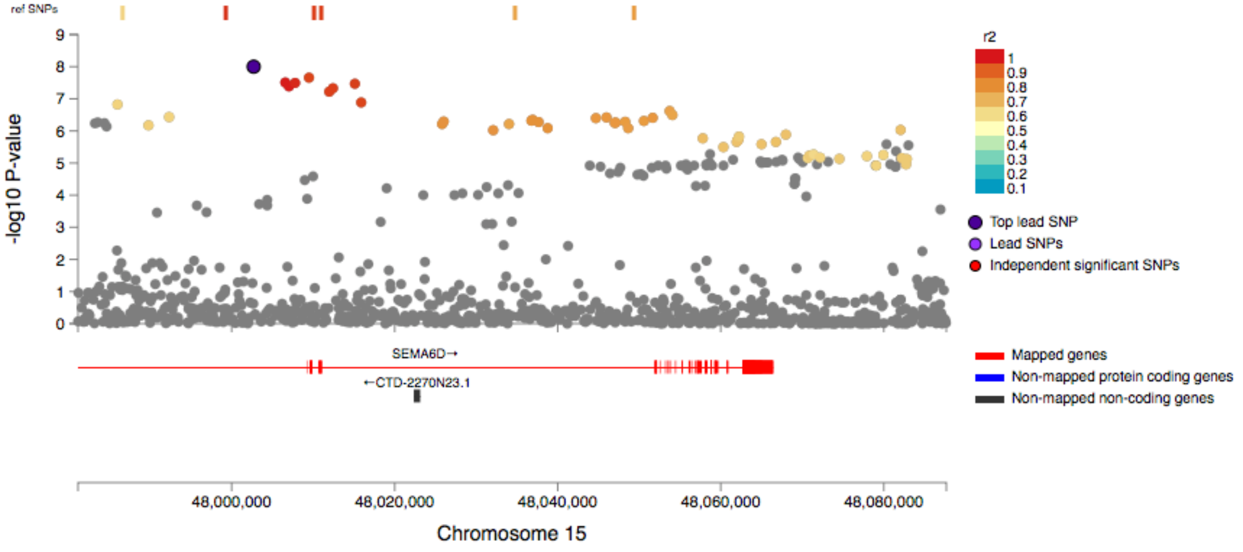

rs1897495

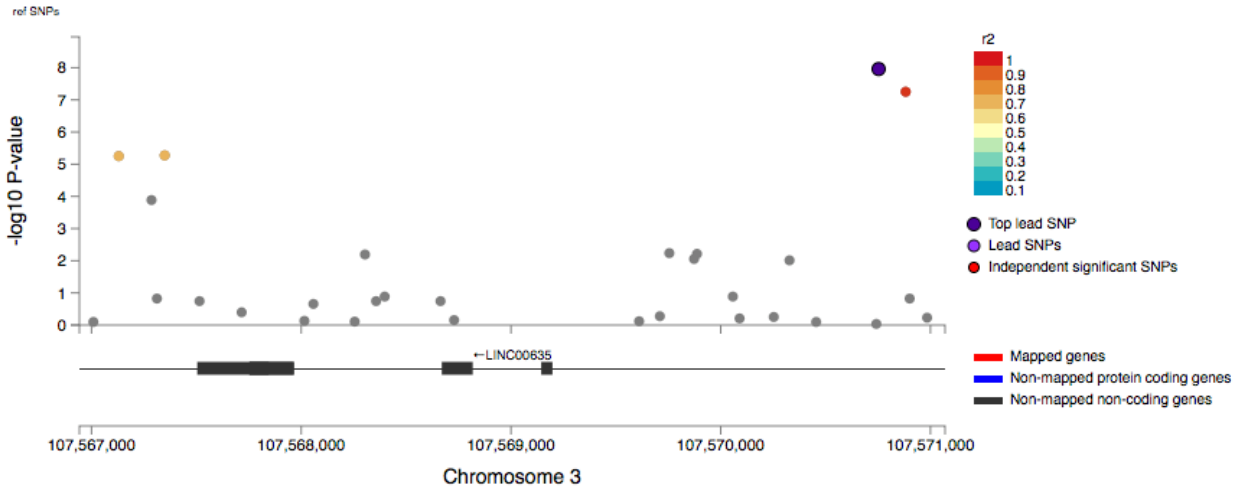

rs200110162

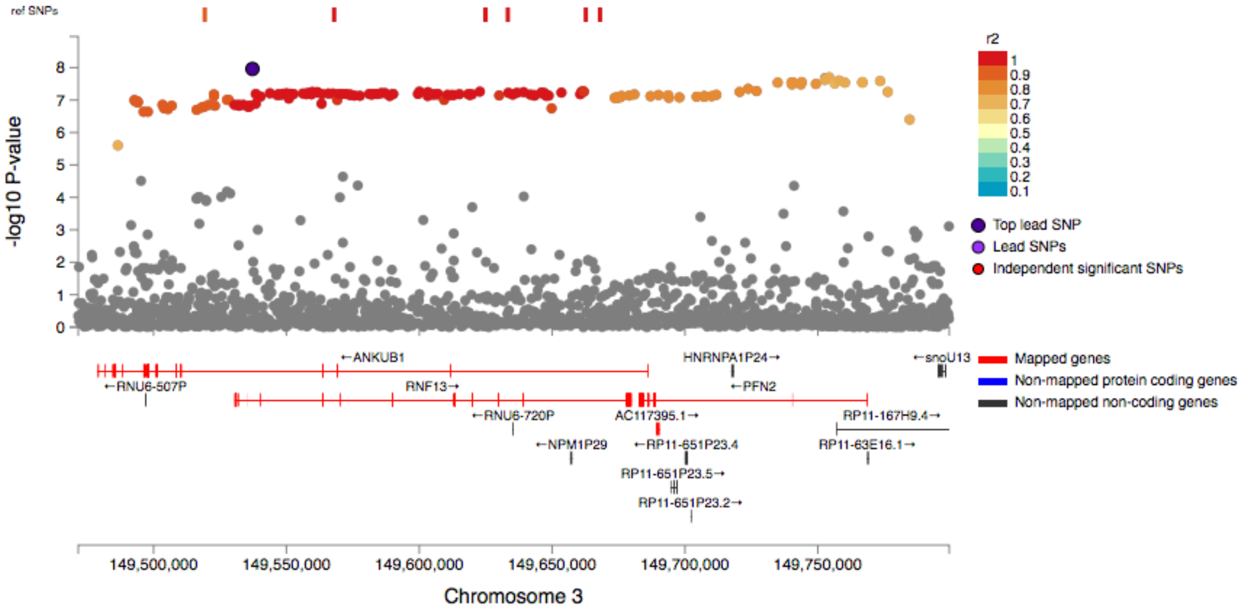

rs12700239

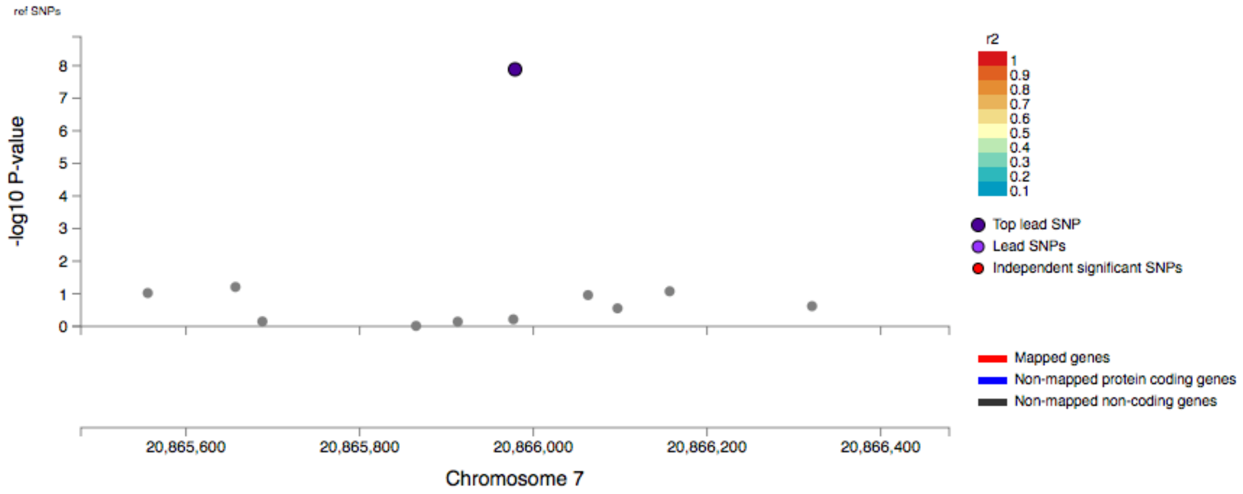
ref SNPs
-log10 P-value
r2
Top lead SNP
Lead SNPs
Independent significant SNPs
Mapped genes
Non-mapped protein coding genes
Non-mapped non-coding genes
20,865,600
20,865,800
20,866,000
20,866,200
20,866,400
Chromosome 7

rs2498381

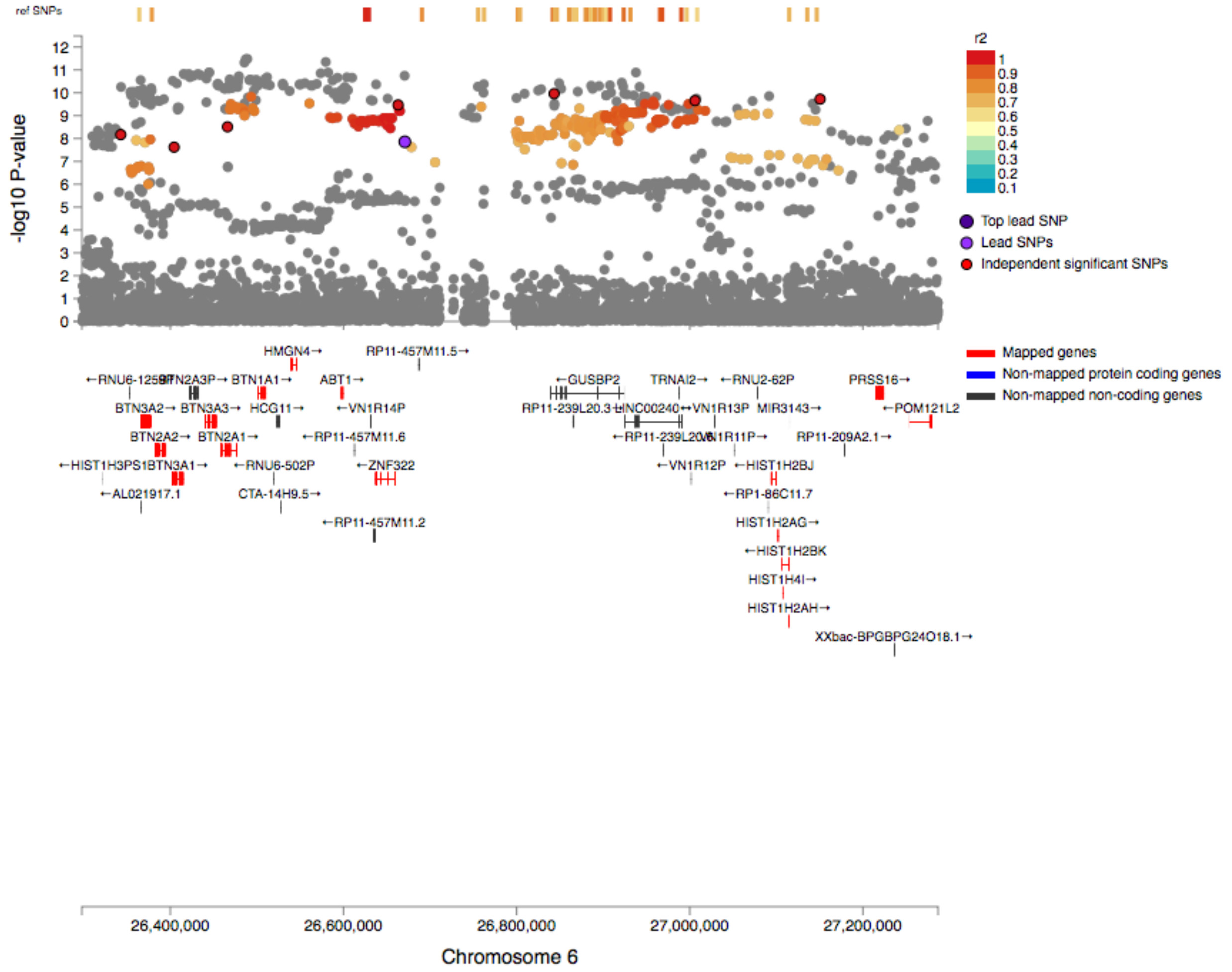
ref SNPs
-log10 P-value
r2
Top lead SNP
Lead SNPs
Independent significant SNPs
Mapped genes
Non-mapped protein coding genes
Non-mapped non-coding genes
HMGN4→
RP11-457M11.5→
ABT1→
←VN1R14P
←RP11-457M11.6
←ZNF322
←RP11-457M11.2
HCG11→
←RNU6-502P
CTA-14H9.5→
←GUSBP2
TRNAI2→
←RNU2-62P
PRSS16→
←POM121L2
MIR3143→
RP11-209A2.1→
←VN1R12P
←HIST1H2BJ
←RP1-86C11.7
HIST1H2AG→
←HIST1H2BK
HIST1H4I→
HIST1H2AH→
XXbac-BPGBPG24O18.1→
26,400,000
26,600,000
26,800,000
27,000,000
27,200,000
Chromosome 6

rs9955276

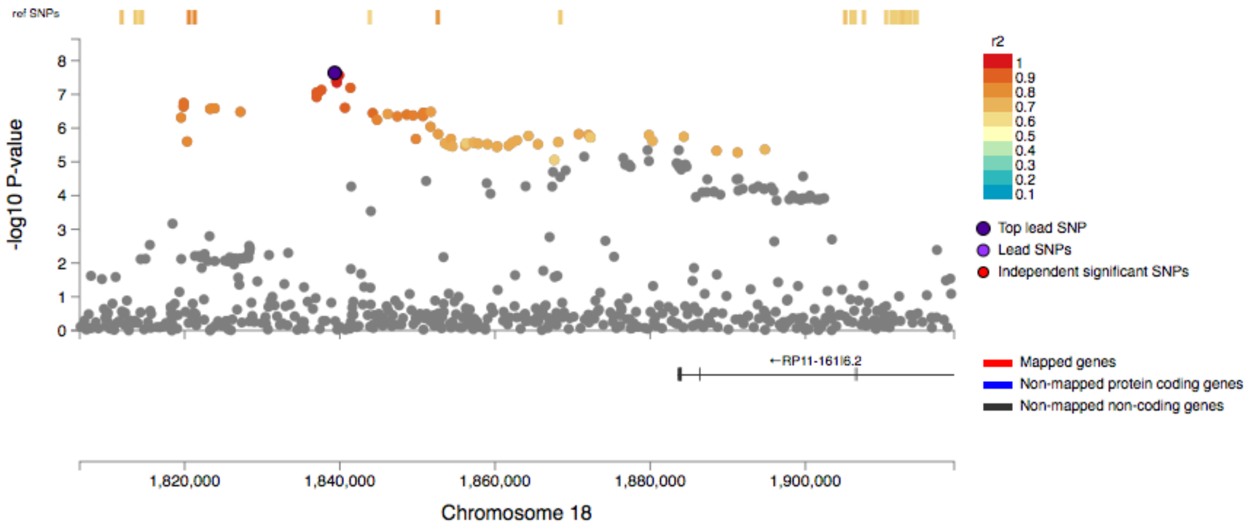

rs13160801

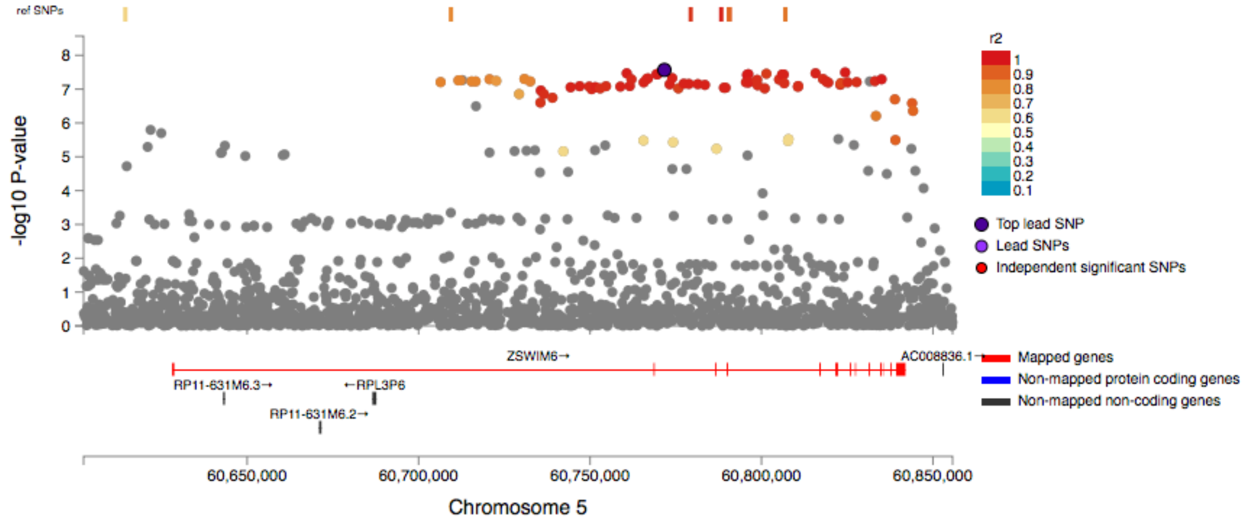

rs62132802

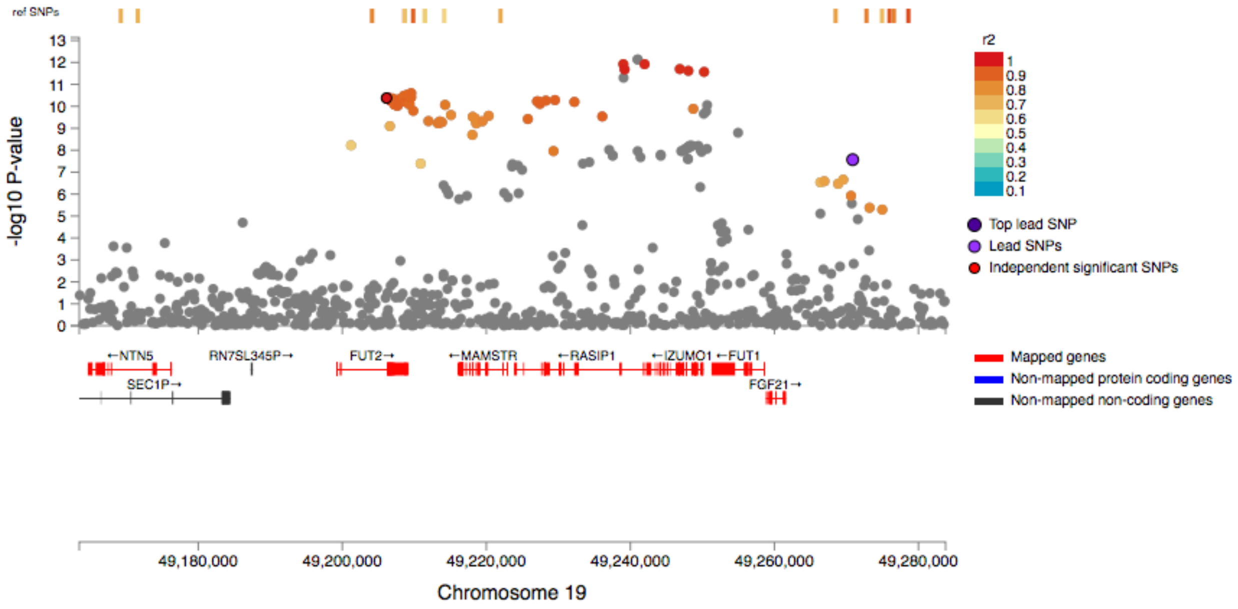

rs10180461

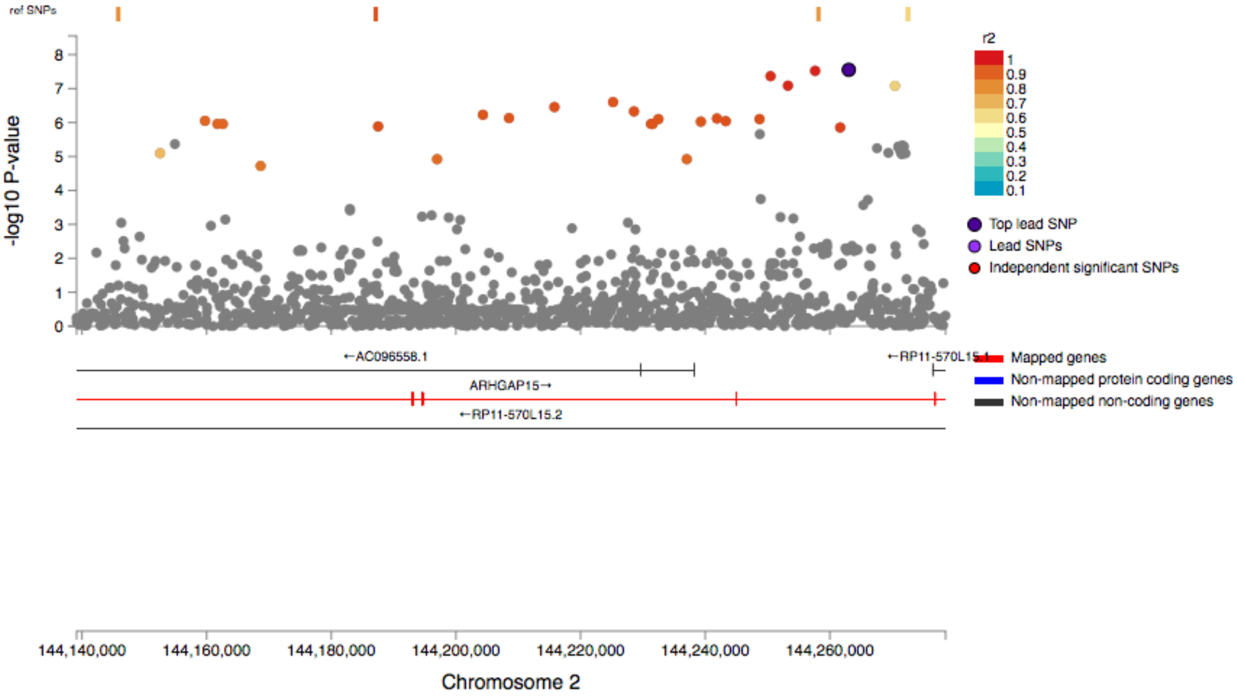

rs7978702

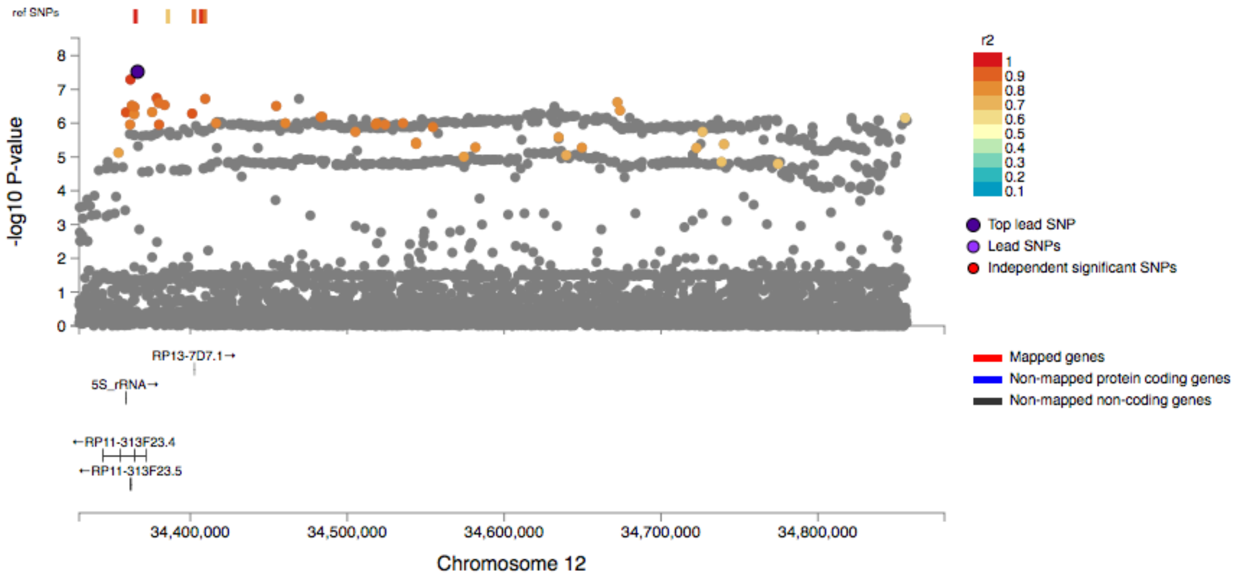

rs56374431

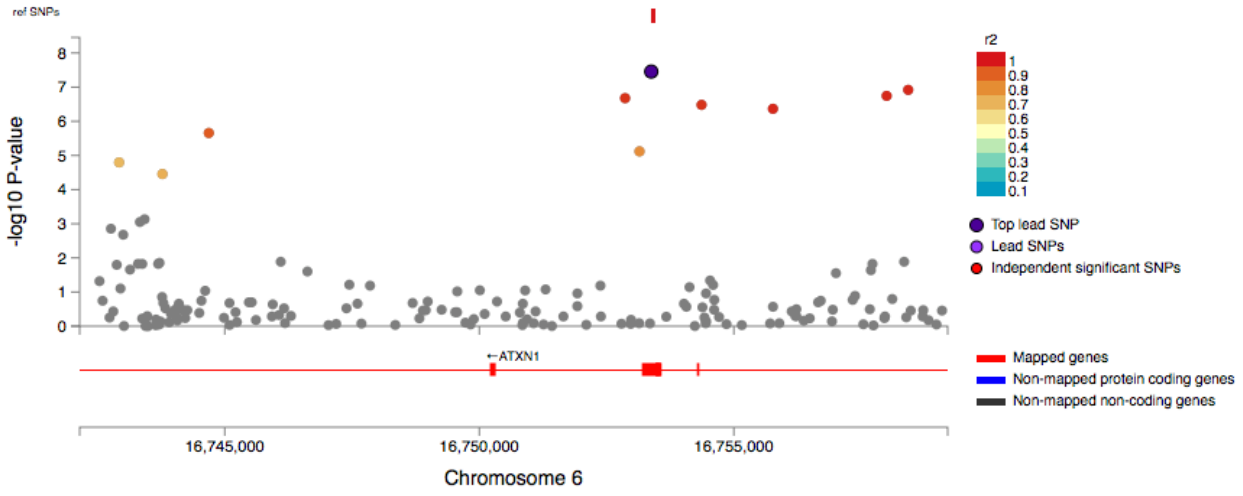

rs1345777

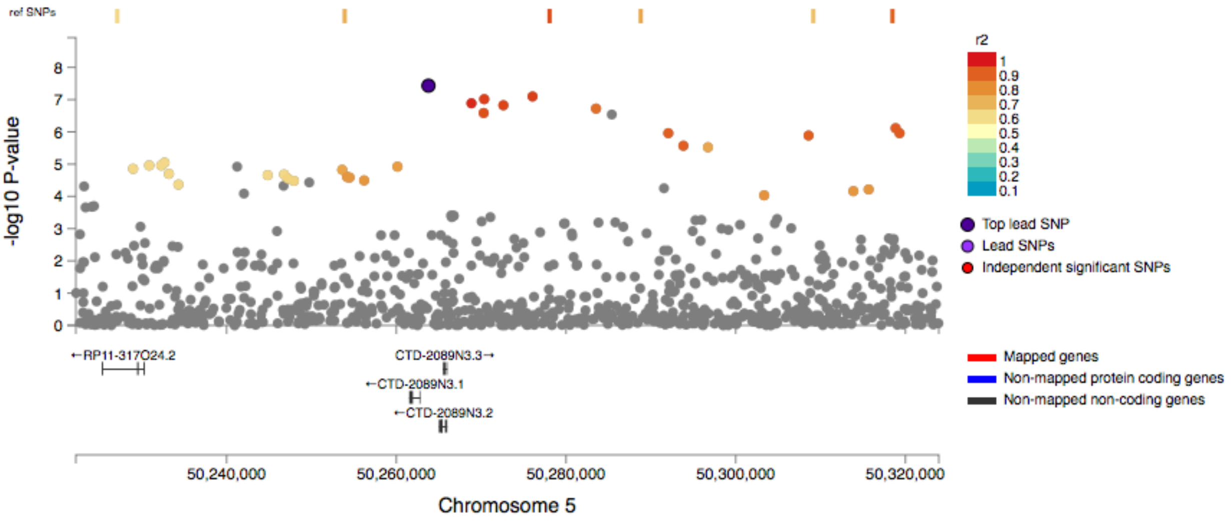

rs6870152

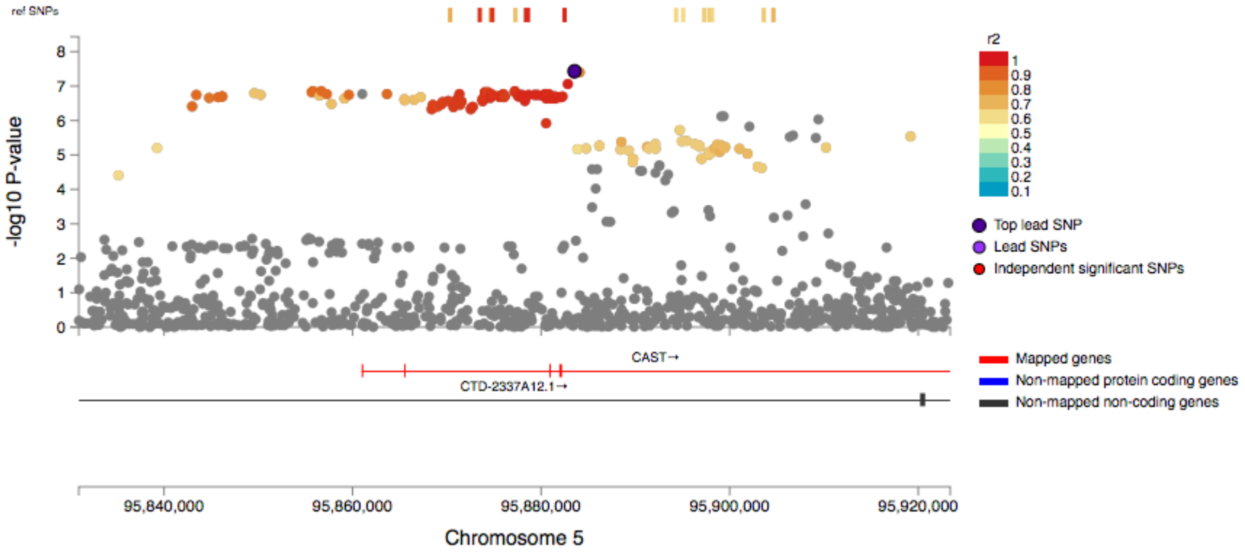

rs17818263

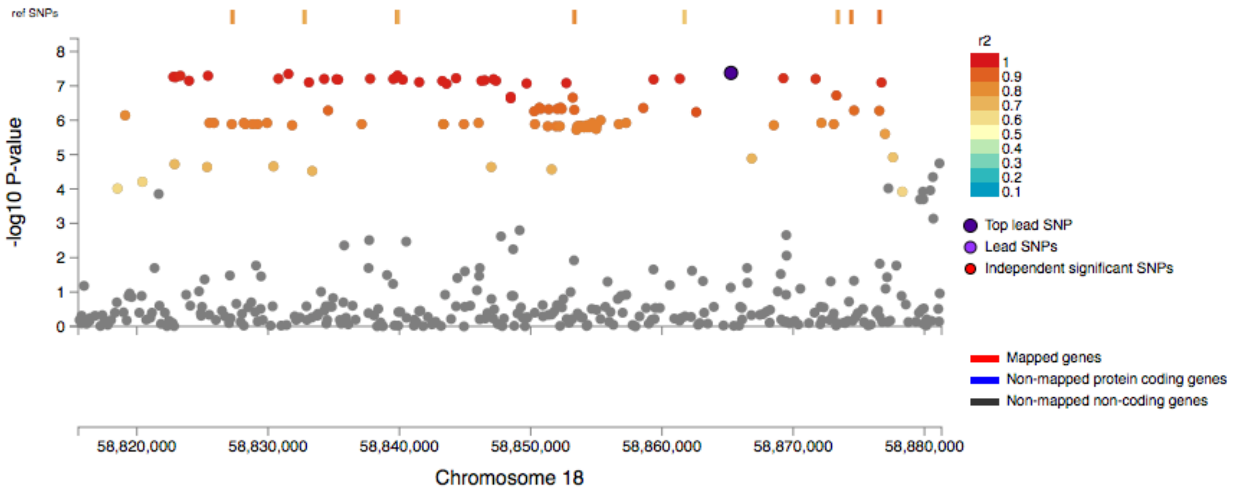

rs2320925

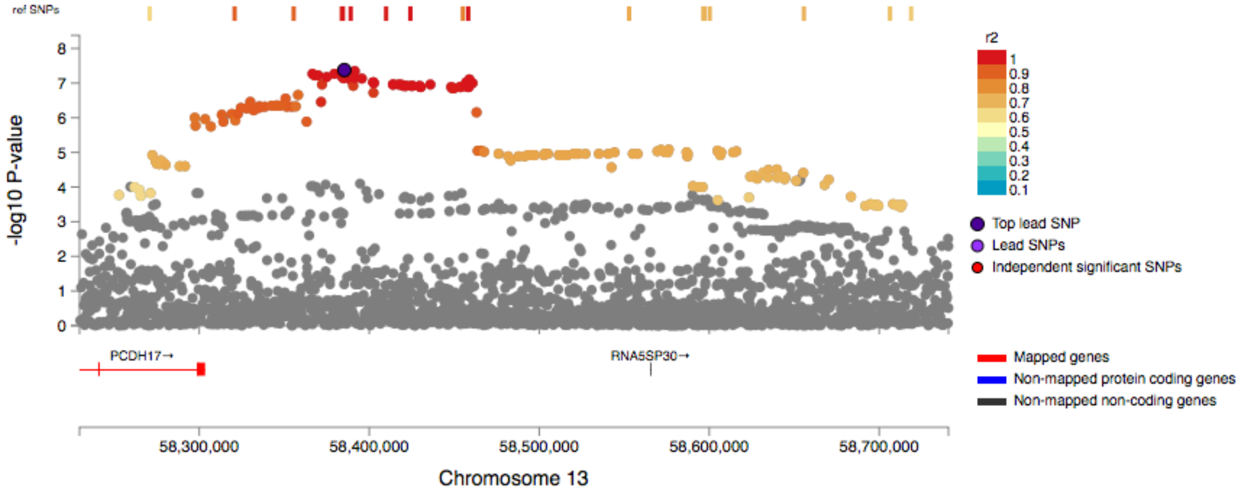

rs779429062

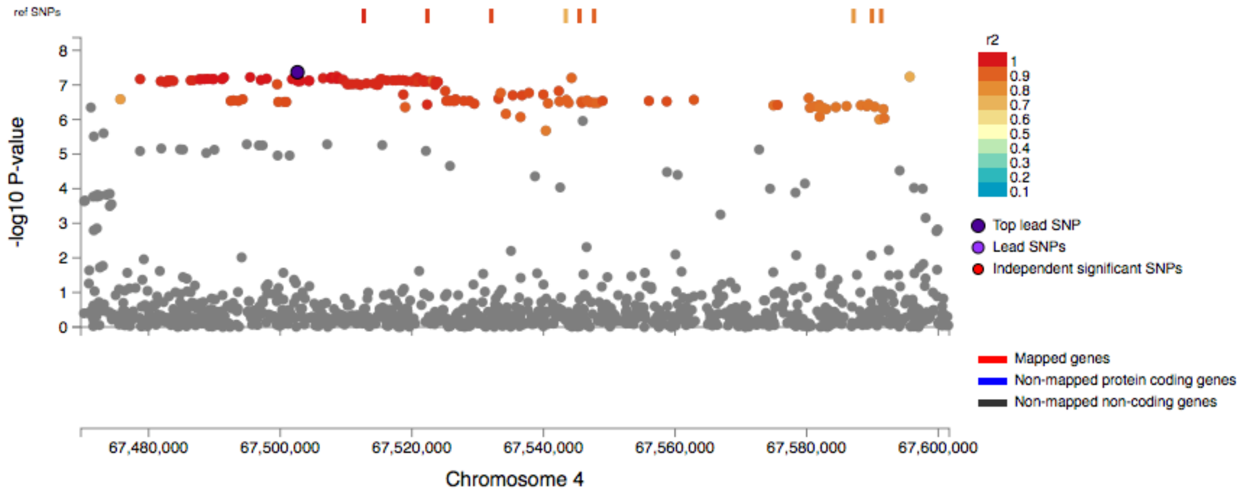

Supplement: Supplementary file 4 — Regional association plots [file 41398_2020_688_MOESM4_ESM.pdf]
